# *Poasecunda* J. Presl (Poaceae): a modern summary of infraspecific taxonomy, chromosome numbers, related species and infrageneric placement based on DNA

**DOI:** 10.3897/phytokeys.110.27750

**Published:** 2018-11-05

**Authors:** Robert John Soreng, Lynn J. Gillespie

**Affiliations:** 1 Department of Botany, National Museum of Natural History, Smithsonian Institution, Washington, DC 20013-7012, USA National Museum of Natural History, Smithsonian Institution Washington United States of America; 2 Research and Collections Division, Canadian Museum of Nature, P.O. Box 3443, Station D, Ottawa, Ontario K1P 6P4, Canada Research and Collections Division, Canadian Museum of Nature Ottawa Canada

**Keywords:** Apomixis, hybridisation, *
Poa secunda
*, Poaceae, polyploidy, reticulation, *
Secundae
*, taxonomy

## Abstract

*Poasecunda* J. Presl. s.l. is a morphologically highly variable bunchgrass that is a valuable forage species in western North America. There has been much controversy as to whether multiple taxa should be recognised and at what rank in this taxonomically challenging apomictic complex. Here we propose an infraspecific classification for *Poasecunda* of six varieties within two subspecies, *juncifolia* and *secunda.* New combinations are *P.secunda* vars. *ampla*, *gracillima*, *juncifolia*, *nevadensis* and *scabrella*. Conflicting plastid and nrDNA phylogenies show that P.sect.Secundae is of ancient hybrid origin. Based on this and its distinct morphology, the section is raised to the rank of subgenus. A key is presented for *P.secunda* infraspecies and closely related non-arctic species. Suppl. materials are provided of chromosome counts for *Secundae* taxa and D.D. Keck specimen annotations of taxa here included in *P.secunda*.

## Introduction

*Poasecunda* J. Presl. s.l. is a morphologically highly variable species found primarily in western North America. It is common to dominant in grasslands extending from Alaska to Northern Mexico and eastwards in the northern Great Plains and scattered more eastern locations to the Gaspé Peninsula in Quebec (Marsh 1952, Kellogg 1985a, Soreng 2007). The species is disjunct but not common in South America, occurring in Patagonian Argentina and Chile (presumed origin of the type collection of *P.secunda*). This perennial bunchgrass is a valuable forage species that greens up and flowers in early spring and is abundant across western grasslands, coastal chaparral, Great Basin steppe, uplands of the Mojave Desert and dry forests surrounding these and reaches into alpine meadows. It exhibits diverse ecotypes across this wide geographic and climatic range. *Poasecunda* s.l. is abundantly represented in herbaria across the United States; for example, the United States National Herbarium (US) has more than three very full herbarium cases of specimens for the United States and Canada. It is distinguished from most *Poa* species by its obscurely keeled lemmas, calluses often with a minute crown of hairs surrounding the base of the lemma (in subsp. *secunda*) and elongated spikelets (mostly 3.5–5 × longer than wide).

Differing taxonomies of the *Poasecunda* J. Presl complex continually appear in the literature. There are some 45 formal names applied to *P.secunda* s.l. Some taxonomists follow A.S. Hitchcock’s (1935, 1951) taxonomic revision in the *Manual of the Grasses of the United States*, in recognising his two informal groups of species, “Scabrellae” and “Nevadenses”. In the former group, Hitchcock included *P.scabrella* (Thurb.) Benth. ex Vasey, *P.gracillima* Vasey, *P.secunda* and *P.canbyi* (Scribn.) Howell; in the latter group *P.nevadensis* Vasey ex Scribn., *P.curtifolia* Scribn., *P.juncifolia* Scribn. and *P.ampla* Merr. Most of these taxa are still often recognised as species, subspecies or varieties (e.g. Keck 1950, 1959, Hitchcock et al. 1969, Dorn 1977, 1988). Keck (1950, 1959) additionally recognised *P.tenerrima* Scribn., a serpentine endemic of the Sierra Nevada foot hills and *P.incurva* Scribn. & T.A. Williams, a western sub-alpine/alpine element, within the Scabrellae group. Keck (1950, 1959) furthermore treated North American *P.secunda* as *P.sandbergii* Vasey, restricting *P.secunda* to South America and Hitchcock et al. (1969) accepted this split. Arnow (1981) concluded *P.secunda* was the correct name for plants of the *P.sandbergii* form. Hitchcock (1935, 1951) included *P.sandbergii* and *P.incurva* in *P.secunda*. Marsh (1950, 1952) and Kellogg (1985a, b) quite independently lumped most of these taxa in *P.secunda* without any infraspecies (followed by Lesica 2012, for Montana). Kellogg’s work, based on morphometric analyses, separated *P.curtifolia*; Marsh maintained that and also *P.tenerrima*. Soreng (1991b) divided P.secunda s.l. into two subspecies, subsp. secunda and subsp. juncifolia (Scribn.) Soreng, corresponding to the taxa in the Scabrellae and Nevadenses groups, respectively and accepted *P.curtifolia* and *P.tenerrima*. Dorn (1988) accepted as full species the two subspecies that Soreng recognised and established a few varieties within those. Skinner (2010) followed the subspecies split, but provided separate pages for each of the common names that Hitchcock (1951) applied to species aligned within the two subspecies: Sandberg bluegrass (P.secundavar.secunda), Sandberg bluegrass [P.secundavar.incurva (Scribn. & T.A. Williams) Beetle], Pine bluegrass (*P.scabrella*), Canby bluegrass (*P.canbyi*), Nevada bluegrass (*P.nevadensis*), Big bluegrass (*P.ampla*) and Alkali bluegrass (*P.juncifolia*). Several cultivars correspond to these different forms (Alderson and Sharp 1995). Thus, there appears to be a consistent desire and effort to maintain some or all of the diversity of forms commonly recognised, at some taxonomic rank.

Molecular (Patterson et al. 2005) and cytogenetic (Hiesey and Nobs 1982) studies show that *P.secunda* s.l. is a highly complex, apomictic species. Apomixis, although it is facultative to varying degrees (Clausen 1961, Kellogg 1987), is apparently the main mode of reproduction in *P.secunda* (Kellogg 1987). This has led to the production of various “strains”, ecotypes and races that are fairly monomorphic, but also to much intermediacy. Taxonomy in apomictic complexes is notoriously difficult, as is evident in the case of *P.secunda* s.l. However, we concur with Marsh (1952) and Kellogg (1985a, b) that the rank of species is not justifiable for most of the Scabrellae and Nevadenses taxa.

*Poasecunda* s.l. belongs to P.sect.Secundae, a primarily North American section of about eight species (Soreng 1991a, b, 1998, 2007, Soreng et al. 2003a, b, Gillespie et al. 2007). Soreng (1991a) established sect. Secundae with two subsections to accommodate species that share similar, apparently plesiomorphic Poeae traits, along with a derived chloroplast type (Soreng 1990) and suggested the section was of hybrid origin (Soreng 1990, 1991a, b). Our phylogenetic studies, based on plastid and nuclear ribosomal DNA, demonstrate that sect. Secundae is reticulate in origin (Gillespie et al. 2008, Cabi et al. 2017, Soreng et al. 2017). *Secundae* species exhibit several atavistic traits (Soreng 1991a, Soreng et al. 2015) that are otherwise odd in *Poa*, including; a crown of hairs around the base of the callus, upper culm leaf sheaths margins often free more than 80–90% of their length and lemmas that are often somewhat rounded on the back. These traits are common in genera outside of *Poa* and have led to many names for *Secundae* taxa being published in *Atropis*, *Glyceria* and *Puccinellia*. *Secundae* species are tufted (rhizomes occur in some putative hybrids, see Species hybrids involving *Poasecunda* in the Taxonomy section) and perfect flowered, with anthers ranging from 1.2–3 mm long. Soreng (1991a, 2007) recognised two subsections: *Secundae* and *Halophytae* V.L. Marsh ex Soreng. In addition to *P.curtifolia*, *P.secunda* s.l. and *P.tenerrima*, subsectionSecundae also includes two Arctic species, *P.ammophila* A.E. Porsild and *P.hartzii* Gand. (Soreng 1991b) and is characterised by elongate, weakly compressed spikelets and lemmas rounded on the back. Species of subsect. Halophytae (including *P.napensis* Beetle, *P.stenantha* Trin., *P.unilateralis* Scribn. ex Vasey) commonly have distinctly keeled lemmas and sometimes a papilliate epidermis on pedicels and leaf blades (Soreng 1991a, b, 2007).

Our goals in this paper are: 1) to provide a current overview of the taxonomy of *P.secunda* s.l. and present an up-to-date infraspecific classification including new combinations for the forms often recognised as species and 2) to document what we know of the relationships and hybrid origin of *P.secunda* s.l. and sect. Secundae. We also provide a review of the cytology of *P.secunda* s.l. and other species in sect. Secundae.

## *Poasecunda* s.l.: a revised infraspecific classification

In the interest of facilitating land-managers, ecologists, plant breeders, seed-storage facilities and collections managers in maintaining the understanding of variations in morphological forms in *P.secunda* s.l. that are often recognised as species, we here provide varietal names within *P.secunda* s.l. subspecies. This would also maintain herbarium collections that are organised or understood along A. S. Hitchcock’s taxonomic concepts, in which the taxa can be viewed to correspond to ecotypes or ecologically adapted apomictic lineages within *P.secunda*. There is extensive intermediacy between these taxa and, thus, the rank of species is viewed as untenable (Marsh 1950, 1952, Kellogg 1985a, b, Soreng 1991b, 1993, 2007, Soreng et al. 2003a, b). RJS’s revised classification of *P.secunda* s.l. is presented in the Taxonomy section below.

**Figure 1.**
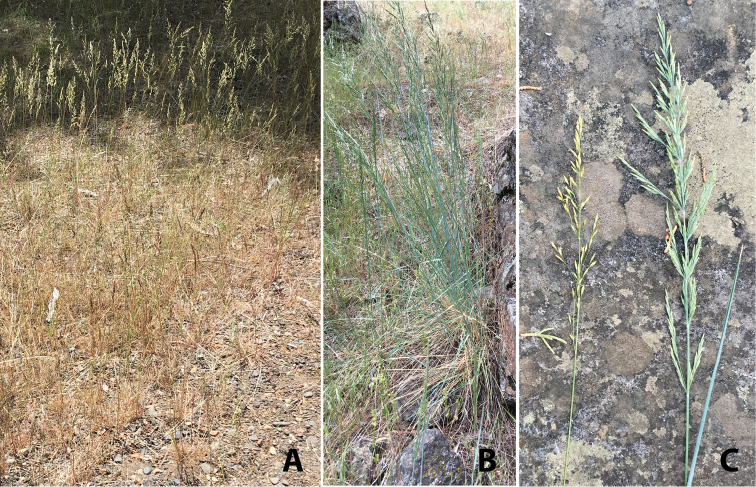
*Poasecunda* habit and panicles: **A**P.s.subsp.secundavar.secunda (*Soreng 9359*) **B**P.s.subsp.juncifoliavar.ampla (*Soreng 9358*) **C** Panicles of subsp. secundavar.secunda (left) and *juncifolia* var. ampla (right) (Photos. RJS, Deschutes River near Madras, Jefferson Co., Oregon).

**Figure 2.**
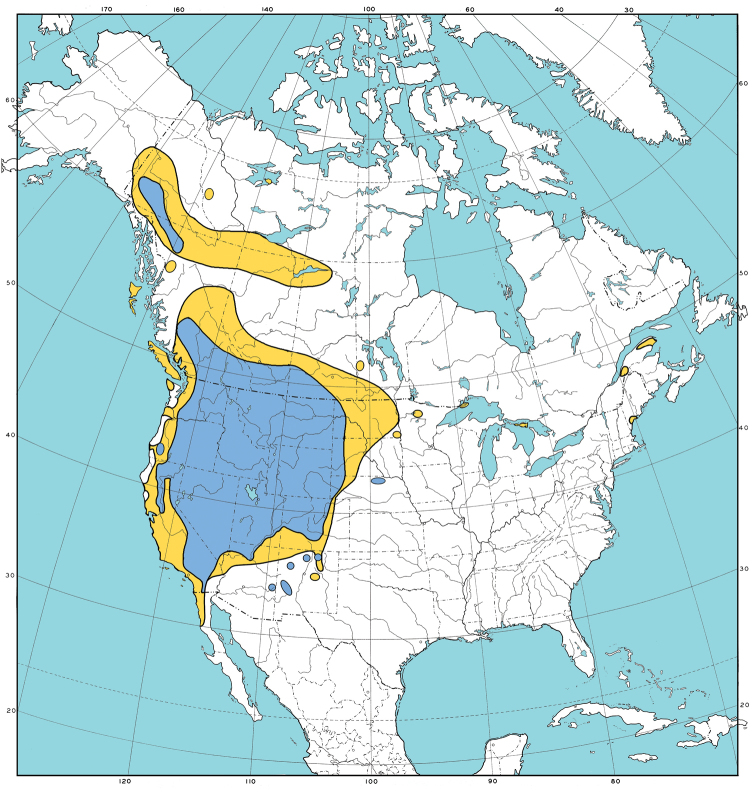
Distribution of *Poasecunda* subspecies in North America: subsp. secunda (yellow) ; subsp. juncifolia (blue).

As a whole, *P.secunda* s.l. is relatively easily split into two subspecies (*juncifolia* and *secunda*, Fig. 1), but finer distinctions are often inconstant and overlapping. Within subspecies *secunda*, it is a futile exercise to attempt to consistently separate *P.canbyi*, *incurva*, *sandbergii* and *secunda* forms and so these are all included in var. *secunda*. The epithet *gracillima* is often misapplied to plants RJS would classify as var. *secunda*. Both *gracillima* and *scabrella* are extreme forms that many botanists seem to think are worthy of taxonomic recognition at some level. Kellogg (1985a, b) concluded that the *P.ampla* form was the most distinct element within *P.secunda* s.l., but the difficulty noted in the literature (see Hitchcock et al. 1969) of separating that from subspecies juncifolia s.s., leads RJS to treat it as a variety in the subspecies. Range maps of the two *P.secunda* subspecies are provided in Fig. 2.

An exhaustive summary of the nomenclature, protologues and types for the more than 45 names included in *P.secunda* s.l. is beyond the scope of the present paper, but names of synonyms are listed. Hitchcock’s and some other historical synonymies for the nominal taxa can be found in his widely available "Manual of the Grasses of the United States" (Hitchcock 1951) and on-line: http://tropicos.org. See also the "Catalogue of New World Grasses" (Soreng et al. 2003a, and on-line Soreng et al. 2003b: http://tropicos.org/NameSearch.aspx?projectid=10) for synonymy for the two subspecies accepted.

A compilation of chromosome numbers for *Poasecunda* s.l. and other species in sect. Secundae (*P.hartzii*, *P.napensis*, *P.stenantha*, *P.tenerrima* and *P.unilateralis*) is presented in Suppl. material 1: Table S1. This shows that *Poasecunda* s.l. and all other taxa in sect. Secundae (for which chromosome numbers are known) are polyploid with a hexaploid base chromosome number of 2*n* = 42. There are many higher numbers, particularly in *P.secunda* (2*n* = 42 to 105 or 106; some 140 counts). Subsp. *secunda* has a mode at 2*n* = 84, while subsp. juncifolia has a mode at 2*n* = 63 (2n = 42 to ca. 100) (Soreng 1991b). Maintenance of a dysploid - nanaploid mode of ca. 2*n* = 63 in subsp. juncifolia suggests this subspecies is highly apomictic.

Suppl. material 2 provides Keck’s annotations for each of the nominal taxa he accepted that are here included in *P.secunda*. David Daniels Keck (1903–1995) had a deep interest in *Poasecunda* s.l. and other western North American species of *Poa* and, in 1986, he kindly gave RJS his manuscript on those, as well as his extensive specimen annotation lists for *Poa* of North America. He worked at the Carnegie Institute of Washington, at Stanford University, with J. Clausen and W. Hiesey for more than two decades (up to 1950), on the nature of species. He ceased his work on the lists by 1958 when he retired from the New York Botanical Garden. His lists focused on western Continental United States species, but included some mainly non-arctic Alaskan and Canadian and Mexican (Baja California) records, along with representative records of eastern United States species. Keck’s annotations represent hundreds of historical collections widely distributed in herbaria as vouchers for *P.secunda* infraspecies. His annotations are considered to be sound by RJS, although here we recognise the taxa at the rank of variety. Copies of the full lists are stored in the reprint files in the Grass Lab in the Department of Botany, Smithsonian Institution. The lists are reproduced here in a semi-searchable form.

## Phylogenetic relationships of *Poasecunda* s.l. and sect. Secundae

### Methods

Phylogenetic analyses were performed on 78 samples (73 *Poa* and five outgroups) (Suppl. material 3: Table S2.) using sequences of three plastid markers (matK, rpoB-trnC and trnT-trnL-trnF) and two nuclear ribosomal DNA (nrDNA) markers (ITS and ETS). Methods follow Soreng et al. (2017) and clade designations follow Soreng et al. (2010, 2017) (in the text, bold capital letters represent plastid types, bold small capital letters represent nrDNA types). Bayesian and maximum parsimony (MP) analyses were performed on the separate nuclear and plastid datasets. Branches having MP bootstrap support (BS) > 85% and Bayesian posterior probability (pp) support > 95% were considered strongly supported. See the following publications for genotype notation in *Poa*: Soreng et al. (2010, 2017), Nosov et al. (2015), Cabi et al. (2016, 2017).

### Results

**Figure 3.**
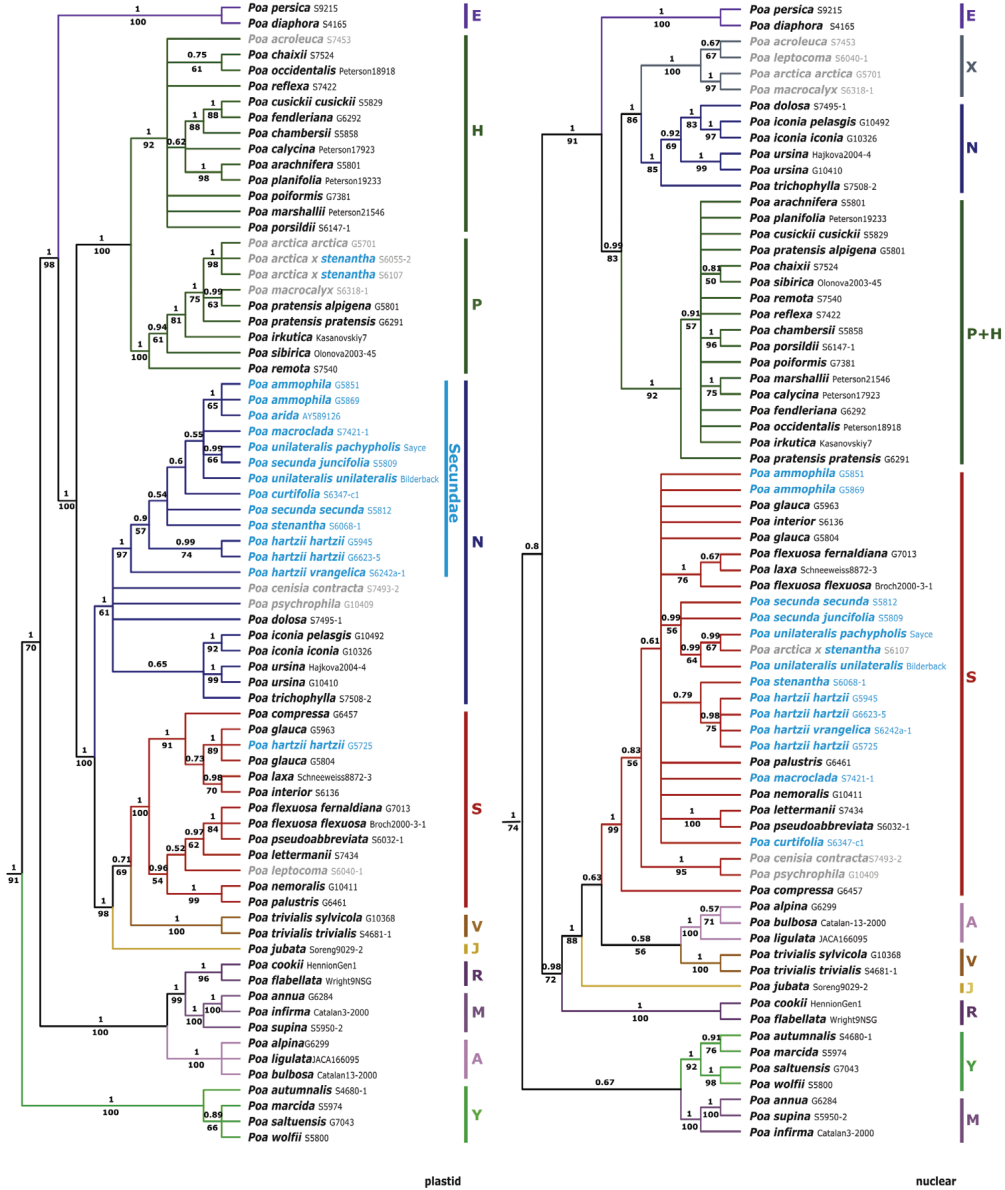
Bayesian 50% majority rule consensus trees of *Poa* based on plastid (trnT-trnL-trnF, rpoB-trnC, MatK) data (left) and nrDNA ITS and ETS data (right). Bayesian posterior probabilities are shown above branches, maximum parsimony bootstrap values below branches. Outgroups are not shown. Major clades are indicated by colour and capital letters. Taxa shown in blue belong to P.subg.Secundae; those in grey are other taxa of putative hybrid origin that belong to different major clades in plastid and nrDNA trees.

Separate plastid and nuclear Bayesian trees are presented in Fig. 3 (summary statistics are given in Suppl. material 4: Table S3). In the plastid analysis *Secundae* members form a strongly supported subclade (pp = 1, BS = 97%) within the **N** clade (pp = 1, BS = 61%), whereas in the nuclear analysis members are intermixed with species of *P.* sects. *Abbreviatae* Nannf. ex Tzvelev, *Tichopoa* Asch. & Graebn. and *Stenopoa* Dumort. in the strongly supported **s** clade (pp = 1, BS = 99%). The postulated hybrids *P.arctica* R. Br. × *P.stenantha* (*Soreng 6055-2*, *6107*) resolved with *P.arctica* (pp = 1, BS = 98%) in the **P** clade (pp = 1, BS = 100%) in the plastid tree and with *P.unilateralis* in the **s** clade in the nuclear tree (pp = 0.99, BS = 64%) (*Soreng 6055-2* not included since sequence unreadable due to multiple sequence copies).

Relationships amongst taxa within *Secundae* are mostly poorly supported and not congruent between plastid and nuclear trees. *Poaunilateralis* subspecies and P.arctica×stenantha form a clade and these form a clade with both *P.secunda* subspecies in the nuclear tree. In contrast in the plastid tree, P.unilateralissubsp.pachypholis (Piper) D.D. Keck and P.secundasubsp.juncifolia are sister taxa, whereas conspecific subspecies are not together. *Poahartzii* formed a moderately supported subclade (pp = 0.98, BS = 0.75) within the **s** clade in the nuclear tree, but one of four samples (*Gillespie 5725*) resolved outside the **N** clade, amongst *P.glauca* Vahl samples in the **S** clade, in the plastid tree. *Poamacroclada* Rydb., currently considered a synonym of *P.stenantha* (Soreng et al. 2003b, 2007), resolved in *Secundae* but did not resolve with the latter species in either tree.

## Discussion

The whole sectionSecundae is shown to be of reticulate origin (**Ns** plastid / nrDNA genotype combination) consistent with our previous results based on fewer samples (Gillespie et al. 2008, Cabi et al. 2017, Soreng et al. 2017). All sampled taxa of the section have the **N** plastid genotype marker that is otherwise known only from Europe and SW Asia (sect. Nanopoa J. R. Edm. and unclassified species) and the **s**nrDNA genotype of subg. Stenopoa (Dumort.) Soreng & L.J. Gillespie. While it remains a mystery how the **N** plastid type came to North America, it is evident that the **s**nrDNA type came from P.subg.Stenopoa (**Ss** genotype combination), either from sect. Stenopoa or, as we predict, from the primarily western North American sect. Abbreviatae (where *P.hartzii* was historically placed; see notes in Soreng 1991b) or possibly from the morphologically related Russian P.sect.Kolymenses Prob. (still lacking DNA data). Since **N** genotypes are otherwise absent from South America and **S** genotypes are otherwise rare there, we believe *Secundae* (P.secundasubsp.secunda, subsp. juncifolia and *P.stenantha*) arrived there secondarily by long distance dispersal from North America.

This ancient hybrid origin, together with its unusual morphology, make P.sectSecundae rather difficult to place within *Poa.* To better accommodate P.sect.Secundae within the infrageneric classification of *Poa*, we here raise the section to the rank of subgenus (see Taxonomy section below).

Curiously, *Poacenisia* All. and *P.psychrophila* Boiss. & Heldr. (P.sect.Cenisiae Asch. & Graebn.) also have an **Ns** genotype combination. This species group is confined to alpine habitats in central and southern Europe and Anatolian Turkey. The taxa are rhizomatous, with extravaginal branching, have a strongly keeled lemma and a dorsal tuft of cobwebby hairs (web) on the callus lemma. Morphological characteristics lead us to hypothesise that this group of species is derived from a cross between **Nn** taxa of Europe and **Ss** taxa from P.subg.Stenopoasect.Stenopoa, whereas we predict *Secundae* originated from a cross with P.subg.Stenopoasect.Abbreviatae**Ss** taxa. The **N** and **s** genotypes are slightly different between *Secundae* and *Cenisiae*, but if they prove to have a common origin, sect. Cenisiae may be better placed within subgenus Secundae than with species they are usually associated with in P.sect.Malacanthae (Roshev.) Olonova (type: *P.malacantha* Kom.), which exhibit **Px** genotypes (e.g. *P.arctica*, *P.macrocalyx* Trautv. & C.A. Mey., *P.smirnowii* Roshev.).

*Poahartzii*, an Arctic species distributed from Wrangel Island across the North American Arctic to Svalbard, represents an example in *Secundae* of more recent secondary reticulation. hartzii was shown previously (and again here) to include individuals with two different plastid types (**N** and **S**), interpreted as a case of recent and localised chloroplast introgression from *P.glauca* (Gillespie et al. 1997, 2007, Gillespie and Boles 2001). The sample with **S** plastid type, clearly has a *P.hartzii*nrDNA type.

*Poaarida* Vasey, a rhizomatous species of the Great Plains and eastern slopes of the Rocky Mts., was suggested by Soreng (2007) to have arisen from hybridisation of *P.secunda* and some element of P.sect.Poa. The species appears to have the typical *Secundae***Ns** genotype; it is shown here to have an **N** plastid genotype, whereas its nrDNA type is likely to be the **s** type as cloned nuclear DNA genes of *P.arida* track with *P.secunda* (Patterson et al. 2005). So, the postulated origin of rhizomes in *P.arida* deriving from Poasect.Poa may be wrong, but deeper genetic analyses would be more convincing. Two collections, *Soreng & Soreng 6055* and *6107*, from Alaska, are viviparous plants identified by RJS as *P.arctica* × *P.stenantha*. Both form a clade with *P.arctica* (sect. Malacanthae) in the plastid tree. In the nuclear tree, while the single sample included (*6107*, from the Alaska Range) resolves amongst *Secundae* taxa, it forms a clade with *P.unilateralis* and these with *P.secunda*, rather than *P.stenantha*. Based on geography, the paternal parent could not be *P.unilateralis* (coastal Washington to California) and is unlikely to be *P.secunda* (occurs in SE Alaska, but not in the Alaska Range); the only co-occurring *Secundae* is *P.stenantha*. Further research is needed, including deeper sampling in *P.stenantha* to determine the precise origin of this common viviparous Alaskan form.

Given that P.subg.Secundae is an apomictic, polyploid (often with high and odd sets of seven and further dysploid chromosome numbers), hybridising complex of reticulate origin, it is not surprising that the detected phylogenetic structure within the group is mostly not well resolved nor supported. Neither the plastid nor nrDNA trees support the current division into two subsections, *Secundae* and *Halophytae*. Several clades that are weakly to moderately supported may be informative of possible changes to the taxonomy. One example is the status of *P.macroclada*. This taxon was considered as a southern variant of *P.stenantha*, subsequently tentatively treated as a separate species in P.sect.Stenopoa (Soreng et al. 2003a) and then most recently as a synonym of *P.stenantha* (Soreng et al. 2003b, Soreng 2007). Our data suggest that it may be a distinct species in subg. Secundae. Such taxonomic changes may be considered in the future, but we feel making changes within *Secundae*, based on the current phylogenetic trees, to be premature, especially given the small sample set within *P.secunda* s.l. and the nature of the beast.

## Taxonomy

### Poa subg. Secundae

Taxon classificationPlantaePoalesPoaceae

 (V.L. Marsh ex Soreng) Soreng & L.J. Gillespie, comb. et stat nov.

urn:lsid:ipni.org:names:77191590-1

#### Basionym.

Poasect.Secundae V.L. Marsh ex Soreng, Syst. Bot. 16(3): 511, 523. 1991a.

#### Type species.

*Poasecunda* J. Presl.

#### Species included.

*Poaammophila*, *P.curtifolia*, *P.hartzii*, *P.napensis*, *P.secunda*, *P.stenantha* (including *P.macroclada*?), *P.tenerrima*, *P.unilateralis*.

#### Notes.

The sectionSecundae was originally suggested to belong to P.subg.Poa (Soreng 1991a), but, in subdividing the genus into more subgenera based on our plastid phylogeny (Gillespie et al. 2007), the section was moved to subg. Stenopoa (Dumort.) Soreng & L.J. Gillespie. We subsequently discovered that the set of plastid *Secundae* genotypes resolved in a clade with three European diploid species with **Nn** genotypes, but the nrDNA genotypes of Secundae were like those of subg. Stenopoa (**Ss**) (Cabi et al. 2017, Soreng et al. 2017 and results presented above). The apparently ancient reticulate origin of the group, along with its odd morphology within *Poa*, now leads us to raise the section to the rank of subgenus.

### Poa secunda

Taxon classificationPlantaePoalesPoaceae

J. Presl, Reliq. Haenk. 1(4–5): 271, 1830

#### Range.

CANADA: Alberta, British Columbia, Manitoba (sw), Northwest Territories (sw), Ontario (Manitoulin Island), Quebec (Gaspe Peninsula), Saskatchewan, Yukon (s). UNITED STATES: Alaska (se interior border), Arizona (n), California, Colorado, Idaho, Montana, North Dakota, Nevada, New Mexico (n), Oregon, South Dakota (w), Utah, Washington, Wyoming, with outlying populations in Nebraska (w), Oklahoma (panhandle), Michigan (Isle Royal) and sporadic in Illinois and Maine, Massachusetts.

The geographic range of *P.secunda* in North America north of Mexico is mapped in Fig. 2. Subspecies *secunda* occurs throughout the species range and also reaches into Baja California Norte, Mexico (Fig. 2A). The range of subspecies juncifolia is almost completely within that of subsp. *secunda*, except for several scattered localities in Arizona, New Mexico and Nebraska; it is absent from Mexico and mostly absent from the Pacific Coast and coastal mountains (Fig. 2B). The ranges of varieties are broadly overlapping within their subspecies. Both subspecies have disjunct populations in the Patagonian Andes of Argentina and Chile.

### Poa secunda subsp. juncifolia

Taxon classificationPlantaePoalesPoaceae

(Scribn.) Soreng, Phytologia 71(5): 401. 1991b [1992]

#### Basionym.

*Poajuncifolia* Scribn., Bull. Div. Agrostol., U.S.D.A. 11: 52, pl. 8, 1898.

### Poa secunda subsp. juncifolia var. ampla

Taxon classificationPlantaePoalesPoaceae

(Merr.) Soreng, comb. et stat nov.

urn:lsid:ipni.org:names:77191591-1

Figs 1B, C , 3A

#### Basionym.

*Poaampla* Merr., Rhodora 4(43): 145, 1902.

#### Synonyms.

*Poaampla* Merr., *P.confusa* Rydb., P.juncifoliavar.ampla (Merr.) Dorn, *P.laeviculmis* T.A. Williams, *P.truncata* Rydb.

#### Habitat and range.

Open upland forests, mountain steppe, generally in light, well-drained soils to somewhat heavy soils. Range of the subspecies (Fig. 2B), but mostly absent from west side of the Sierra Nevada and westwards. Minor outlying occurrences in Arizona and New Mexico of subsp. juncifolia (Fig. 2B) mostly represent var. ampla (Soreng 1985), likely introduced by seeding.

#### Chromosome numbers.

Numbers reported as *P.ampla*: 2*n* = 61, 62(x3), 63 (x11), ≈ 63 (x3), 63–64 (x2), 64 (x6), ≈ 65, 70–71, ≈ 97, ≈ 100.

### Poa secunda subsp. juncifolia var. juncifolia

Taxon classificationPlantaePoalesPoaceae

(Scribn.) Soreng, comb. et stat nov.

urn:lsid:ipni.org:names:77191594-1

Fig. 4B.

#### Basionym.

*Poajuncifolia* Scribn., Bull. Div. Agrostol., U.S.D.A. 11: 52, pl. 8, 1898.

#### Synonyms.

*Poabrachyglossa* Piper, P.fendlerianavar.juncifolia (Scribn.) M.E. Jones, *P.juncifolia* Scribn., P.juncifoliasubsp.juncifolia, P.juncifoliasubsp.porteri D.D. Keck (?), P.nevadensisvar.juncifolia (Scribn.) Beetle.

#### Notes.

The type and other material identified as Poajuncifoliasubsp.porteri by Keck combine pubescent lemmas with narrow panicles, firm blades and short ligules on lateral shoots and appear to RJS to be intermediate in form between varieties *juncifolia* and *secunda* (*canbyi* form).

#### Habitat and range.

Pine forests and steppe, riparian and alkali meadows, in well-drained to poorly-drained, light to heavy, often alkaline or saline soils. Range of the subspecies (Fig. 2B), but infrequent in the California Floristic Province and south-western states.

**Figure 4.**
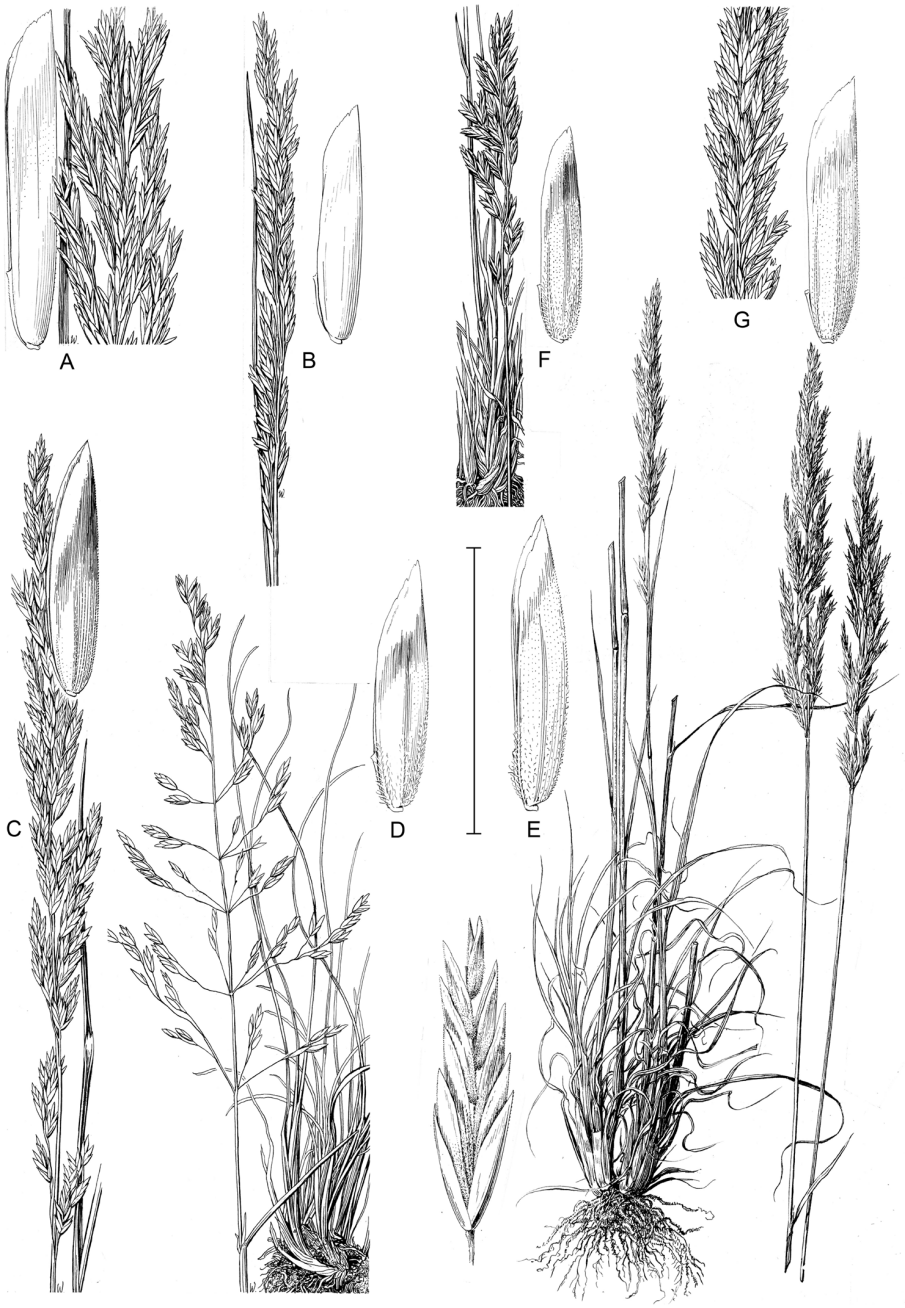
*Poasecunda* infraspecies illustrations (reproduced from Hitchcock 1935): **A**P.s.var.ampla panicle and floret **B**P.s.var.juncifolia panicle and floret **C**P.s.var.nevadensis panicle and floret **D**P.s.var.gracillima habit, panicle and floret **E**P.s.var.scabrella habit, panicles, spikelet and floret **F, G**P.s.var.secunda habit, panicle and floret variations **A, B, C** = subsp. juncifolia**D, E, F, G** = subsp. *secunda*. Scale bar: 5 mm for florets, 10 mm for spikelet, 5 cm for habits and panicles (10 cm for **E** habit and panicles).

#### Chromosome numbers.

Numbers reported as *P.juncifolia*: 2*n* = 42, 60, 62, 63, 63–64, 78, 84. The one 2*n* = 42 count was originally published by Hartung (1946) as *P.cusickii*, but RJS re-determined the California voucher as P.secundasubsp.juncifoliavar.juncifolia.

### Poa secunda subsp. juncifolia var. nevadensis

Taxon classificationPlantaePoalesPoaceae

(Vasey ex Scribn.) Soreng, comb. et stat nov.

urn:lsid:ipni.org:names:77191595-1

Fig. 4C

#### Basionym.

*Poanevadensis* Vasey ex Scribn., Bull. Torrey Bot. Club 10: 66, 1883.

#### Synonyms.

*Atropisnevadensis* (Vasey ex Scribn.) Beal, *Atropispauciflora* Thurb., *Paniculariathurberiana* Kuntze, *Poanevadensis* Vasey ex Scribn., *Poapauciflora* (Thurb.) Benth. ex Vasey, Poatenuifoliavar.scabra Vasey ex Scribn. (nom. inval.), *Poathurberiana* (Kuntze) Vasey, *Puccinellianevadensis* (Vasey ex Scribn.) Ponert.

#### Habitat and range.

Open forests and steppe, in light, well-drained to heavier soils. Range of the subspecies (Fig. 2B), but mostly absent from west side of the Sierra Nevada and westward. Collected once in New Mexico (Soreng 1985).

#### Chromosome numbers.

Numbers reported as *P.nevadensis*: 2*n* = 62 (x2), 62–63, 63 (x7), 64, 64–66 (x2), ≈ 65, 70.

### Poa secunda subsp. secunda

#### Poa secunda subsp. secunda var. gracillima

Taxon classificationPlantaePoalesPoaceae

(Vasey) Soreng, comb. et stat nov.

urn:lsid:ipni.org:names:77191596-1

Fig. 4D

##### Basionym.

*Poagracillima* Vasey, Contr. U.S. Natl. Herb. 1(8): 272, 1893.

##### Synonyms.

Poagracillimavar.gracillima, *P.gracillima* Vasey, *P.invaginata* Scribn. & T.A. Williams.

##### Habitat and range.

Open forests, moist cliffs and rocks and subalpine to alpine meadows, in well-drained acid soils that are consistently moist through the growing season of the variety. Pacific Northwest and northern California.

##### Chromosome numbers.

Numbers reported as *P.gracillima*: 2*n* = 81, ≈ 81, ≈ 84, 86, ≈ 91.

#### Poa secunda subsp. secunda var. scabrella

Taxon classificationPlantaePoalesPoaceae

(Thurb.) Soreng, comb. et stat nov.

urn:lsid:ipni.org:names:77191598-1

Fig. 4E

##### Basionym.

*Atropisscabrella* Thurb., Bot. California 2: 310–311, 1880. *Poascabrella* (Thurb.) Benth. ex Vasey, Grass. U.S. 42, 1883.

##### Synonyms.

*Atropiscalifornica* Munro ex Thurb., *Atropisscabrella* Thurb., *Paniculariascabrella* (Thurb.) Kuntze, *Poaacutiglumis* Scribn., *Poacalifornica* (Munro ex Thurb.) Scribn., *Poacapillaris* Scribn., *Poanudata* Scribn., *Poaorcuttiana* Vasey, *Poascabrella* (Thurb.) Benth ex Vasey, *Puccinelliascabrella* (Thurb.) Ponert, *Sclerochloacalifornica* Munro ex Benth.

##### Habitat and range.

Open pine forests, coastal scrub and coastal and Central Valley grasslands, in well-drained or heavier soils. Mainly in the California Floristic Province, but extending northwards in the Pacific North West and southeast into the Mojave Desert, where it is largely replaced by var. *secunda*.

##### Chromosome numbers.

Numbers reported as *P.scabrella*: 2*n* = 44+f, 61–63, ≈ 62, 63 (x4), 64, ≈ 66, ≈ 68, 81 (x2), 82 (x3), 84 (x10, ≈ 84, 84+f, 86, ≈ 86 (x2), ≈ 88, ≈ 91, 104.

#### Poa secunda subsp. secunda var. secunda

Taxon classificationPlantaePoalesPoaceae

Figs 1A, C , 4F, G

##### Synonyms.

*Airabrevifolia* Pursh, *Airamissurica* Spreng. ex B.D. Jacks., *Airopsisbrevifolia* (Pursh) Roem. & Schult., *Atropiscanbyi* (Scribn.) Beal, *Atropislaevis* Beal, Atropislaevisvar.rigida Beal, *Atropistenuifolia* Thurb., Atropistenuifoliavar.stenophylla Vasey ex Beal (*incurva* form), *Festucaoregona* Vasey, *Festucapatagonica* Phil., *Festucaspaniantha* Phil., *Glyceriacanbyi* Scribn., *Panicularianuttalliana* Kuntze, *Poaandina* Nutt. ex S. Watson, *Poabuckleyana* Nash, Poabuckleyanavar.buckleyana, Poabuckleyanavar.elongata (Vasey) M.E. Jones, Poabuckleyanavar.sandbergii (Vasey) M.E. Jones, Poabuckleyanavar.stenophylla (Vasey ex Beal) M.E. Jones, *Poacanbyi* (Scribn.) Piper, *Poafulvescens* Trin., Poagracillimavar.saxatilis (Scribn. & T.A. Williams) Hack., *Poahelleri* Rydb., *Poaincurva* Scribn. & T. A. Williams, *Poalaevigata* Scribn., *Poalaevis* Vasey, *Poaleckenbyi* Scribn., *Poalucida* Vasey, Poanevadensisvar.laevigata (Scribn.) M.E. Jones, Poanevadensisvar.leckenbyi (Scribn.) M.E. Jones, *Poasandbergii* Vasey, *Poasaxatilis* Scribn. & T.A. Williams (toward *gracillima* form), Poasecundavar.elongata (Vasey) Dorn (= *canbyi* form), Poasecundavar.incurva (Scribn. & T.A. Williams) Beetle, Poasecundavar.stenophylla (Vasey ex Beal) Beetle, Poastenanthavar.sandbergii (Vasey) B. Boivin, *Poatenuifolia* Buckley, *Poatenuifolia* Nutt. ex S. Watson, Poatenuifoliavar.elongata Vasey, Poatenuifoliavar.oregona (Vasey) Vasey, *Poawyomingensis* Scribn., *Puccinelliacanbyi* (Scribn.) Ponert, *Puccinellialaevis* (Beal) Ponert

##### Notes.

There are various variety names included in the autonymic var. *secunda*. Variety *sandbergii* is *secunda* s.s.; vars. *elongata*, *leckenbyi*, *laevigata* and *rigida* represent the *canbyi* form; and vars. *incurva*, *saxatilis*, *buckleyana* and *stenophylla* represent the *incurva* form. Varieties *incurva* and *saxatilis* are generally subalpine to alpine with slightly open panicles; Hitchcock (1951) included them within *P.gracillima*, but Keck (1959 and annotations) treated them as *P.incurva*. Vars. *buckleyana* and *stenophylla* are intermediate to var. scabrella, but are too smooth to be included in the latter variety. Hitchcock (1951) equated *stenophylla* with var. gracillima, but we agree with Keck (Suppl. material 2) in equating it with the *incurva* form. *Poatenuifolia* Nutt. ex S. Watson (1871), nom. illeg., was described from heterogeneous original material (at US, distributed as *S. Watson 1318*): E. Humboldt Mts. = var. juncifolia; Virginia Mts. = var. secunda ; Diamond Mts. = var. scabrella. However, Watson also cited “*Poatenuifolia* Nutt., ms. In Herb.” The Nuttall specimen with that name is at PH (PH00020091) and *P.tenuifolia*Buckley (1862) is based on that specimen. Therefore, Watson’s taxon name is superfluous.

##### Habitat and range.

Open forests, steppe, and alpine, generally in light, well-drained soils. Range of the subspecies (Fig. 2B), but mostly replaced by var. scabrella on west side of the Sierra Nevada and westwards and in the Mojave Desert.

##### Chromosome numbers.

Numbers reported as *P.secunda* and avowed synonyms: 2*n* = 42, 56, ≈ 68, 70 (x2), ≈ 70 (x2), ≈ 74, ≈ 78 (x2), 81, 81, 82 (x2), 84 (x2), 84–88+II, ≈ 84, 85–87, 86 (x2), ≈ 87, ≈ 99, 104. The one count of 2*n* = 42 was reported by Bowden (1961) originated from Alberta. Numbers originally reported as *P.canbyi*: 2*n* = 56, 70 (x4), ≈ 72, ≈ 82 (x2), ≈ 83, 84 (x4), ≈ 84 (x2), 85, ≈ 86. Keck (in Munz 1959) attributed higher numbers to *P.incurva*). Numbers secondarily reported as *P.incurva*: 2*n* = 90, 93, 94, 99, 105–106.

#### Key to *Poasecunda*, *P.curtifolia* and *P.tenerrima* (adapted from Hitchcock 1951) (non-Arctic taxa of P.subg.Secundae with rounded lemma keels)

**Table:** 

1	Lemmas more or less crisp-puberulent on the lower half or basal portion (sometimes obscurely so in P.secundavar.scabrella); ligules of lower culm usually well developed and acute to acuminate (short in *P.tenerrima*); tillers strictly intravaginal, cataphylls absent, prophylls well developed, mostly over 1 cm long; leaf blades commonly withering early, long-cells all or mostly fusiform and smooth-walled	**2**
–	Lemmas glabrous, smooth or scabrous (except in “P.juncifoliasubsp.porteri” form, but then plants from the plains of the eastern slope of the Rocky Mts.); ligules of lower culm and lateral shoot leaves truncate to rounded (acute in var. nevadensis); tillers intravaginal and sometimes extravaginal, the latter with cataphylls and reduced prophylls (mostly less than 2 mm long); leaf blades more or less persisting in form, long-cells mostly rectangular and sinuous-walled	**6**
2	Leaf blades short (mostly 1–3 cm long), (1–) 1.5–3 mm wide, flat, with prominent white, cartilaginous margins; plants of serpentine rocks in the Wenatchee Range of the Cascade Mts., Washington State	*** P. curtifolia ***
–	Leaf blades of various lengths and widths, but not short and flat, without prominent cartilaginous margins	**3**
3	Sheaths scabrous, at least on the margins; ligules scabrous; panicle branches scabrous, often densely so; plants mainly of California Floristic Province and Mojave Desert	**4**
–	Sheaths smooth; ligules smooth or lightly scabrous; panicle branches smooth or scabrous; plants mostly of the eastern slope of the western Cordilleras and eastward	**5**
4	Ligules of culm leaves well developed (2–6 mm long), acute to acuminate; blades filiform or broader; panicles branches capillary or thicker, appressed to ascending (rarely spreading); plants widespread; chromosomes 2*n* = 63 and higher	** P. secunda subsp. secunda var. scabrella **
–	Ligules of culm leaves short [0.5–1.5 (–2.5) mm long], truncate to obtuse (acute); blades filiform; panicle branches capillary, widely spreading; plants of serpentine barrens in central foothills of west slope of the Sierra Nevada; chromosomes 2*n* = 42	*** P. tenerrima ***
5	Panicles open, the branches spreading to patent, divergent more than 45° at anthesis and remaining open with spikelets absent in the lower half; plants of moist often shady places	** P. secunda subsp. secunda var. gracillima **
–	Panicles usually loosely to tightly contracted at maturity, branches sometimes ascending but branches finally divergent by less than 45°, spikelets from near the base or lower 1/3^rd^; plants mostly of more open places	** P. secunda subsp. secunda var. secunda **
6	Sheaths scabridulous; ligules elongated, acute, decurrent	** P. secunda subsp. juncifolia var. nevadensis **
–	Sheaths smooth; ligules of lower culm and basal leaf short, obtuse to truncate, not decurrent	**7**
7	Blades involute; plants of open riparian and alkali or saline meadows	** P. secunda subsp. juncifolia var. juncifolia **
–	Blades flat; plants of mountain meadows and forests	** P. secunda subsp. juncifolia var. ampla **

## Supplementary Material

XML Treatment for Poa subg. Secundae

XML Treatment for Poa secunda

XML Treatment for Poa secunda subsp. juncifolia

XML Treatment for Poa secunda subsp. juncifolia var. ampla

XML Treatment for Poa secunda subsp. juncifolia var. juncifolia

XML Treatment for Poa secunda subsp. juncifolia var. nevadensis

XML Treatment for Poa secunda subsp. secunda var. gracillima

XML Treatment for Poa secunda subsp. secunda var. scabrella

XML Treatment for Poa secunda subsp. secunda var. secunda

## Supplementary materials

Supplementary material 1 Table 1. Chromosome numbers in taxa of Poasubg.Secundae Data type: species data Explanation note: Chromosome numbers in taxa of Poasubg.Secundae, with RJS’ subspecies determinations and original determinations as published or found on herbarium sheets, literature reference, number of counts, voucher collection, country and state or province abbreviation and herbarium where deposited, if known. CI = Carnegie Institution. These numbers show the hexaploid nature of the species complex and the wide and differing ranges of chromosome numbers in each of the *P.secunda* subspecies. This is an open access article distributed under the terms of the CC0 Public Domain Dedication.Robert John Soreng, Lynn J. Gillespie

Supplementary material 2 David D. Keck’s annotations of taxa here included in *Poasecunda* Data type: species data Explanation note: The following taxa recognised by Keck, but included by us in *P.secunda* s.l., are given in the specimen annotation lists: *P.ampla*, *P.canbyi*, *P.gracillima*, *P.incurva*, *P.juncifolia*, *P.nevadensis*, *P.sandbergii* and *P.scabrella*. His lists focused on western Continental United States species but included some mainly non-arctic Alaskan, Canadian and Mexican (Baja California) records and some records of eastern United States species. Copies of the original typed lists are stored in the reprint files in the Grass Lab in the Department of Botany, Smithsonian Institution. Optical character recognition (OCR) was performed on the present selection to allow the lists to be searchable to a large degree. Keck’s annotations are considered to be sound and to represent hundreds of historical collections widely distributed in herbaria as vouchers for *P.secunda* infraspecies. We treat *P.ampla*, *P.juncifolia* and *P.nevadensis* as varieties of P.secundasubsp.juncifolia (vars. *ampla*, *juncifolia* and *nevadensis*, respectively) in our revised classification. The remaining taxa are treated as varieties of P.secundasubsp.secunda , as follows: var. gracillima (*P.gracillima*), var. scabrella (*P.scabrella*) and var. *secunda* (*P.canbyi*, *P.incurva*, and *P.sandbergii*). This is an open access article distributed under the terms of the CC0 Public Domain Dedication.Robert John Soreng, Lynn J. Gillespie

Supplementary material 3 Table 2. *Poa* and outgroup samples used in the phylogenetic analyses Data type: phylogenetic data Explanation note: *Poa* and outgroup samples used in the phylogenetic analyses with subgeneric classification (subtribe for outgroups), voucher information and GenBank Accession numbers for each of the five DNA regions (ITS, ETS, trnT-trnL-trnF, matK, rpoB-trnC). This is an open access article distributed under the terms of the CC0 Public Domain Dedication.Robert John Soreng, Lynn J. Gillespie

Supplementary material 4 Table 3. Data partition characteristics, summary statistics and models of the phylogenetic analyses Data type: phylogenetic data Explanation note: For each data partition (ITS, ETS, trnT-trnL-trnF, rpoB-trnC, MatK) and concatenated dataset (plastid and nuclear), the following are given: number of sequences, number of characters, number of parsimony informative (PI) characters, % parsimony informative characters, maximum parsimony tree length (L), number of most parsimonious trees, consistency index excluding uninformative characters (CI) and retention index (RI). Also given are the models used in the Bayesian analyses as determined using the Akaike information criterion (AIC) in jModeltest. This is an open access article distributed under the terms of the CC0 Public Domain Dedication.Robert John Soreng, Lynn J. Gillespie
