# Supplementary material for: Poasecunda J. Presl (Poaceae): a modern summary of infraspecific taxonomy, chromosome numbers, related species and infrageneric placement based on DNA
Source: PhytoKeys. 2018 Nov 5;(110):101–21. doi: 10.3897/phytokeys.110.27750 (PMC6232245; doi:10.3897/phytokeys.110.27750)
Supplement: Supplementary material 1 — Table 1. Chromosome numbers in taxa of Poasubg.Secundae [file phytokeys-110-101-s001.pdf]

Supplementary file I. Table 1. Chromosome numbers in taxa of *Poa* subg. *Secundae,* with original determination, literature reference, number of counts, voucher collection, Country and State or Province abbreviation, and herbarium where deposited if known. CI = Carnegie Institution.

Genus	Species	Rank	Subspecies	Subvariety	2n =	Original determination	Literature Reference	No. of counts reported	Voucher	Country-Region	Herbarium vouchered
Poa	*curtifolia*				2n = 42	*P. curtifolia*	Kellogg 1983	1	Kellogg 104	USA, WA	
Poa	*hartzii*	subsp.	*hartzii*		2n = 63	*P. hartzii*	Zhukova & Petrovsky 1972	1		RUSSIA far east	
Poa	*hartzii*	subsp.	*hartzii*		2n = 63-70	*P. hartzii*	Holmen 1952	1	Holmen	GREENLAND	
Poa	*hartzii*	subsp.	*hartzii*		2n = 70	*P. hartzii*	Jorgensen et al. 1958	2	JSW & JSW fig. 24	GREENLAND	
Poa	*hartzii*	subsp.	*hartzii*		2n = 70	*P. hartzii*	Löve & Löve 1975	3			
Poa	*hartzii*	subsp.	*hartzii*		2n = 70	*P. hartzii*	Petrovsky & Zhukova 1981	1		RUSSIA far east	
Poa	*hartzii*	subsp.	*vrangelica*		2n = 49	*P. vrangelica*	Zhukova & Petrovsky 1971 Zukova & Petrovsky 1972 Petrovsky & Zukova 1981	1		RUSSIA far east	
Poa	*hartzii*	subsp.	*vrangelica*		2n = 56	*P. vrangelica* in Probatova 2007, originally *P. tolmatschewii* var. *stricta*	Zhukova & Petrovsky 1971 Zukova & Petrovsky 1972 Petrovsky & Zukova 1981	1		RUSSIA far east	
Poa	*napensis*				2n = 42	*P. napensis*	Soreng 1991a; more info. 2005	1	Soreng-2926	USA, CA	US
Poa	*secunda*		intermediate between subspp.		2n = 56	*P. canbyi*	Bowden 1961	1	Bowden-1197	Canada, AB	DAO!
Poa	*secunda*		intermediate between subspp.		2n = 70	*P. canbyi*	Bowden 1961	1	Bowden-1198 (Dore 12000)	Canada, AB	DAO!
Poa	*secunda*		intermediate between subspp.		2n = 70	*P. canbyi*	Bowden unreported	1	Bowden-1220 (Dore 12023, Jenkins 5437); 1220 reported as 2n≈98 on two other sheats (see above) was apparently on a separate clone, or 2n=70 written on the present two sheats was in error	Canada, AB	DAO!
Poa	*secunda*		intermediate between subspp.		2n = 100	*P. glaucifolia,* but = *P. secunda secunda (porteri* form) towards *juncifolia*	Bowden 1961	1	Bowden-1189	Canada, AB	DAO!
Poa	*secunda*	subsp.	*juncifolia*	*juncifolia*	2n = 42	*P. cusickii* !	Hartung 1946	1	CI 4531-11 (Keck 5408)	USA, CA	CI
Poa	*secunda*	subsp.	*juncifolia*	*ampla*	2n = 56	*P. secunda,* ampla like plants	Kellogg 1983 (dissertation)	1		USA, OR	
Poa	*secunda*	subsp.	*juncifolia*	*juncifolia*	2n = 60	*P. juncifolia*	Bowden 1961	1	Bowden-1192	Canada, AB	DAO!
Poa	*secunda*	subsp.	*juncifolia*	*ampla*	2n = 61	*P. ampla*	Hiesey & Nobs 1982	1	CI 4197	USA, ID	CI
Poa	*secunda*	subsp.	*juncifolia*	*ampla*	2n = 62	*P. confusa*	Armstrong 1937	4	S.E. Clarke	Canada, AB	
Poa	*secunda*	subsp.	*juncifolia*	*nevadensis*	2n = 62	*P. nevadensis*	Armstrong 1937	2	SES	USA	
Poa	*secunda*	subsp.	*juncifolia*	*ampla*	2n = 62	*P. ampla*	Bowden 1961	1	Bowden-1209	Canada, AB	DAO!
Poa	*secunda*	subsp.	*juncifolia*	*ampla*	2n = 62	*P. ampla*	Hartung 1946	1	CI 4177-1	USA, WA	CI
Poa	*secunda*	subsp.	*juncifolia*	*juncifolia*	2n = 62	*P. juncifolia*	Hartung 1946	1	CI 4197-11	USA, ID	CI
Poa	*secunda*	subsp.	*juncifolia*	*nevadensis*	2n = 62	*P. nevadensis*	Hiesey & Nobs 1982	1	CI 4476	USA, CA	CI
Poa	*secunda*	subsp.	*juncifolia*	*nevadensis*	2n = 62-63	*P. nevadensis*	Hartung 1946	1	CI 4190-1	USA, OR	CI
Poa	*secunda*	subsp.	*juncifolia*	*ampla*	2n = 63	*P. ampla*	Hartung 1946	1	CI 4172-1	USA, OR	CI
Poa	*secunda*	subsp.	*juncifolia*	*ampla*	2n = 63	*P. ampla*	Hartung 1946	1	CI 4174-1	USA, WA	CI
Poa	*secunda*	subsp.	*juncifolia*	*ampla*	2n = 63	*P. ampla*	Hartung 1946	2	CI 4175-1,2	USA, WA	CI
Poa	*secunda*	subsp.	*juncifolia*	*ampla*	2n = 63	*P. ampla*	Hartung 1946	1	CI 4177-1	USA, WA	CI
Poa	*secunda*	subsp.	*juncifolia*	*ampla*	2n = 63	*P. ampla*	Hartung 1946	1	CI 4180-13	USA, WA	CI
Poa	*secunda*	subsp.	*juncifolia*	*ampla*	2n = 63	*P. ampla*	Hartung 1946	1	CI 4182-11	USA, WA	CI
Poa	*secunda*	subsp.	*juncifolia*	*ampla*	2n = 63	*P. ampla*	Hartung 1946	1	CI 4184-1	USA, WA	CI
Poa	*secunda*	subsp.	*juncifolia*	*ampla*	2n = 63	*P. ampla*	Hartung 1946	1	CI 4185-11	USA, WA	CI

Poa	*secunda*	subsp.	*juncifolia*	*ampla*	2n = 63	*P. ampla*	Hartung 1946	1	CI 4186-1	USA, OR	CI
Poa	*secunda*	subsp.	*juncifolia*	*ampla*	2n = 63	*P. ampla*	Hartung 1946	1	CI 4188-11	USA, WY	CI
Poa	*secunda*	subsp.	*juncifolia*	*nevadensis*	2n = 63	*P. nevadensis*	Hartung 1946	1	CI 4192-1	USA, OR	CI
Poa	*secunda*	subsp.	*juncifolia*	*ampla*	2n = 63	*P. ampla*	Hartung 1946	1	CI 4193-1	USA, OR	CI
Poa	*secunda*	subsp.	*juncifolia*	*nevadensis*	2n = 63	*P. nevadensis*	Hartung 1946	1	CI 4194-1	USA, ID	CI
Poa	*secunda*	subsp.	*juncifolia*	*nevadensis*	2n = 63	*P. nevadensis*	Hartung 1946	1	CI 4195-1	USA, ID	CI
Poa	*secunda*	subsp.	*juncifolia*	*nevadensis*	2n = 63	*P. nevadensis*	Hartung 1946	1	CI 4198-1	USA, ID	CI
Poa	*secunda*	subsp.	*juncifolia*	*nevadensis*	2n = 63	*P. nevadensis*	Hartung 1946	1	CI 4475-11	USA, CA	CI
Poa	*secunda*	subsp.	*juncifolia*	*nevadensis*	2n = 63	*P. nevadensis*	Hartung 1946	1	CI 4478-2	USA, CA	CI
Poa	*secunda*	subsp.	*juncifolia*	*nevadensis*	2n = 63	*P. nevadensis*	Hartung 1946	1	CI 4534-13	USA, CA	CI
Poa	*secunda*	subsp.	*juncifolia*	*juncifolia*	2n = 63	*P. juncifolia*	Soreng 1991a; more info. 2005	1	Soreng-821	USA, NV	US
Poa	*secunda*	subsp.	*juncifolia*	*ampla*	2n ≈ 63	*P. ampla*	Hartung 1946	1	CI 4173-1	USA, OR	CI
Poa	*secunda*	subsp.	*juncifolia*	*ampla*	2n ≈ 63	*P. ampla*	Hartung 1946	1	CI 4179-1	USA, WA	CI
Poa	*secunda*	subsp.	*juncifolia*	*ampla*	2n ≈ 63	*P. ampla*	Hartung 1946	1	CI 4199-1	USA, ID	CI
Poa	*secunda*	subsp.	*juncifolia*	*juncifolia*	2n = 63-64	*P. juncifolia*	Hartung 1946	1	CI 4181-1	USA, WA	CI
Poa	*secunda*	subsp.	*juncifolia*	*ampla*	2n = 63-64	*P. ampla*	Hiesey & Nobs 1982	1	CI 4181	USA, WA	CI
Poa	*secunda*	subsp.	*juncifolia*	*ampla*	2n = 63-64	*P. ampla*	Hiesey & Nobs 1982	1	CI 4183	USA, WA	CI
Poa	*secunda*	subsp.	*juncifolia*	*ampla*	2n = 64	*P. ampla*	Armstrong 1937	2	SES	USA	
Poa	*secunda*	subsp.	*juncifolia*	*ampla*	2n = 64	*P. ampla*	Hartung 1946	1	CI 4171-1	USA, OR	CI
Poa	*secunda*	subsp.	*juncifolia*	*ampla*	2n = 64	*P. ampla*	Hartung 1946	1	CI 4178-1	USA, WA	CI
Poa	*secunda*	subsp.	*juncifolia*	*ampla*	2n = 64	*P. ampla*	Hartung 1946	1	CI 4183-1	USA, WA	CI
Poa	*secunda*	subsp.	*juncifolia*	*nevadensis*	2n = 64	*P. nevadensis*	Hartung 1946	1	CI 4189-11	USA, OR	CI
Poa	*secunda*	subsp.	*juncifolia*	*ampla*	2n = 64	*P. ampla*	Hartung 1946	1	CI 4473-11	USA, CA	CI
Poa	*secunda*	subsp.	*juncifolia*	*ampla*	2n = 64	*P. ampla*	Hartung 1946	1	CI 4586-11	USA, WY	CI
Poa	*secunda*	subsp.	*juncifolia*	*nevadensis*	2n = 64-66	*P. nevadensis*	Stebbins & Love 1941	1	Stebbins 2918	USA, CA	DAV, UC/JEP
Poa	*secunda*	subsp.	*juncifolia*	*nevadensis*	2n = 64-66	*P. nevadensis*	Stebbins & Love 1941	1	Stebbins 2922	USA, CA	DAV, UC/JEP
Poa	*secunda*	subsp.	*juncifolia*	*nevadensis*	2n ≈ 65	*P. nevadensis*	Hartung 1946	1	CI 4477-3	USA, CA	CI
Poa	*secunda*	subsp.	*juncifolia*	*ampla*	2n ≈ 65	*P. ampla*	Hartung 1946	1	CI 4187-11	USA, ID	CI
Poa	*secunda*	subsp.	*juncifolia*	*nevadensis*	2n ≈ 66	*P. nevadensis*	Hartung 1946	1	CI 4191-1	USA, OR	CI
Poa	*secunda*	subsp.	*juncifolia*	*nevadensis*	2n = 70	*P. nevadensis*	Hartung 1946	1	CI 4200-1	USA, ID	CI
Poa	*secunda*	subsp.	*juncifolia*	*ampla*	2n = 70-71	*P. ampla*	Hartung 1946	1	CI 4196-11	USA, ID	CI
Poa	*secunda*	subsp.	*juncifolia*	*juncifolia*	2n = 78	*P. juncifolia*	Hartung 1946	1	CI 4592-11	USA, WY	CI
Poa	*secunda*	subsp.	*juncifolia*	*juncifolia*	2n = 84	*P. juncifolia*	Hartung 1946	1	CI 4589-11	USA, WY	CI
Poa	*secunda*	subsp.	*juncifolia*	*ampla*	2n ≈ 97	*P. ampla*	Hartung 1946	1	CI 4186-12	USA, OR	CI
Poa	*secunda*	subsp.	*juncifolia*	*ampla*	2n ≈ 100	*P. ampla*	Hartung 1946	1	CI 4183-13	USA, WA	CI
Poa	*secunda*	subsp.	*secunda*	*secunda*	2n = 42	*P. secunda*	Bowden 1961	1	Bowden-1188	Canada, AB	DAO!
Poa	*secunda*	subsp.	*secunda*	*scabrella*	2n = 44+f	*P. scabrella*	Hartung 1946	1	CI 4495-1	USA, CA	CI
Poa	*secunda*	subsp.	*secunda*	*gracillima* or *secunda*	2n ≈ 48	*P. secunda*, open panicled plants	Kellogg 1983	1		USA, ID	
Poa	*secunda*	subsp.	*secunda*	*secunda*	2n = 56	*P. secunda*	Bowden 1961	1	Bowden-1199	Canada, AB	DAO!
Poa	*secunda*	subsp.	*secunda*	*scabrella*	2n = 61-63	*P. scabrella*	Hartung 1946	1	CI 4510-11	USA, CA	CI
Poa	*secunda*	subsp.	*secunda*	*scabrella*	2n ≈ 62	*P. scabrella*	Hartung 1946	1	CI 4214-4	USA, CA	CI
Poa	*secunda*	subsp.	*secunda*	*scabrella*	2n = 63	*P. scabrella*	Hartung 1946	2	CI 4217-17,18	USA, CA	CI
Poa	*secunda*	subsp.	*secunda*	*scabrella*	2n = 63	*P. scabrella*	Hartung 1946	1	CI 4219-11	USA, CA	CI
Poa	*secunda*	subsp.	*secunda*	*scabrella*	2n = 63	*P. scabrella*	Hartung 1946	1	CI 4223-13	USA, CA	CI
Poa	*secunda*	subsp.	*secunda*	*scabrella*	2n = 63	*P. scabrella*	Stebbins & Love 1941	1	Stebbins 2698	USA, CA	DAV, UC/JEP
Poa	*secunda*	subsp.	*secunda*	*scabrella*	2n = 64	*P. scabrella*	Hartung 1946	1	CI 4217-16	USA, CA	CI
Poa	*secunda*	subsp.	*secunda*	*scabrella*	2n ≈ 66	*P. scabrella*	Stebbins & Love 1941	1	Stebbins 2711	USA, CA	DAV, UC/JEP
Poa	*secunda*	subsp.	*secunda*	*secunda*	2n ≈ 68	*P. secunda*	Kellogg 1983	1		Canada, QU	
Poa	*secunda*	subsp.	*secunda*	*scabrella*	2n ≈ 68	*P. scabrella*	Hiesey & Nobs 1982	1	CI 4217	USA, CA	CI
Poa	*secunda*	subsp.	*secunda*	*secunda*	2n = 70	*P. secunda*	Bowden 1961	1	Bowden-1203	Canada, AB	DAO!
Poa	*secunda*	subsp.	*secunda*	*secunda*	2n = 70	*P. canbyi*	Bowden 1961	1	Bowden-1204	Canada, AB	DAO!
Poa	*secunda*	subsp.	*secunda*	*secunda*	2n = 70	*P. canbyi*	Bowden 1961	1	Bowden-1206	Canada, AB	DAO!
Poa	*secunda*	subsp.	*secunda*	*secunda*	2n = 70	*P. secunda*	Kellogg 1983	1		Canada, QU	

Poa	*secunda*	subsp.	*secunda*	*secunda*	2n ≈ 70	*P. secunda*	Bowden 1961	1	Bowden-1207	Canada, AB	DAO!
Poa	*secunda*	subsp.	*secunda*	*secunda*	2n ≈ 70	*P. secunda*	Bowden 1961	1	Bowden-1208	Canada, AB	DAO!
Poa	*secunda*	subsp.	*secunda*	*secunda*	2n ≈ 72	*P. canbyi*	Hartung 1946	1	CI 4590-11	USA, WY	CI
Poa	*secunda*	subsp.	*secunda*	*secunda*	2n ≈ 74	*P. secunda*	Hartung 1946	1	CI 4239-1	USA, OR	CI
Poa	*secunda*	subsp.	*secunda*	*secunda*	2n ≈ 78	*P. secunda*	Bowden 1961	1	Bowden-1184	Canada, AB	DAO!
Poa	*secunda*	subsp.	*secunda*	*secunda*	2n ≈ 78	*P. secunda*	Bowden 1961	1	Bowden-1211	Canada, AB	DAO!
Poa	*secunda*	subsp.	*secunda*	*secunda*	2n = 80	*P. secunda*	Bowden 1961	1	Bowden-1173	Canada, AB	DAO!
Poa	*secunda*	subsp.	*secunda*	*scabrella*	2n = 81	*P. scabrella*	Hartung 1946	1	CI 4223-14	USA, CA	CI
Poa	*secunda*	subsp.	*secunda*	*gracillima*	2n = 81	*P. gracillima*	Hartung 1946	3	CI 4226-1,3,6	USA, CA	CI
Poa	*secunda*	subsp.	*secunda*	*secunda*	2n = 81	*P. secunda*	Hartung 1946	1	CI 4236-1	USA, ID	CI
Poa	*secunda*	subsp.	*secunda*	*scabrella*	2n = 81	*P. scabrella*	Hartung 1946	1	CI 4484-11	USA, CA	CI
Poa	*secunda*	subsp.	*secunda*	*gracillima*	2n ≈ 81	*P. gracillima*	Hartung 1946	1	CI 4226-2	USA, CA	CI
Poa	*secunda*	subsp.	*secunda*	*scabrella*	2n = 82	*P. scabrella*	Hartung 1946	1	CI 4212-4	USA, CA	CI
Poa	*secunda*	subsp.	*secunda*	*scabrella*	2n = 82	*P. scabrella*	Hartung 1946	1	CI 4214-1	USA, CA	CI
Poa	*secunda*	subsp.	*secunda*	*secunda*	2n = 82	*P. secunda*	Stebbins & Love 1941	1	Stebbins 2718	USA, CA	DAV, UC/JEP
Poa	*secunda*	subsp.	*secunda*	*secunda*	2n = 82	*P. secunda*	Stebbins & Love 1941	1	Stebbins 2785	USA, CA	DAV, UC/JEP
Poa	*secunda*	subsp.	*secunda*	*scabrella*	2n = 82 (see 4213-11 of Hartung)	*P. scabrella*	Hiesey & Nobs 1982	1	CI 4213	USA, CA	CI
Poa	*secunda*	subsp.	*secunda*	*secunda*	2n ≈ 82	*P. canbyi*	Hartung 1946	1	CI 4205-1	USA, WA	CI
Poa	*secunda*	subsp.	*secunda*	*secunda*	2n ≈ 82	*P. canbyi*	Hartung 1946	1	CI 4209-1	USA, ID	CI
Poa	*secunda*	subsp.	*secunda*	*secunda*	2n ≈ 83	*P. canbyi*	Hartung 1946	1	CI 4210-1	USA, ID	CI
Poa	*secunda*	subsp.	*secunda*	*scabrella*	2n = 84	*P. scabrella*	Armstrong 1937	2	SES	USA	
Poa	*secunda*	subsp.	*secunda*	*secunda*	2n = 84	*P. canbyi*	Hartung 1946	1	CI 4206-1	USA, WA	CI
Poa	*secunda*	subsp.	*secunda*	*secunda*	2n = 84	*P. canbyi*	Hartung 1946	1	CI 4207-1	USA, WA	CI
Poa	*secunda*	subsp.	*secunda*	*secunda*	2n = 84	*P. canbyi*	Hartung 1946	1	CI 4211-1	USA, MT	CI
Poa	*secunda*	subsp.	*secunda*	*scabrella*	2n = 84	*P. scabrella*	Hartung 1946	1	CI 4215-11	USA, CA	CI
Poa	*secunda*	subsp.	*secunda*	*scabrella*	2n = 84	*P. scabrella*	Hartung 1946	1	CI 4221-11	USA, CA	CI
Poa	*secunda*	subsp.	*secunda*	*scabrella*	2n = 84	*P. scabrella*	Hartung 1946	1	CI 4222-2	USA, CA	CI
Poa	*secunda*	subsp.	*secunda*	*scabrella*	2n = 84	*P. scabrella*	Hartung 1946	3	CI 4223-11,12,15	USA, CA	CI
Poa	*secunda*	subsp.	*secunda*	*scabrella*	2n = 84	*P. scabrella*	Hartung 1946	1	CI 4228-11	USA, CA	CI
Poa	*secunda*	subsp.	*secunda*	*scabrella*	2n = 84	*P. scabrella*	Hartung 1946	1	CI 4229-11	USA, CA	CI
Poa	*secunda*	subsp.	*secunda*	*secunda*	2n = 84	*P. canbyi*	Hartung 1946	1	CI 4233-1	USA, CA	CI
Poa	*secunda*	subsp.	*secunda*	*secunda*	2n = 84	*P. secunda*	Hartung 1946	1	CI 4235-1 (4334 by H&N)	USA, ID	CI
Poa	*secunda*	subsp.	*secunda*	*scabrella*	2n = 84	*P. scabrella*	Hartung 1946	1	CI 4490-11	USA, CA	CI
Poa	*secunda*	subsp.	*secunda*	*secunda*	2n = 84	*P. secunda*	Hiesey & Nobs 1982	1	CI 4236 (see Hartung count)	USA, ID	CI
Poa	*secunda*	subsp.	*secunda*	*scabrella*	2n = 84	*P. scabrella*	Hiesey & Nobs 1982	1	CI 4490	USA, CA	CI
Poa	*secunda*	subsp.	*secunda*	*scabrella*	2n = 84	*P. scabrella*	Stebbins & Love 1941	1	Stebbins 2898	USA, CA	DAV, UC/JEP
Poa	*secunda*	subsp.	*secunda*	*gracillima*	2n ≈ 84	*P. gracillima*	Hartung 1946	2	CI 4226-4,5	USA, CA	CI
Poa	*secunda*	subsp.	*secunda*	*scabrella*	2n ≈ 84	*P. scabrella*	Hartung 1946	1	CI 4511-11	USA, CA	CI
Poa	*secunda*	subsp.	*secunda*	*secunda*	2n ≈ 84	*P. canbyi*	Hartung 1946	1	CI 4595-11	Canada, AB	CI
Poa	*secunda*	subsp.	*secunda*	*secunda*	2n ≈ 84	*P. canbyi*	Hiesey & Nobs 1982	1	CI 4201	USA, CA	CI
Poa	*secunda*	subsp.	*secunda*	*scabrella*	2n = 84+f	*P. scabrella*	Hartung 1946	1	CI 4212-7	USA, CA	CI
Poa	*secunda*	subsp.	*secunda*	*secunda*	2n = 84-88+II	*P. secunda*	Soreng 1991a; more info. 2005	1	Soreng-1135	USA, MT	US
Poa	*secunda*	subsp.	*secunda*	*secunda*	2n ≈ 84	*P. secunda*	Stebbins & Love 1941	1	Stebbins 2853	USA, CA	DAV, UC/JEP
Poa	*secunda*	subsp.	*secunda*	*secunda*	2n = 85	*P. canbyi*	Hartung 1946	1	CI 4230-1	USA, OR	CI
Poa	*secunda*	subsp.	*secunda*	*secunda*	2n = 85-87	*P. secunda*	Hartung 1946	1	CI 4237-1	USA, MT	CI
Poa	*secunda*	subsp.	*secunda*	*secunda*	2n = 86	*P. secunda*	Hartung 1946	1	CI 4231-11	USA, OR	CI
Poa	*secunda*	subsp.	*secunda*	*gracillima*	2n = 86	*P. gracillima*	Hartung 1946	1	CI 4240-13	USA, CA	CI
Poa	*secunda*	subsp.	*secunda*	*secunda*	2n = 86	*P. secunda*	Stebbins & Love 1941	1	Stebbins 2712	USA, CA	DAV, UC/JEP
Poa	*secunda*	subsp.	*secunda*	*scabrella*	2n = 86	*P. scabrella*	Hartung 1946	1	CI 4213-11	USA, CA	CI
Poa	*secunda*	subsp.	*secunda*	*secunda*	2n ≈ 86	*P. canbyi*	Hiesey & Nobs 1982	1	CI 4204	USA, CA	CI
Poa	*secunda*	subsp.	*secunda*	*scabrella*	2n ≈ 86	*P. scabrella*	Stebbins & Love 1941	1	Stebbins 2699	USA, CA	DAV, UC/JEP
Poa	*secunda*	subsp.	*secunda*	*scabrella*	2n ≈ 86	*P. scabrella*	Stebbins & Love 1941	1	Stebbins 2787	USA, CA	DAV, UC/JEP

Poa	*secunda*	subsp.	*secunda*	*secunda*	2n ≈ 87	*P. secunda*	Hartung 1946	1	CI 4234-1 (4334 by H&N)	USA, ID	CI
Poa	*secunda*	subsp.	*secunda*	*scabrella*	2n ≈ 88	*P. scabrella*	Hartung 1946	1	CI 4509-11	USA, CA	CI
Poa	*secunda*	subsp.	*secunda*	*secunda*	2n = 90	*P. canbyi*	Hartung 1946	1	CI 4225-3	USA, CA	CI
Poa	*secunda*	subsp.	*secunda*	*scabrella*	2n ≈ 91	*P. scabrella*	Hartung 1946	1	CI 4223-17	USA, CA	CI
Poa	*secunda*	subsp.	*secunda*	*gracillima*	2n ≈ 91	*P. gracillima*	Hiesey & Nobs 1982	1	CI 4227	USA, CA	CI
Poa	*secunda*	subsp.	*secunda*	*secunda*	2n = 93	*P. canbyi*	Hartung 1946	1	CI 4518-11	USA, CA	CI
Poa	*secunda*	subsp.	*secunda*	*secunda*	2n ≈ 94	*P. canbyi*	Hartung 1946	1	CI 4224-4	USA, CA	CI
Poa	*secunda*	subsp.	*secunda*	*secunda*	2n ≈ 98	*P. canbyi*	Bowden 1961	1	Bowden-1220 (Dore 12023)	Canada, AB	DAO!
Poa	*secunda*	subsp.	*secunda*	*secunda*	2n ≈ 99	*P. secunda*	Hiesey & Nobs 1982	1	CI 4225	USA, CA	CI
Poa	*secunda*	subsp.	*secunda*	*secunda*	2n ≈ 99	*P. canbyi*	Hartung 1946	1	CI 4225-1	USA, CA	CI
Poa	*secunda*	subsp.	*secunda*	*scabrella* or *secunda*	2n = 104	*P. scabrella* , (*P. secunda* in Hiesey &Nobs, 1982)	Hartung 1946	1	CI 4530-11	USA, CA	CI
Poa	*secunda*	subsp.	*secunda*	*secunda*	2n = 105-106	*P. canbyi*	Hartung 1946	1	CI 4225-5	USA, CA	CI
Poa	*stenantha*	var.	*stenantha*		2n = 42	*P. stenantha*	Taylor & Mulligan 1968	1	Calder & Taylor 36417	Canada, BC, QC Isls.	DAO!
Poa	*stenantha*				2n = 81*	*P. stenantha*	Hartung 1946 (?)				
Poa	*stenantha*				2n = 84	*P. stenantha*	Taylor & Mulligan 1968	1		Canada	
Poa	*stenantha*	var.	*stenantha*		2n = 84*	*P. stenantha*	Hartung 1946 (?)				
Poa	*stenantha*				2n = 86*	*P. stenantha*	Hartung 1946 (?)				
Poa	*tenerrima*				2n = 42	*P. tenerrima*	Stebbins In Munz 1959	1		USA, CA	
Poa	*unilateralis*	subsp.	*pachypholis*		2n = 42	*P. pachypholis*	Spellenberg 1970; Soreng 2005 more info	1	Spellenberg & Southerland 1522a	USA, WA	NMC!
Poa	*unilateralis*	subsp.	*unilateralis*		2n = 42	*P. unilateralis*	Soreng 1991a (Stebbins ref. to (Myers 1947))	1	Stebbins	USA, CA	
Poa	*unilateralis*	subsp.	*unilateralis*		2n = 84	*P. unilateralis*	Soreng 1991a; more info. 2005	1	Clausen 2151	USA, OR	
Poa	*glauca*					*P. stenantha* , but = *P. glauca*	Bowden 1961	*1*	Bowden-921	Canada, DM	DAO!
Poa	*pratensis*					*P. secunda* , but = *P. pratensis*	Bowden 1961	*1*	Bowden-1195	Canada, AB	DAO!

Armstrong JM (1937) A cytological study of the genus *Poa.* Canad. J. Res. 15: 281–297.

Bowden WM (1961) Chromosome numbers and taxonomic notes on northern grasses. IV. Tribe Festuceae: *Poa* and *Puccinellia.* Canad. J. Bot. 39: 123–138.

Hartung ME (1946) Chromosome numbers in *Poa, Agropyron* and *Elymus.* Amer. J. Bot. 33(6): 516–531.

Hiesey WM, Nobs MA (1982) Experimental studies on the nature of species VI. Interspecific hybrid derivatives between facultatively apomictic species of bluegrasses and their responses to contrasting environments. Publ. Carnegie Inst. Wash. 636: i–x, 1–199.

Holmen K (1952) Cytological studies in the Flora of Peary Land, North Greenland. Medd. Groenl. 128: 1-40

Jorgensen CA, Sorensen T, Westergaard M (1958) The flowering plants of Greenland. A taxonomical and cytological survey. Biol. Skr. 9(4): 1–172.

Kellogg EA (1983) A biosystematics study of the *Poa secunda* complex. Harvard University, Dissertation, Cambridge.

Löve A, Löve D (1975) In IOPB chromosome number reports L. Taxon 24: 671–678.

Löve A, Löve D (1975) In IOPB chromosome number reports XLIX. Taxon 24: 501–516.

Löve A, Löve D (1975) In IOPB chromosome number reports XLVIII. Taxon 24: 367–372.

Löve A, Löve D (1975) Nomenclatural notes on arctic plants. Bot. Not. 128: 495–523.

Myers WM (1947) Cytology and genetics of forage grasses. Bot. Rev. (Lancaster) 13: 319–422.

Petrovsky VV, Zhukova PG (1981) Chromosome numbers and taxonomy of some plant species of Wrangel Island. Bot. Zhurn. 66 (3): 380–387.

Petrovsky VV, Zhukova PG (1981) Chromosome numbers and taxonomy of some plant species of Wrangel Island. Bot. Zhurn. 66 (3): 380–387.

Soreng RJ (1991) Notes on new infraspecific taxa and hybrids in North American *Poa* (Poaceae). Phytologia 71: 390–413.

Soreng RJ (2005) Miscellaneous chromosome number reports for *Poa* (Poaceae) in North America. Sida 21(4): 2195–2203.

Spellenberg RW (1970) IOPB chromosome number reports XXV. Taxon 19(1): 112–113

Stebbins GL In: Munz PA (1959) A California Flora. 1–1681. University of California Press, Berkeley.

Stebbins GL, Love RM (1941) A cytological study of California forage grasses. Amer. J. Bot. 28: 371–382.

Taylor RL, Mulligan GA (1968) Flora of the Queen Charlotte Islands. Part 2. Cytological Aspects of the Vascular Plants.

Zhukova PG, Petrovsky VV (1971) Chromosome numbers of some flowering plants of Wrangel Island. Bot. Zhurn. 56(2): 294-305.

Zhukova PG, Petrovsky VV (1972) Chromosome numbers of some flowering plants of Wrangel Island. II . Bot. Zhurn. 57(4): 554-563.

Zhukova PG, Petrovsky VV (1976) Chromosome numbers of some Western Chukotka plant species, II. Bot. Zhurn. 61(7): 963–969.
