# Supplementary material for: Poasecunda J. Presl (Poaceae): a modern summary of infraspecific taxonomy, chromosome numbers, related species and infrageneric placement based on DNA
Source: PhytoKeys. 2018 Nov 5;(110):101–21. doi: 10.3897/phytokeys.110.27750 (PMC6232245; doi:10.3897/phytokeys.110.27750)
Supplement: Supplementary material 2 — David D. Keck’s annotations of taxa here included in Poasecunda [file phytokeys-110-101-s002.pdf]

Poa ampla Merr.

ALASKA: Skagway, single bunch in old abandoned garden spot, 7/11/09, Hitchcock 4206 (US

[SASKATCHEWAN: Cypress Hills, Assiniboia, 6/27/95, Macoun 13302 (US

Farwell Ck, s of Cypress Hills, 6/27/95, J Macoun 13302 (NY = ? Porteri !

ALBERTA: [Pot Hole Ck, S of Lethbridge, 7/21/95, J Macoun 13308 (NY = Porteri]

[White Mud R, 6/22/95, J Macoun 13300 (NY ! 6/21/95, J Macoun 13301 (NY ampla ligule but puberulent lemmas of Canbyi! lvs. short and ± firm but wide for juncifd.! ] = juncifolia ssp. Porteri

Milk R Ridge, 7/18/95, J Macoun 13298 (NY

Maple Creek, 7/2/81, J Macoun 20 (NY !

Slope of Squaw Mt, nr. Banff, 5200', 8/17/99, McCalla 2321a (NY

Craigmyle District, grassland in garden, 7/4/35, Brinkman 4772a (US

Crow Nest Pass, 7/30/97, J Macoun 33982 (NY
Waterton Lakes N.P.; 4100', Breitung

BRITISH COLUMBIA

Kootenay: [gravelly soil on moraine in valley of Elk R, along Goat Ck, 27 mi N of Natal, 7/4/41, Weber 2293 (C, NY, US, UW] = juncifolia ssp. Porteri

Deer Park, Lower Arrow Lake, 6/5/90, Macoun 11119 (US do, 6/18/90, Macoun 97 (US

Trail, 6/6/02, Macoun 63434 (NY, US

Cascade, 6/26/02, Macoun 63432 (CAS, NY, US), 63433 (US , 6/28/02, 63443 (NY
Kootenay L, JKHenry 555 (RM

Yale: Penticton, very dry bench land, 6/5/38, Eastham 44 (US

Okanagan R. opp. Granite Creek, 6/21/18, Hutchinson 2 (US

Nanaimo (?): Sea coast, Gordon Head, Vancouver Island, 5/30/87, Macoun 11 (US good ampla but lig. up to 3.6 mm long and lemmas almost crisp-puberulent on nerves

Gordon Head, 5/30/87, Macoun 25 (NY good ampla!

NORTH DAKOTA

Billings: sandy clay slope, edge of alkaline draw, 1.5 mi from Sully Sprgs, 8/1/39, Swallen 5818 (US 5816 (US

Tracy Mtn, 7/28/39, Swallen 5792 (US

alkali flat E of Golva, 6/8/36, GL Weber N9-64 (US

SOUTH DAKOTA

Custer: Custer, Black Hills, 6/24-28/14, Hitchcock 11133 (US

do, do, pine woods, rocky hills, 11129 (US

[Meade: Elk Canyon, Black Hills, 4-5000', 6/29/92, Rydberg 1146 (NY] = juncif. Porteri

NEBRASKA

Brown: Long Pine, 5/30/97, JM Bates 1094 (NY !

NEBRASKA, cont.

Co.?: Low ground at mouth of War Bonnet Canyon, 4200', 6/24/90, Williams (NY

MONTANA

Lake: Wild Horse Island, Flathead Lake, 8/12/08, Clemens (SU

do, do, 3000', 8/13/08, Jones 9556 (SU

Lewis and Clark: Helena, 7/7/91, Kelsey (NY !

Meagher: [Elk Mts nr Castle, 6000', 8/1/96, Flodman 151 (NY = juncif. Porteri]
Little Elk Mts, nr. Black Hawk, 8/5/96, Rydberg 3263 (NY , [Black Hawk, mdw, 8/1/96, Rydberg 3272 (NY ~~toward~~= juncifol. Porteri ]
[Castle, 8/1/96, Rydb. 3243 (NY = " " ]
Fergus: Half Moon Canyon, Big Snowy Mts., 7/5/45, Hitch. + Muhlick 11987 (UW lemmas ± pubescent like Canbyi, but big; lvs. wide; ligule blunt; good ampla
Cascade: Great Falls, 6/87, FW Anderson 9414 (NY
Missoula: 1 mi. E. of Missoula, 3200', 5/28/36, Hitchcock 2946 (UW
Madison (or Gallatin?): Spanish Basin, Madison Range, 6000', 7/17/96, Flodman 147 (NY ligule rather long but blunt
Round Hill Vig. Exp. Range, 8600', 7/25/41, Lemon & Hurtt 144 (Minn
do, 7/11/96, Flodman 144 (NY

Spanish Peaks, Madison Range, 7-8000', 7/14/96, Flodman 146 (NY! good ampla!
Spanish Creek, 7/11/96, Rydberg 3050 (NY
Spanish Basin, 7/20/96, Rydb. 3161 (NY ! short lig! 7/19/96, Rydberg 3161 (NY toward juncifolia.

Spanish Basin, 7/17/96, Rydb. 3111 (NY

do, 7/19/96, Rydb. 3166 (NY toward juncifolia
Gravelly Range, 6 mi. NE. of Crockett Lake, 8000', 7/19/45, Hitch. + Muhlick 12483 (UW
Jack Creek Canyon, 7000', 7/14/97, Rydberg & Bessey 3665 (NY
Powell: Flathead R., 1 mi. N.W. of Big Prairie R.S., 7/20/48, Hitchcock 18578 (UW , 18605 (UW
Gallatin: Bozeman, 7/25/95, Rydb. 2259 (NY
Powell: 20 mi. W. of Lincoln on Ovando Rd., 6/26/45, Hitch. + Muhlick 11558 (UW
[illegible] Bridger Mts nr the Pass, 8000', 7/28/96, Flodman 148 (NY excellent ampla except lemmas slightly pubescent on the sides)
Beaverhead: wet mdw. ca. 15 mi. W. of Wisdom on Gibbonsville Pass rd, 6/29/44, Hitch. + Muhlick 1207 (UW
Co.?: Jack Creek Canyon (nr. YNP?), 7000', 7/14/97, Rydb. and Bessey 3665 (NY
prairie ground bordering L. Bowdoin, 7 mi. E. of Malta, 6/10/35, Marsh 743 (UW

WYOMING

**Y.N.P.; Moose Falls, open ground, 8/21/99, A & E Nelson 6732 (RM** @ NY = Canbyi
Yellowstone Lake, 1885, Tweedy 641 (GH labelled "Poa duriuscula Scribn. (n.sp.)"
" " **, open ~~partly wooded~~ slopes, 8/23/99, A & ENelson 6630 (RM**
Sheridan: Tongue R, 8/16/92, Buffum (C, RM

**foothills betw. Sheridan and Buffalo, 6000', 6/15-7/15/00, Tweedy 3698 in pt(NY**
one sheet of Canbyi under this no. at NY do 3699 (RM, NY
**Lower Tongue C&H Allot, Bighorn NF, 8500', 9/15/51, Hurd 257 (RM invol. lvs.** but big ampla type
**N. Johnson and W. Sheridan: Clear Creek, 8/98, Griffiths 134 (C**

Johnson: E slope Big Horn Mts, hdwaters Clear Ck and Crazy Woman R, 7-9000', 1900, Tweedy 3696 (NY, RM
washes, Middle Fork Powder R, 7/16/01, Goodding 269 (RM
Buffalo, 4-5000', 7/00, Tweedy 3694 (NY
Converse: badlands, T38, R68, 4600', 7/7/36, Ownbey + Long 1063 (RM
Natrona: Bates Ck, 7/5/01, Goodding 200 (NY, RM

Big Horn: Canyon Creek, 7/27/01, Goodding 397 (C, NY, RM, SU meadow
Sublette: Green R. Lakes, 8000', 7/19/40, Porter 5111 (RM [illegible]

WYOMING, cont. **Fremont: 20 mi. W. Dubois, on US 287, 7/21/46, Porter 4055 (RM**

Albany: Pole Mt region, 8400', dry gravelly soil, 7/6/43, Porter 3249, in part (C, CI = Canbyi at SU 8300', bunchgrass in aspen woods, 7/15/46, Porter 4017 (RM.
**Green Top (Mt., 8100'), 6/29/97, ANelson 3257 (RM**
Antelope Basin, 6/9/00, A. Nels. 7479 (Minn, NY, RM, 7508 (RM, NY

Laramie Hills, among sagebrush in deep draws, 7/17/01, E. Nelson 404 (SU, RM. (do., 7/19/01, E.N. 421 (RM

do, 7/19/01, E. Nelson 421 (C toward juncifolia
**Centennial, 7/2/97, A.Nelson 3273 (RM**
Medicine Bow Mts, 9000', dry gravelly soil, 8/2/32, Porter 3308 (C, CI, NY, RM

do, in open parks, 7/28/00, A. Nelson 7787 (Minn (big ampla), NY type of P. confusa Rydg., RM

gravelly roadside nr. University Science Camp, Medicine Bow Mts, 9500', 9/10/44, Porter 3528 (CI
Park: Clay Butte, nr. Beartooth Butte, 9000', 8/18/51, Porter 5923 (RM
Teton: Menoe's Ferry, Jackson's Hole, 7/23/01, Merrill and Wilcon 313 (NY, RM 318 (R

Jackson, along Snake R, 7/13/01, Merrill and Wilcox 262 (SU = nevadensis @ RM

Gros Ventre Ck, 8000', 8/97, Tweedy 21 (NY do, 7500', do, T 22 (NY

upper Hoback Basin, 7500', 8/1-8/00, Curtis (NY

Snake and Lower Hoback rivers, 6500', 8/18-28/00, Curtis (NY
**Sweetwater: 40 mi. S. of Rock Springs, 6500', 6/15/48, Porter 4576 (RM** short form
Lincoln: Sheep Mtn, 7/3/97, A. Nels. 3301 (NY
**Sweetwater: G Summer Ranch, 7/23/97, ANelson 3823 (RM** toward juncifol.

COLORADO:
**Larimer: Grassplot 1, Fort Collins, 6/13/92, StateAgricCollColo 3755 (NY**
Larimer: gulch W of Soldier Can. 6500', 8/8/98, St.Agri. Coll. Colo 3750 (NY, RM
**Moraine Park, 7/11/17, Osterhout 5637 (RM**
**Larimer: Estes Park, 7-9000', July 1884, J. Ball (US**

Routt: Steamboat Springs, July 1891, Eastwood 9 (US 7/20/30, Wooton (US
" " 7/11/13, Osterhout 4961 (RM

Summit: Dillon, 8500', 8/26/96, FE Clements 373 (NY, type of P. truncata Rydg.

Moffat: in sagebrush, w. rim of Lodore Canyon, 7500', 7/9/45, Porter 3671 (C, 3670 (RM, RM
**Boulder: Eldora, 7/28/06, WWRobbins 2384 (RM**
Eagle: McCoy's, sandy moist shady place along Rock Ck, 6500', 7/30/98, Shear & Bessey 1326 (US
**Walcott, 6/21/00, Osterhout 2106 (RM**
Clear Creek: Clear Lake, Georgetown, 8/17/95, Rydberg 2376 (NY lig. less than 1 mm long, but lemma puber.; lvs. short, glaucous, flat = juncifolia Porteri

Co.?: North Park, dry plains, 8/9/99, St. Agric. Coll. Colo. 3754 (NY

IDAHO
**Teton: Victor, dry soil, 7/10/01, Merrill & Wilcox 171 (RM**
Fremont: St. Anthony, 8/15/00, Merrill 64 (SU

Cassia: Shoshone RS, 7/30/38, Gierisch 795 (NY

Oneida: Sublette Canyon, Sec 30, T13S, R31E, nr Minidoka, 6/12/40, Hull 210 (NY

Latah: mdws nr. Viola, 6/20/92, Sandberg 505 (CAS, NY, RM

Clark: Beaver Ck, 3 mi above Spencer, 6000', 6/24/39, Cronquist 1294 in pt, (NY

IDAHO, cont.

Kootenay: nr. Thompson Mt, region of Coeur d'Alene Mts, 630 m, 8/28/95, Leiberg 1614 (F, RM, NY

Nez Perces: Clearwater R, 5/30/92, Sandberg, Heller, McDougal 266 (C, CAS, NY

Big Potlatch R, 6/2/92, Sandberg, Heller, McDougal 305, (CAS, NY, SU
do, 375, (CAS, NY
do, 354, (CAS

nr. Lewiston, 705-1500', 5/11/96, Heller 3042 (NY, SU, RM

Elmore: Nursery Plot 601 Arrowrock substation, 4500', in sagebrush, 7/22/42, Hull 328 (NY

Owyhee: House Creek, 6/29/12, Nelson & Macbride 1788 (NY, RM

De Lamar, 7/7/92, Isabel Mulford 57 (NY

Washington: Rush Creek, 4000', 7/10/99, Jones 6610 (CAS

~~Kootenai: nr Thompson Mt, 630 m, 8/28/95, Leiberg 1614 (NY~~

UTAH Uintah: Little Brush Ck, Ashley Forest, grassy flat nr. stream, [illegible] 8100', 6/19/34, Harrison & Larsen 7828 (BYU rhizome 18 mm.long det. as glaucifolia by Swallen

Salt Lake: City Creek Canyon, 7/25/07, Garrett 2154 (US

alkaline and sandy soil, Beck's Hot Sprgs, NW of Salt Lake City, 6/12/05, Rydb. 6191 (NY, RM = Canby? 6183, RM 6190 (NY

Davis: rd. to Lime Canyon, 4700', 6/10/42, Hurd 172 (NY !

Summit: Blacks Fork, N slope Uinta Mts, T3N, R12E, S33, rocky slopes in scattered lodgepole and aspen, 9000', 9/2/45, B.F. and K.Harrison 10944 (C, US

**Utah: R.S., Payson Canyon, 7800', Liddle 6813 (BYU typical**

Co.?: Whitney RS, Wasatch NF, 9000', 7/13/26, Hitchcock 23083 (CAS, US

NEVADA

Elko: NW Island Lake, Ruby Range, 10800', 8/18/43, Maguire & Holmgren 22649 (NY

Ruby Valley at cave nr Cave Creek PO, 6100', 8/21/08, Heller 9538 (CAS
**Little Lakes Canyon nr. Stampede, 7/21/02, Kennedy 593 (RM**
Jarbidge, 7000', 7/9/12, Nels. & Macbr. 2000 (Minn, NY, RM
**do., do., Upper Corral Creek, 7/12/12, Nelson & Macbride 2032 (RM** 2026 (RM
nr. Gold Ck. on Martin Ck, 8/8/13, Kennedy 4446 (CAS, CI

vic. Bull Camp, E slopes Elk Mtn, Humb. NF, 6/11/39, Maguire 16774 (NY

**Humboldt: N. end Santa Rosa Range, S20, T46N, R40E, 6/11/46, Bunn 1600 (RM**

Lander: nr. Austin, 7/22-25/13, Kennedy 4019 (SU

**dry hills and valleys among sagebrush, Carter's Ranch to Kingston RS, vic. of Austin, Big Ck Canyon, 7/28/13, Hitchcock 10648 (US**

Birch Creek, 6/27/31, JM & MAR Linsdale 468 (CAS

Nye: Stargo Sp(ring), Toiyabe Forest, dry sites, 7800', 6/23/39, Basil K. Crane 315 BC (NY

Douglas: Glenbrook, Lake Tahoe, 6-8000', 8/8/08, Hitchcock 3177 (US, typical
Do., do., 3178 (US definitely toward juncifolia, but with 3177 in "dry sand,

NEVADA, cont.

Mineral: Agnes D. Mine Canyon, N slope Mt. Grant, 8800', 7/5/40, Train 4253 (NY

WASHINGTON

Co.?: Sandberg and Leiberg 531 in 1893 (NY (Peshastin, Chelan Co.)

Stevens: Columbia R 2 mi SW of Northport, 7/8/39, Boner & Weldert 209(C,CAS,NY, RM,SU,UW

Columbia R nr. Gerome, 1290', 5/23/40, Rogers 512 (NY,US,UW

Ferry: Inchelium, sandy W shore of Col.R, 1290',5/25/40,Rogers 531(CAS,Minn,NY, SU,UW

Spokane: N edge Peone Prairie, 5/30/33, Sprague (SU

Sprague, 6/93, Sandberg & Leiberg (NY

Whitman: summit Kamiak Butte, N of Pullman, 6/13/43, Keck and Hiesey 5346(C,CI, SU

bet. Albion and Wilcox, 10 mi NW of Pullman, 6/13/43, Keck and Hiesey 5344 (CI,SU

Endicott, 6/98, Elmer 1025 (Minn

Wawawai, 6/13/96, Piper 2567 (NY

Albion, 8 mi NW Pullman, deep soil, 6/13/43, Keck & Hiesey 5342 (CI

Pullman, 6/23/02, Piper 1837 (C,CAS,F,NY,RM 6/20/93, Piper 1755 (NY,RM

Garfield: Pomeroy, 5/24/44, Hitchcock & Muhlick 8249 (NY,UW 8241 (UW

Okanogan: Oroville, 6/24/11, Jones (SU 5340, 5341 (CI

dry ground on shore of Omak Lake, 7/2/33, Fiker 1237 (NY

Bernhard trail to Mt. Tiffany, 5500', 7/17/32, Fiker 1044 (SU

Grant: border of alkaline pond, Grand Coulee 7 mi above Dry Falls, 6/2/40, Rogers 589 (CAS,Minn,NY,SU,US,UW

Coulee City, 6/1/02, Piper 3916 (US

Hartline, 580 m, 7/2-3/16, Eggleston 12881 (US

Walla Walla: Waitsburg, 6/5/97, Horner 241 (C

Walla Walla, 6/14/00, Williams 3087 (F, SU

Chelan: Chumstick Lookout, alpine slopes, 6000', 7/10/40, Thompson 14979 (CAS,Minn,NY,SU,UW

nr. Entiat, rocky sagebrush slopes, 5/16/31, Thompson 6375 (SU

Peshastin, 1893, Sandberg & Leiberg 531 (NY

lower wooded slopes of Dirtyface Mt, 2000', 6/24/32, Thompson 8540 (C,NY,SU,UW

Kittitas: S of Ellensburg, sagebrush slopes, 6/20/33, Thompson 9048 (C,CAS,NY,SU, UW

10 mi NW Ellensburg on rd. to Teanaway Jct., 5/22/44, Hitchc. & Muhlick 8180 (NY,UW

WASHINGTON, cont.

Yakima: along Logy Ck, Hwy 97, 5/8/38, Hitchcock & Martin 3383 (C,SU,RM,UW

Natches region, Cascade Mts, 6/1910, Grant (C

North Yakima, 5/31/02, Griffiths & Cotton 40 (C

Yakima, 7/3/98, Leckenby (US

Clallam (?): Olympic Mts, July, 1900, Elmer (CAS !

Klickitat: dry ground, Columbia R, 6/83, Suksdorf (SU,NY

Falcon Valley (Nr. Bingen) dry rocky places, 5/27/92, Suksdorf 1127 (C,NY

Bingen, 6/2/97, Suksdorf 2830 (SU, Minn, odd form with open panicle

R.R. bank several mi W Bingen, 6/11/20, Suksdorf 10512 (US

Skamania: Rands (near), dry hillside, 4/30/38, Hitchc. & Marsh 3299 (C,SU,RM,UW

OREGON

"E.Ore.", moist basaltic soil, 1200 m, 6/06, Cusick 3043(SU, Canbyi at US
do, dry basaltic soil, 4000', 7/4/06, Cusick 3148 (RM 3149 (RM

Wallowa: Imnaha Canyon 3 mi above Imnaha, 6/3/33, Peck 17491 (SU dry slope

do, 20 mi above Imnaha, dry woods, 7/8/33, Peck 17531 (NY,SU

Bald Knob, Imnaha NF, 6500', 6/23/07, Sampson & Pearson 30 (US
Crow Ck, 4320', 7/3/97, Sheldon 8505 (NY

Wallowa (?): Snake R. Landing (E. Ore.), sandy bank, 5/27/01, Cusick 2524 (C,Min NY,RM

Dry Creek, 3600', 7/8/07, Jardine 68 (US

Sherman: Grass Valley, moist ground, 6/18/25, Peck 13762 (F abnormal spikelets with 10-12 rather well-spaced florets per.

mouth John Day R, grassy hillsides, 5/5/25, Henderson 5084 (CAS,SU

John Day R, 5/17/85, T Howell (NY

Grant: Dayville, 5/85, T Howell (NY

Izee, creek bottoms, 7/15/02, Griffiths and Hunter 209 (US do.,do., river, do., G & H 233 (US

Wasco: Rowena, 6/99, Leckenby (NY

along Columbia R nr Rowena, sandy slope, 7/1/33, Peck 17422 (NY,SU

bet. Mosier and The Dalles, dry ground, 5/27/28, Thompson 4320 (SU

The Dalles, dry ground, 7/2/27, Thompson 2854 (SU

dry slope, canyon of Des Chutes R nr Maupin, 5/28/33, Peck 17340 (NY,SU

canyon N of Tygh Valley, rather dry slope, 5/29/27, Peck 14901 (SU

W of Friend, Oregon NF, rock crevices, yellow pine, 6/28/17, Lawrence 214 (SU

Deschutes: Bend, 7/3/21, Howell 7155 (CAS

OREGON, cont.

Harney: Alvord Ranch, 7/5/30, Jones 25184 (CAS,SU Henderson 8220,8222 (CAS,US

Alberson, moist ground, 7/2/25, Peck 14149 (SU

hillsides, Whiting Ranch, Blue Mts, 30 mi N Burns, 6/26/27, Hend. 8225 (CAS very scabrous lemmas

Lake: Summer L, in marsh, 6/20/19, Ferris & Duthie 384 (SU

6 mi NW Paisley, dry woods, 7/15/27, Peck 15666 (SU,UW

Klamath: nr Little Klamath L, 4500', 5/11/98, Appleg. 2047 (SU on rocky hillside toward nervosa

3 mi N Klamath Falls, basalt slopes over Klamath L, 7/31/46, Maguire & Holmgren 26518 (C,CAS,SU,UW

1/2 mi S Klamath Falls, ungrazed land, 5/11/40, Hitchc. 6385 in part, (NY,SU,RM

Klamath R, 10 mi below Spencer Ck, 5/13/98, rocky soil under Garry Oak, Appleg. 2074 (SU

Co.?: Deschutes River, 5/9/85, T Howell (NY

CALIFORNIA

Modoc: nr. Ash Ck, 10 mi E Adin, 5/26/36, Whitney 3157 (C

nr. Bull's Eye L, 7/15/35, Whitney s.n. (Veg

nr. Lost L, Warner Mts, 8000', 6/14/34, Howell 12171 (CAS,NY

Siskiyou: Devil's Homestead, Lava Beds N.M., 5/22/36, Appleg. 10347 (SU,US

Siskiyou(?): dry cliff, Klamath R, 7/16/08, Butler 467 (C,US toward juncifolia

Lassen: mts. S of Dixie Valley, 7/5/94, Baker & Nutting (US

mts. N of Moulton (NE of Ravendale), ca. 6000'(?), 8/5/02, Griffiths & Hunter 463 (US

Halls Flat, ponderosa-Jeffrey pine site, 7/2/44, Gus Hormay (C

Honey L. Valley, 6/97, Davy 3319 (SU

1.5 mi W Susanville, 4400', light soil, lava crevices, 7/8/44, Keck 5449 (C,CI, NY

Sierra: Mt. Lola, 7/18/01, Kennedy & Doten 182 (US

Nevada: Castle Peak, 7/18/43, Howell 18548 (CAS

**Placer: Tallac, 6/28/00, WR Dudley (SU unusual form with 8 florets per spikelet**

Mono: trail to Desert Ck, e of Swamp Maws, Sweetwater Mts, 9400', 8/9/45, Alexander & Kellogg 4568 (C,CI

Bridgeport, 8/5/45, Mullen (CI 130 cm. tall

Twin Lakes, Mammoth Lakes region, 8550', 7/20/35, Rose 35397 (CAS

borders of Mono L, 6/6/-Sept. 1866, (Bolander) 6113 (C a sheet at US, said to

CALIFORNIA, cont.

Mono, cont.

Jeffrey pine and aspen woods nr. Robinson Ck, flats above upper of Twin Lakes W of Bridgeport, 7400', 8/2/45, Ferris and Lorraine 10977 (SU

Poa Canbyi (Scribn.) Piper--page one

Type statement: "Cascade Mts., Washington Terr., Frank Tweedy and T. S. Brandegee, August, 1882."

EASTERN QUEBEC. Bic, Rimouski Co., crevices of dry, esposed calcareous rock, 7/8/05, Collins and Fernald 29 (NY seems OK in every way

YUKON DISTRICT. Moosehide, Mt. Dawson, 3700', 7/14/02, JMacoun 54545 (F,NY fide Hultèn; OK, DDK

**MACKENZIE. Great Bear L, Sawmill Bay, 7/24/48, Corcoran 9 (RM**

SASKATCHEWAN. Charlot Pt, L. Athabasca, 59°36'N, 109°13'W, rocky hillside, 6255 (CAS,NY) 7/2/35, Raup 6410 (NY ); do, 6/22/35, Raup 6289 (CAS,NY); do, 6/21/35, Raup

Touchwood, 7/16/06, Macoun & Herriot 77191 (NY

Lipton, prairie, 7/6/11, Clokey 1610 (NY

Moose Jaw, 6/17/96, JMacoun 13306 (NY approaching juncifolia

Herzel, 7/11/06, JMacoun & Herriot 77188 (NY

ALBERTA. below Laggan, Rocky Mtn. Park, 7/13/04, Macoun 64863 (US

Jasper Park, (NY

**Sage Creek, Milk R, 7/8/95, JMacoun 13305 (NY Milk R, 7/12/95, JMac 13303 (NY**

Crow's Nest Pass, 7/29/97, JMaccoun 33980 (NY do, 8/18/97, JMacoun 33981(NY

do, 8/13/97, JMacoun 34007 (NY

Craigmyle District, Brinkman 219 (NY do, NW28,32,16W4, 2750', 6/23/21, Brinkman 136 (NY do, do, do, 6/30/21, Br. 163 (NY

Elbow River, lat. 49°40', 7/16/97, JMacoun 34000 (NY

BRITISH COLUMBIA. Prince Rupert (?): Ca. 1500' above head of Dease Lake (130° W.Long., ca. 59°N. Lat.), in pockets of gravelly soil in bare dry volcanic rock at timberline, in small dense coherent clusters, 8/4/41, McCabe 8736 (C typical Canbyi, 48 cm. high, basal lvs to 17 cm. long, lig. 4 mm., acuminate, lemmas slightly pubescent, panicles 6-6 cm. long, slender, purple. Like a tall incurva, and could be considered that. Certainly not at all as stout as the following two.

Caribou(?): 1000 ft. above Stine (Fraser R. Valley??), 6/30/34, McCabe 1558B(C

Caribou: alpine slopes of Green Mt, nr. Haylmore, 7000', 7/28/38, JW & EM Thompson 668 (C

Kootenay: Yoho Park, along Ottertail road, 2 mi W of Field, 4600', 7/3/27, Ulke 631 (F

**Rogers Pass, Selkirks, 4000', JKHenry 559 (RM**

**NORTH DAKOTA.**

**Ramsay: Devils Lake, prairies, saline soil, 6/29/02, Lunell (RM**

Billings: Medora, 7/16/37, Moran 315 (C

SOUTH DAKOTA. **? Co.: Buffalo Gap, red foothillsm grass,assoc, 6/27/27, Hayward** 1538 (RM

Custer: Custer, Black Hills, 5500', 7/16/92, Rydberg 1149 (NY

Meade: Elk Canyon, Black Hills, 4-5000', 6/29/92, Rydberg 1146 (NY

Fall River: Hot Springs, 3500', 8/3/92, Rydberg 1146 (NY

SOUTH DAKOTA, cont.

Co.?: 10 mi NW Deerfield, 7/3/40, Johnson 121 (NY !

MONTANA. without loc., 1883, Scribner 53 (US, type of P. laevis Vasey

Co.?: North Fork Smith R, 5600', 7/19/83, Scribner (NY isotype of P. laevis Vasey and P. laevigata Scribn.

Mt. McDougal, Kootenai Mts, (Big Fork), 8/11/01, Umbach 126 (NY

Fergus: Denton, 6/29/01, Spragg (?) (C ; Square Butte, 7/16/01, Spragg (RM
Sweetgrass: Upside-Down Trail, nr. Boulder River, 7/17/47, Hitchc. 16538 (UW
Wheatland: river bottom at Harlowton, 6/16/34, Hitchcock 2344 (CAS
Powell: grassland, 15 mi. W. of Lincoln on Orando Rd, 6/28/45, " & Muhlick 11632 (UW
Stillwater: dry open slopes, summit Haystack Mt, hd Boulder Ck, Absaroka NF, 10500', 8/8/45, Hitchc. & Muhlick 13400 (CAS not incurva?

Cascade: Great Falls, 6/87, Anderson 619 (SU 8/5/90, Williams (RM

Judith Basin: Yogo Baldy, Little Belt Mts, 7000', 8/24/96, Rydberg 3421 (NY 3417 (NY 3427 (NY

do, 7000', 8/24/96, Flodman 130 (NY

Lewis and Clarke: Helena, 1888, Kelsey (NY, SU do, 1889 (NY

Meagher: Elk Mts, nr Black Hawk, 6000', 8/5/96, Flodman 132 (NY
Link Park, 3 mi. E. of Williams Mt., Little Belt Mts, 7/12/45, Hitch. & Muhlick 12291 (UW
Broadwater: Townsend, 7/15/95, Rydb. 2158 (NY

Gallatin: Bozeman, 6/19/02, Blankinship (C

Park: Livingston, 5000', 6/8/06, Blankinship 799 (C, RM

Electric Peak, 8000', 8/18/97, Rydberg and Bessey 3663 (NY incurva? short leaved, called Buckleyana, inflor a little open but not much like gracillima--toward P. incurva = nervosa@ RM

Madison: Gravelly Range, 6 mi NE of Crockett Lake, 8000', 7/19/45, Hitchc. & Muhlick 12483 (CAS, SU[UW = ample @ UW], RM
East Hammond Ck, 13 mi. E. of Ennis, 7/23/47, Hitchc. 16748 (UW
Spanish Basin, 6000', 7/20/96, Flodman 149 (NY
do, 7/18/96, Rydberg 3147 (NY

Spanish Creek, 7/11/96, Rydberg 3025 (NY

Cliff Lake, 7000', 7/27/97, Rydb. and Bess. 3636 (NY
Glacier: 14 mi. S. of St. Mary, 7/9/41, Fry 511 (UW
Glacier NP: Sperry Trail, 6000', 7/27/19, Somes 10 (NY
**Gunsight Pass, 7250', 7/15/19, MPSomes 54 (NY toward incurva**
open ground, McDonald Ck. and Little Kootenai, 7000', 7/8/14, Hitchc. 11258 (US

Flathead: Columbia Falls, 7/9/94, Williams (NY

Beaverhead: Monica, 7/27/22, Jones (US

WYOMING.

Co.?: Camp Crawford, 8/6/08, Clemens (SU

[7/14/27, Hayward 2160 (
WYOMING, cont. **Crook: Inyankara Ck, 17 mi. S. Sundance, creekside, grass assoc,**

[~~Goshen: Raw Hide Butte, Buffum 5115 (NY~~ = juncif. Porter]

Albany: Medicine Bow Mts, 7/28/99, ANelson 7739 (NY, RM
**Medicine Bow Forest, timberline bunch grass, 10,000', 7/14/36, Porter 2170(**
Telephone Mines, 8/1/00, ANelson 7886a (NY, RM

2514 (RM orig.
[Veedauwoo Rocks, Laramie Mts, 8500', 7/13/38, Porter 2512 (C, RM = Sandbergii]
**Libby Flats, Medicine Bow Mts, 11000', 8/18/38, Porter 2610 (RM**
head of Pole Ck, 6/19/97, ANelson 3197 (NY
**Halleck Canyon, sandy creek bottoms, 7/4/00, ANelson 7431 (RM**
Laramie, 7/22/01, E Nelson 431 (C, RM — in alkaline mdw. - not junciful. Porter
do, 6/28/26, Heller 13960 (SU    7/14/01, E Nelson 402 (RM, NY
**Pilot Knob, dry sandy soil, 8500', 8/5/44, Porter 3469 (RM**
Route 85, 5 mi E of jct. with Route 87, dry banks along highway, 5/24/47,
Beetle 4416 (SU

Laramie Hills, 7/19/01, E Nelson 416 (C, RM    6/16/97, A. Nelson 3179 (RM

Centennial Val, E margin Medicine Bow NF, 8/2/97, Nelson 3277 (C

Chimney Park, 8/1/01, E. Nelson 463 (SU, RM not junci. - Porter

Pole Mtn region, 8400', 7/6/43, Porter 3249, in part (NY, SU    = ampla at
do, 8/15/42, Porter 3166 (NY do, 8200', 8/13/44, (C, CI n. sp.? Porter 3510 (N

Sheridan: Big Horn, in clay soil above, 6-7800', 7/97, LHPammel (US, type of P. wyomingensis Scribn., spikelets crowded and large, up to 7 florets plus a rudiment!
**Tongue District, Bighorn NF, 8000', 8/3/52, Hurd 313 (RM**
Sheridan, 6/25/97, Pammel & Crone 119 (Minn

foothills betw. Sheridan and Buffalo, 6000', 6/15-7/15/00, Tweedy 3698 (NY, RM
rolling plains " " " " " 3695 (RM, NY
Big Horn Mts, 8000', 7/99, Tweedy 2136 (NY

Johnson: E slope Bighorns, hdwts Clear Ck and Crazy Woman R, Tweedy 3700 (NY, [illegible]

Buffalo, N. Fork Clear Ck, 7500', 7/27/98, Griffiths & Williams 21 (C
**5 mi. E. of Powder R. Pass, Big Horn Mts, 9000', 8/6/47, Porter 4385 (RM**
~~Buffalo Fork, Tweedy 19 (NY~~ = juncif.

Carbon: Park Hdqts, Medicine Bow NF, 7/14/36, Porter 2168 (C RM    9500'
**Battle Lake Mt, 8/17/97, ANelson 4043 (RM    typical even tho late**
Copperton, 8700', 8/1/01, Tweedy 4345 (NY

Chimneys of Pedro Mts, 6/25/01, Goodding 111 (C, CI, NY, SU, RM = gracyana
**Big Horn: Doyle Creek, moist alpine mdw, 7/26/01, Goodding 383** √ **(RM** looks like
Big Horn: hd. Mid. Fk. Powder R, 7/18/01, Goodding 284 (NY) SU, RM 371 - Sandbergii
**Fremont: Sweetwater R. on Farson-Lander road, 7200', 7/7/49, Porter 4981 (RM**
**Fremont:** (Lander, (NY = nevadensis) Fiddlers L. Wind R. Mts, w. of Lander, 9000', 8/1/50, Porter 5584 (RM
**Sweetwater: Rock Springs, common, 6200', 5/20/47, Larsen 3 (RM    long-lvd.**
Sweetwater: Leucite Hills, N of Rock Springs, 6/17/01, Merrill & Wilcox 3 + 15 (RM, NY
(F, SU, RM Red Desert, 6/3/97, A. Nelson 3118 (NY    do, do, do M & W 10
Park: Lookout Station, 3 mi. W. of Beartooth L, 7/20/47, Hitchc. 16652 (UW    (NY (could be Sandbergii-no base)
**Sweetwater: Point of Rocks, 6/1/97, A.Nelson 3093 (RM**    long lvs. - not Sandbergii
"    J. Johnson's Ranch, 3/5/97, A. Nelson 3106 (RM
Yellowstone Nr: Yancey's, 7/17/99, Nelson 5941 (C, Minn, SU, RM, NY
**Undine Falls, open hills, 7/18/99, A & ENelsom 5963 (RM**
E. De Lacy's Ck, 7500', 8/10/97, Rydberg & Bessey 3002 (NY
**Dunraven Peak, 8/27/99, A & E Nelson 6927 (RM**

Poa Canbyi (Scribn.) Piper--page four

WYOMING, cont.

Yellowstone NP, cont. **Lewis R, small tufts, open woods, 8/21/99, ANels & ENe** 6593 (RM,NY

Mammoth Lake, Hot Sprgs, open grassy slope, 6300', 6/27/38, Bauer 29 (US

Yellowstone Lake, 8/23/99, A & E Nelson 6625 (C,NY,SU,RM
do, 7500', 8/12/97, Rydberg & Bessey 3664 (NY,RM

s. part YNP, rocky slope below cliff, 7/28/26, Hitchcock 23152 (US

Teton: Jackson, Gros Ventre R, 7/22/01, Merrill & Wilcox 307 (C,NY,RM = gjuncifo! but ligule short!
Jackson Hole (NY Jackson, dry fields, 7/13/01, " " " 264 (RM lig rather

Sublette: Big Sandy, 7/21/92, Buffum (NY
**Horseshoe L, W. slope Wind R. Range se. of Pinedale, 9000', 7/30/52, Porter & Miller 6102(R**

juncif. [Lincoln: Hams Fk and La Barge, 7000', 7/13/00, Curtis (NY ]

Uinta: Snake River, abundant in mdws, 8/15/99, A Nels. 6462a (NY,RM 6461(RM better as nevad. @NY

COLORADO.

Larimer: Pinkham Ck, 7/7/03, Goodding 1476 (NY,RM toward Sandbergii

Beaver Ck, 7/4/03, Goodding 1449 (C,NY,SU " "
do, 8/7/92, State Agric. Coll Colo 3766 (NY ; **do., 9000', 7/8/96. do 3757(R**

Rabbit Ears, 7/15/03, Goodding 1553a (NY

Holridge Mdws, N. Park, 7/22/94, State Agric. Coll.Colo 3758 (NY

Encampment Mdws, North Park, 8200', 7/6/01, Tweedy 4343 (NY toward Sandbergii (or incurva)

**Higho, 9000', 8/17/98, Shear & Bessey 1497 (C**

Eagle: McCoy, 6500', 7/30/98, Shear & Bessey 1326 (C = ampla at US
**Boulder: Boulder, 5/21/11, Robbins 8660 (RM**
Jefferson: foothills nr. Golden, 6500', 6/17/78, Jones 255 (US

Grand: Milner Pass, 3340 m, 8/11/21, Clokey, Bruderlin, Clokey 4008 (CAS,SU,RM

Sulphur Springs, 6/28/05, Osterhout 2994 (NY,RM

**1 mi NE Columbine Lake, Rocky Mt. NP, 8700', 7/29/36, Sawyer & Rutter 73 (C**

Summit: Breckenridge, rare, wet woods, 10000', 8/29/96, Clements 389 (US
**Mt. Baldy, nr. Breckenridge, 12,500', 8/01, Mackenzie 343 (RM,NY**
Lake: Twin Lakes;

El Paso: Table Rock, 7500', 6/27/91, Crandall 8 (US

**dense clumps, gravel, edge spruce woods nr. Seven Lakes, nr. Pikes P,** 10000', 9/1/08, Chase 5329 (US

nr. lake beyond Windy Point, Pikes Peak, 9/3/06, Hitchcock 2342 (US

Clear Creek; mt. sides at Georgetown, hdwtrs Clear Ck, 8500', 7/3/85, Patterson 73 (C,F (rock at top of Devil's Gate fall), isotypes,US, type of P. lucida Vasey

COLORADO, cont.

Clear Creek, cont.

Silver Plume, 8/21/95, Rydberg 2428 (NY  do, do, Shear 680 (RM

Mt. sides nr. Empire, 8500-11000', 9/6/92, Patterson (F

Chaffee: Buena Vista, 10000', 8/20/96, Shear 1018 (NY very small-fld.

Park: Como, South Park, 8500', 8/7/86, Letterman 47 (US

Hoosier Pass, S. side, 10000', 7/28/35, Penland 1319 (CAS

South Park few mi W of Hartsel, ca. 9000'?, 7/7/40, Penland 1559 (CAS

Gunnison: 5 mi S Tincup, 11000', Rollins 1438 (C,SU
Crystal Creek, 8000', 6/27/01, Baker 262 (NY
[Gunnison, 7/18/01, Baker 467 (NY fide V.L.M. = junci folia gracilenta]

Delta: Leroux Ck, 9000', 7/14/92, Cowen (NY

Montrose: Cimarron, 6900', 7/5/01, Baker 329 (NY lvs. basal and short (6-9 cm) but not incurva (culms too husky and 30 cm) and poor Sandbergii

Montezuma: Mancos, 7000', 7/8/98, Baker, Earle, Tracy 434 (C,F,Minn,RM
do, 6/23/98, do, 110 (NY,RM
Chicken Ck, W. Mt. Hesperus, 9000', 6/26/98, Baker, Earle, & Tracy 941 (F

Bob Ck, W of Mt. Hesperus, 10500', open mdws, 6/30/98, Baker, Earle, & Tracy 266 (US - grayana at C,F,NY with rhizomes!

?Co.: Fort Garland, San Luis Valley, 7937', 1884, Vasey (US

IDAHO.

Fremont: Mt. Chauvet, 10,000', 7/29/97, Rydberg & Bessey 3655 (NY

along river at St. Anthony, 7/4/01, Merrill & Wilcox 140 (C,NY,SU

Clark: Beaver Ck, 6000', 3 mi above Spencer, 6/24/39, Cronquist 1294 in part (NY (with ampla)

Teton: Victor, common in dry soil, 7/10/01, Merrill & Wilcox 177 (RM 7/11/01, 204 (RM

Boundary: granitic cliffs above Smith Ck, 5000', 7/14/36, G.B.&R.P.Rossbach 757 (CAS,SU,UW

Latah (or Benewah): Upper St. Mary's R, 970 m, Coeur D'Alene Mts, 6/28/95, Leiberg 1104 (SU,NY

Shoshone: divide bet. St. Joe and Clearwater R, 1900 m (St. Joe NF), 7/11/95, Leiberg 1237 (CAS,SU

Clearwater: Orofino Grade 2 mi NW of Orofino on rd to Ahsahka, 5/28/44, Hitchcock & Muhlick 8425 (NY too big for Sandbergii!?

Lemhi: Gibbonsville, 5500', yellow-pine forest, 6/22/38, Hitch. et al 3690 (CAS

Oneida: Malad Holbrook Hiway, summit, 6000', 6/11/41, Hull 207 (NY

IDAHO, cont.

Nez Perces: nr. Lake Waha, 2-3500', 6/20/96, Heller 3274 (C,NY,SU isotypes of P. Helleri Rydb. At NY I took this at first for an overgrown Sandbergii, at least the spring-flowering thing, but I let it go as Canbyi finally.

about Lewiston, 1500-2000', 5/11/96, Heller 3042 (C very robust

Custer: Mahogany Ck, Lost River Mts, nr. Mt. Borah, 8/12/44, Hitch. & Muhlick 11060 (CAS SU=incurva) 11055 (CAS,NY,SU,UW,RM

MacKay, 5887', 8/1/11, Nelson and Macbride 1539 in part (F (with nervosa)

Sunbeam, 12 mi E of Stanley, under pines, 6/16/44, Hitch. & Muhlick 9013 (CAS,NY,SU,UW,RM toward Sandbg.

gravel wash ca. 20 mi S of Challis, nr. Salmon R, 6/15/44, Hitch. & Muhlick 9000 (NY a big leafy Canbyi

betw. Stanley L. and Cape Horn, 7/7/44, H&M 9663 (UW

Blaine: hd. Boulder Ck. Can., Sawtooth Mts, Sawtooth NF, 10000', 8/2/37, Thompson 14078 (C,SU,NY

mdwland 5 mi N Galena Pass, 7/31/44, Hitch. & Muhlick 10552a (NY

sandy mdwland on Norton Ck nr Baker Ck, vic. Norton Pk, Smoky Mts., 8/3/44, Hitchc. & Muhlick 10732 (CAS,NY,UW

Picabo, 4900', 6/30/16, Macbride & Payson 2959 (C,CAS,SU,RM,NY

Butte (?): Big Butte Station, 6/22/92, Palmer 211 (C,Minn,SU,RM
Bear Ck, 14 mi. N. Leslie, Lost R. Mts, 7300', 6/12/44, Hitchc. & Muhlick 8816 (RM =Cusickii in other herb.
Arco, 6/92, Palmer 179 (Minn,SU,RM

Bingham (or Bannock or Power): Fort Hall Indian Res, 1932, Shoemaker (US

Jerome: Shoshone Falls, 6/4/92, Palmer 88 (SU,RM = Sandbergii (long-lvd.)
do, 6/4/92, Palmer 154 (C

Blue Lake (Jerome?), 6/1893 (?), Palmer 87 (C E. Palmer 86 (US (6/3/92)

Valley: Gold Fork Lookout, Payette NF, Sawtooth Mts, 8100', 7/9/37, Thompson 13771 (C,SU

Adams: 20 mi. S. of New Meadows, nr. hiway to Weiser, 6/24/46, Hitch & Muhlick 13878 (UW

Boise: talus above Payette R, 5 mi N of Banks, 5/31/44, Hitchcock & Muhlick 8553 (CAS,NY these are too robust for Sandbergii

above Payette R 15 mi W of Lowman under yellow pines, 5/31/44, H & M 8564 (NY

Owyhee: Silver City, 7000', 6/20/11, Macbride 949 (F,SU,RM -Sandbergii?

UTAH.

Summit: Black's Fork, N. slope of Uinta Mts, in scattered lodgepole and aspen, 9000', 9/3/45, BF & Kent Harrison 10945 (C short, broad lvs.

Uintah (?): Young Spring's Dugway, Uinta Mts, among dead timber, 7/16/02, Goodding 1405 (NY,RM

Poa Canbyi (Scribn.) Piper--page seven

UTAH, cont. **Cache: Providence Bench, 4800', 5/27/32, Maguire 3239 (RM**

Duchesne: hd. Blind Stream, Uintah Mts, 10500', 7/3/38, Harrison & Nisson 8801 (C **Harrison, Liechty & Allen 10009 (RM**

**just below 1st chain lake of Chain Lakes, Uinta Mts., 10,500', 7/27/40,**

Juab: Mt. Nebo, 8/15/05, Rydberg & Carlton 7597 (NY, RM

San Pete (?): laboratory to summit (Ephraim Canyon?) 8700-10,000', 8/22-27/13 Hitchcock 10912 (US

Tooele: Lake Point, 4200', 7/19/79, Jones 1021 (NY,SU

Utah: Pleasant View, 4700', 5/26/38, Harrison 8347 (C

Thistle Jct. 5000', 6/10/00, Stokes (NY

Sevier: Fish Lake Plateau, 8/9/05, Rydberg & Carlton 7691 (NY (almost incurva)

nr. Fish Lake, 9000', 8/11/94, Jones 5826 (NY

Wayne: Thousand Lake Mt, 10500', 7/14/75, Ward 390 (F

Garfield: S. rim Mt. Ellen (Bull Ck.) Basin, Henry Mts., 10,000', 7/2/40, Maguire 19375 (RM could pass for incurva, but in flor. too narrow for gracillima

Iron: Brian Head Point, Cedar Breaks Nat. Mon. nr n. border, 11000', 7/29/40, Ferris 10288 (SU

Brianhead Mt, Cedar Breaks, 8/15/38, Silveus 3324 (CAS,US

S18,T36S,R8W, 1 mi NW Louder G.S., 10500', 9/8/42, Ellison 942-1 (US

Millard: 50 mi W of Hinckley, 4500', 5/10/35, Harrison 6339 (US (Atriplex-tetradymia assoc)

Washington: Midway Ck, 8/14/37, Gierisch 522 (NY !

NEVADA.

Elko: Martin Ck nr Gold Ck, 7000', 8/8/13, Kennedy 4435 (SU

E. Humboldt Mts, 8/2/01, Jones 11146 (US called gracillima by ASH but too big for that

Coon Ck, Stampede, 8/9/02, Kennedy 632 (RM

Victory Hiway E of Wells, 6/11/33, Eastwood & Howell 312a (CAS stout!

Victory Hiway 10 mi E of Battle Mtn, 6/10/33, Eastwood & Howell 150 (CAS

Jack Ck, 70 mi NW of Elko, 6500', 6/25/37, Nichols & Lund 189 (SU

Jarbidge, 7000', 7/12/12, Nels & Macbride 2030 (Minn, NY, RM

**White Pine: ~~ridge N. Lehman, Snake Range, 6/17/41, Maguire 20786 (C~~**

**N side Mt. Sherman, base of cirque, 10000', 8/4/39, Hitchc. & Martin 5666** (NY very scabrous

Mineral: Big Indian Mine rd to Cory Can, 9500', 7/12/45, Alex. & Kellogg 4446 (C,CI

Cat Ck, Wassuk Range, 8600', 7/15/45, Alex. & Kellogg 4464 (C,CI

**Mt. Grant Grade, betw. Cottonwood-Laphan Mdw. divide and Mt. Grant Pk, Wassuk Range, 9700', 7/6/40, Train 4256 (NY**

NEVADA, cont.

Lander: Big Ck, Toyabe Range, 7000', 7/27/13, Kennedy 4045 (SU

Nye: Mohawk R.S., 8000', 6/17/31, JM & MAR Linsdale 229 (CAS   toward Sandbergii

Washoe: Reno, 4500', 6/8/97, Jones 11433 (SU

do, 5000', 6/19/00, Stokes (SU

Hunter's Ck. Canyon (Dinsmore Camp), 6000', 6/20/07, Kennedy 1639 (C

Hunter's Ck. road, 9-11 mi SW Reno, 7000', 7/11/38, Archer 6288 (SU, NY

Mt. Rose, 10000', 8/17/05, Kennedy 976 (C, CAS

WASHINGTON

Okanogan: Oroville, 6/27/11, Jones (SU

Barnhard Trail W of Salmon Mdws, Mt. Tiffany, 7/17/32, Fiker 1034 (SU

Asotin: dripping cliff along Buford Ck, 1 mi above mouth, S2 T31N, R44E, 5/30/48, Keck 6034 (CI

lower pt of Rattlesnake Grade, S of Anatone, 5/30/48, Keck 6033 (CI

Garfield: above Tucannon R betw. Clearwater RS and Mt. Misery, Blue Mts., T8N, R42E, S15, 6/6/38, Peters 358 (C

Chelan: Leavenworth, 5/23/31, Thompson 6422 (SU

Kittitas: Mt. Stuart region, 5000', 7/27-31/31, Thompson 7819 (SU

scabby hilltops, hd. of Quillamene Ck, 4000', 6/21/04, Cotton 1601 (US

nr. Virden, 6/8/35, Thompson 11594 (SU, US    sagebrush scabland

Yakima: Mt. Paddo (Adams), 2000 m, 7/12/00, Suksdorf 2834 (F, SU, US,
do, stony slopes, 2300 m, 7/27/06, Suksdorf 5746 (US
do, 2000 m, 8/31/04, Suksdorf 4161 (F, SU

Klickitat: nr. Bingen, 5/14/98, Suksdorf 2831 (SU

Scott, 6/5/98, ABLeckenby (US, type of P. Leckenbyi Scribn. def. not scabrella

Skamania (?): La Camas Lake, 5/31/84, Henderson 1158 (SU

Co.?: Blue Mts, 5/15/97, Horner 548 (US

"Glyceria Canbyi Scribner n. sp." bogs, 2500', Yakima Region, July, 1882, Frank Tweedy (NY    not considered isotype by me

common in swamps, Washington Terr., 1883, TSBrandegee 1190 (US, marked "
"dupl. type of G(lyceria) Canbyi" by A.S.H., but it is not!

OREGON

Wallowa: Ice L, 3000 m, 7/14/34, Peck 18535 (NY, SU, WU

OREGON, cont.

Wallowa, cont.

Ice L. trail, 6/26/36, Eastwood & Howell 3375 (CAS

nr. Aneroid L, 7/28/33, Peck 17910 (SU

open S slope, val. of Davis Ck, Imnaha Forest, 6/26/07, Coville 2447 (US, NY

Pete's Point, 7/27/33, Peck 17959 (SU

summit Imnaha-Snake Divide 23 mi above Imnaha, 7/10/33, Peck 17560 (SU

Co.?: "Eastern Oregon", 3500', 6/23/99, Cusick 2198 (C
do, Cusick 2200 (C    do, Cusick 3043 (US

Umatilla: dry streamlet, pine woods, Blue Mts N of Albee, 3500-4000', 6/24/08, Chase 4800 (US

Hood River: Hood River, 5/27/27, Peck 14843 (SU

Grant: Blue Mt. nr. Bates, 4200', 6/5/25, Henderson 5352 (CAS, SU

Co.?: W Blue Mts nr Ukiah, 4500', 6/18/10, Cusick 3447 (NY, RM

Grant: about Austin, 4200', 6/9/25, Henderson 5344 (CAS, SU

Wheeler: Upper Service Ck, 5/4/25, Henderson 5087 (CAS, SU

Douglas (?): Bristow Prairie, Umpqua Forest, 5000', rocky slope, 8/1/27, Hitchcock 23513 (US

Malheur: Owyhee Divide, 1300 m, 5/31/96, Leiberg 2181 (C, RM, NY

Harney: Wild Horse Ck, E side Steens Mt, 5/22/29, Applegate 5656 (SU

ck. nr. divide of Steens Mt, above Anderson Val, 6/6/27, Henderson 8199 (CAS

Steens Mts, 7000', 6/18/01, Cusick 2581 (C, RM, NY [called Sandbergii → Canbyi @ RM]

French Glen, nr. P-Ranch, Donner & Blitzen R, 5/20/27, Henderson 8240 (CAS
do, high slope, 6/27/42, Peck 21409 (CAS

Sawtooth Ck, nr. Burns-Izee rd, open spots in yellow pine timber, 6/17/27, Henderson 8215 (CAS    do, 6/18/27, Henderson 8217 (CAS "Blue Mts. of Harney Co."

flat nr. Crane, 6/4/27, Henderson 8206 (CAS

Lake: Forest Camp on Dairy Ck, 35 mi NW Lakeview (Fremont NF?), 7/1/27, Peck 15423 (SU

nr. Chewaucan Marsh, 1330 m, 6/2/11, Eggleston 6779 (US

Dog Lake R.S., Fremont Forest, 5500', 5/26/30, Ellis (US

Klamath: Keno, 7/6/20, Peck 9339 (SU

do, 7/7/20, Peck 93[illegible]2 (SU

OREGON, cont.

Jackson: Cascades, 7/9/02, Cusick 2865 (C,SU

Siskiyou Summit, 4600', 6/23/29, Kildale and Gillespie 8292 (SU

CALIFORNIA.

Siskiyou: Capt. Jack's Ice Cave, Lava Beds NM, 6/5/35, Applegate 9341 (SU

Indian Well, Lava Beds NM, 6/10/35, Applegate 9465 (SU

Bearpaw Cave, Lava Beds NM, 6/8/35, Applegate 9424 (SU
do, 6/8/35, Applegate 9434 (SU

Grenada Sta, 6/19/05, Heller 8070 (SU =fibrata?

Modoc: Mill Ck. Mdws, 7/31/32, Applegate 7976 (SU

Duncan Horse Camp, 7/1/34, Howell 12341,12342,12343,12344,12345,12346,1234[illegible]
(CAS
do, 12346 (NY

Parker Ck, Warner Mts, 5200', 6/11/34, Howell 11927 (CAS

Warner Mts, Modoc NF, 3 mi from Patterson RS, road to Eagleville, 8/14/41,
Ferris and Lorraine 10582 (C,SU

Trinity: S. shore Upper Canyon Ck Lake, 11 mi N. Dedrick, Salmon-Trinity
Mts, 6200', 7/11/39, Hitchcock and Martin 5393 (SU

Scott Mt, Siskiyou-Trinity line, 7/29/37, Howell 13620 (CAS

Devil's Canyon Mts. hd. of White's Ck, 6800', 8/5/35, Tracy 14549 (SU (16619
**N. slope of Nobth Yolla Bolly Mt, Tehama Co. line, 6500', 7/18/51, Munz (NY**

Shasta: nr. McArthur, swale in open lava flow mdw, 5/27/40, Hitchcock 6034
(NY too husky for Sandbergii

Lassen: Dixie Val., 7/3/ (1894?), Baker (C
do, Baker, Nutting in 1894 (C

nr. Lost Lake, Warner Mts., 8000', 6/14/34, Howell 12173 (CAS,SU

Mono: SW of Bodie, 8500', rocky outcrop, 6/27/45, Alex. & Kellogg 4327 (C,CI
(CI could definitely go as incurva!) all incurva?

saddle S. side Masonic Pk, 1.5 mi from Masonic Spr, 8600', 8/4/45, Ferris
and Lorraine 11031 (C,SU all incurva?

(additional note for above two specimens: OK as Canbyi but very scabrous,
**glaucous, involute and lemmas very attenuate)**

Poa gracilima Vasey - page one

ALBERTA

nr. Banff, lat. 51°11', long. 115°34', 6700', 8/18/91, Macoun 18a (US

Mt. Edith Cavell, 6500', 8/23/17, JMMacoun 98634 (NY
Waterton Lakes N.P.

BRITISH COLUMBIA

Kootenay: open rocky soil, Burgess Pass, nr. Field, Yoho Nat'l Park, 8/1-2/14, Hitchcock 11518 (US
open slope below Burgess Pass, do., do., H. 11522 (US
trail to Burgess Pass, Yoho Valley, 8/28/04, Macoun 64858 (US

Asulkan Valley, Glacier, Selkirk Mts, 4100-6000', 7/19/06, Brown 619 (Ph,US

mdws, Kokanee Nat'l Park, 6000', 7/29/38, Eastham 40 (US

New Westminster: mdws. nr. Black Tusk Mt, Garibaldi Park, 40 mi. N.of Vancouver, 5000', 8/8-13/38, Eastham 18 (US do., do., at 5500', E. 10 (US the latter toward stenantha

Chilliwack Valley, 8/9/01, Macoun 26408 (US looks like scabrella

MONTANA

Glacier Nat'l Park: Logan's Pass, 6000', 8/1/38, Silveus 3187 (US
Glacier Camp, Lake McDonald, 8/21/01, Umbach 399 (NY
above McDermotts, Glacier NP, 7/14/14, Hitchc. 11297 (US

Gunsight Pass, rocky slope, 7/19/14, Hitchcock 11326 (US toward stenantha

alpine mdw, Iceberg Lake trail, 7/15/14, Hitchcock 11307 (US " "

Sperry Glacier, 6000', 9/1/03, Blankinship 3 (C,RM
7500', 8/27/09, MEJones (CAS
mt. sides, Sperry Glacier, 9/1/03, Umbach 850 (NY,SU,US,RM
Sperry trail, 6600', 7/27/19, MP Somes 12 (NY

Missoula: E. side Glacier Peaks above Turquoise Lake, Mission Range, 8-9000', 8/11/36, GB & RP Rossbach 762 (SU 761 (SU

Beaverhead: Lake Waukena, head of Rock Ck, Pioneer Mts, 8/1/45, Hitchcock & Muhlick 13125 (CAS,RM

WYOMING

Teton: W. side Jenny L, Jackson Hole, bare rocky slope, very glaucous, 7/26/26, Hitchcock 23129 (US

above Leighs Lake, Jackson Hole, 9000', nr. a mtn. stream, 7/26/01, Merrill & Wilcox 341 (US

COLORADO

? Co.: Four Mile Creek, Leadville Nat'l Forest, 11,000', 7/26/27, Oakley 17 (US (Forest Service 57756; seems to be very good gracillima)

IDAHO

Boundary (or Bonner): Lime Creek, Upper Priest R, 2700', July 1925, Epling 7622 (US

Smith Peak, sunny slope of, Smith Creek region, 6-7000', 7/16/36, GB & RP Rossbach 763 (SU

Nez Perce (?): grassy mts. slopes, divide betw. St. Joe and Clearwater R, 1900 m, 7/11/95, Leiberg 1237 (US

Idaho (?): Hd. of Bear Creek, 1850 m, Bitter Root Forest Reserve, 8/29/97, Leiberg 2957, 2959 (US

**summit of "He Devil", Seven Devils Mts, 8/10-15/38, A & RNelson 2963 (RM**

Valley: granite slopes, Gold Fork Lookout, Payette NF, Sawtooth Mts, 8100', 7/11/37, Thompson, 13,800 (C, CAS, SU, F, US 7/19/37, 13903 (RM

Custer: small mdw. below Frog L, N. base Castle Pk, White Cloud Range, Challis NF, 10,400', 8/8/44, Hitchc. & Muhlick 10823 (CAS, GH, SU, RM

Toxaway L, 10 mi. w/sw Obsidian, Sawtooth Mts, 8300', 8/8-11/39, Hitch. & Martin 5738 (C, SU

do, 12 mi. SW Obsidian, 9700', do, H & M, 5773 (C

Blaine: Sawtooth Peaks, 9000', 8/9/16, Macbride & Payson 3689 (C, CAS, NY, SU, RM

meadowland at lakes at hd. of Alpine Ck, NW. of Alturas L, Sawtooth Primitive Area, 7/30/44, Hitchc. & Muhlick 10475 (CAS, GH, NY, SU, RM

Elmore: granite outcrops nr. Big Roaring River Lake, 20 mi. n. of Pine, 8/24/47, Fred G. & Lillian E. Meyer 2307 (C perhaps as happy in incurva

**Trinity Lakes Region, granite pks, 8000', 8/29/10, Macbride 657 (RM**

UTAH **Uintah: Dyer Mine, timber, 7/2/02, Goodding 1230 (RM = nervosa @ C, F**

Summit: above timber, SW. slopes Bald Mt, 11,500', 8/14/33, Maguire 3992 (C, RM

SW. side Bald Mt, do., do., Maguire 4004 (C

Stillwater Basin, Uinta Mts, hd. Bear R, 10,500', 8/18/33, Maguire 4002 (US

**Utah: Boy Scout Falls, Timpooneke Sta. to Mt. Timpanogas, 8200', 8/10/39, Maguire 17488 (BYU ; se. slope Mt. Timp., 9500', 8/4/30, Harrison & Swallen 9331 (BYU**

WASHINGTON

**Okanogan: rocky talus slope in Angels Pass, Okanogan Mts. nr. Conconully, 5000', 6/28/31, Thompson 7043 (C, SU, US**

Chelan: Chiwaukum L, Wenatchee Forest, 1450-1600 m, 8/19/16, Eggleston 13592 (US

Do., 1600 m, do, E. 13562 (US

Indian Head Pk, Wenatchee NF, 5000-7411', 7/31/21, St. John 4816 (C

Tip Top Lookout, Wenatchee Mts, 4000', 6/19/31, Thompson 6830 (SU

Stevens Pass, 8/93, Sandberg & Leiberg 747 (US drawn for Hitchc. Manual

WASHINGTON, cont.

Chelan, cont.

Mt. Stuart, 7500', 8/20/30, Thompson 5784, 5787 (SU
among rocks on divide betw. Mt. Stuart and Teanaway Ck, 7/27/98,
Whited 846 (US

Three Brothers, Wenatchee NF, 7000', 8/19/35, Thompson 12630 (SU
lower alpine slope of Three Brothers, 5000', 6/21/34, Th. 10730 (US
rock slides at base of Three Brothers, 3000', 6/2/34, Th. 10531 (US

Kittitas: slopes above Hyas Lake, 37 mi. NW. of Cle Elum, 3600', 7/14/42, Hitchc. 8045 (SU
rocky crest of ridge at mouth of Beverly Creek, 4500', 7/13/32, Thompson 8730 (SU,US
open rock slides at hd. Beverly Ck, 5000', 9/2/33, Thomps. 10042 (US toward incurva
open crests of ridges, Mt. Stuart region, 6000', 7/27/31, Thompson 7800 (SU
**Teanaway-Turnpike Basin Trail, Wenatchee Mts, 6800', 8/8/50, Kruckeberg 261 (RM**
Yakima: alpine slopes of Mt. Aix, Snoqualmie NF, 7000', 7/15/40, Th. 14995 (C,CAS,SU,US

Whatcom: Bagley Lake, Mt. Baker region, 4500', 7/25/30, Thompson 5381 (SU

Snohomish: mtn. mdws. of Mt. Dickerman, 4500', 7/17/32, Thompson 8873 (US

Pierce: nr. timberline, Paradise Val, Mt. Rainier NP, 7/24/26, Abrams 11493 (SU
rocky soil, Canyon Bridge, Mt. Rainier NP, 2900', 7/3/32, Warren 1763 (US
rocks nr. Reflection Lake, do., 7/18/19, Flett (US
Mt. Rainier, 5500', 7/8/19, Flett (US toward incurva
Mt. Rainier, 6500', 8/17/95, Allen 182 (C
**in stones, margin Nisqually River by Paradise trail, Rainier N.F.,** 7/7-11/08, Chase 4967 (US with inflor. of scabrella

Skamania: near snow line, Mt. Adams, 8/10/82, Henderson (US

(i) Mt. Paddo (Adams), loose soil of volcanic ashes, 8/10/82, Suksdorf s.n. (F,US labelled duplicate type of P. gracillima Vasey
Do., ca. 2000 m, 8/29/12, Suksdorf in Kneucker, Gram. Exsic.910 (US toward incurva
Do., 1882, Suksdorf (NY possible isotype
" " rocky places, 6-7000', 9/83, Suksdorf (F

Yakima: Mt. Paddo, E. side, 2000 m, 8/5/06, Suksdorf 5835 (US

wet banks, Mt. Adams, 1882, T. Howell 86 (US toward incurva
Mt. Paddo, base of cliffs on E. side, 6/16/10, Suksdorf 7081 (US
**do., 2200 m, 9/18/03, Suksdorf 2835 (F,SU,US cold, mossy springs**

Clallam: Mt. Angeles, Olympic Mts, 8/2/30, 5500', Thompson 5489 (SU & US = incurva

Do., do., 5500', moist slopes, 8/10/31, Thompson 7842 (SU,US & C = stenantha

Do., 5000', 7/18/31, Th. 7527 (SU toward incurva = incurva at C

Olympic Mts, 8/00, Elmer 1926 (SU, Minn), 1926 [NY = stenantha,] (SU

WASHINGTON, cont.

Mason: Olympic Mts, 8/90, Piper 973 (US do., 8/10/90, Piper 983 (US toward stenantha for latter coll. only

Jefferson: crevices of cliffs nr. Marmot Pass, 6500', 8/13/31, Thompson 8001 (US

Grays Harbor: talus slopes, trail to Col. Bob Lookout, 4700', 7/9/31, Th. 7314 (US

OREGON

Wallowa: Eagle Cap Peak, Wallowa Mts, 8000', 9/24/38, Sharsmith 3889 (C,US

Lostine Canyon 19 mi. above Lostine, Wallowa Mts, 7/19/33, Peck 17755 (SU

head Trout Creek, Wallowa Mts, 8000', 9/1/99, Cusick 2326 (C

left fork of Wallowa R, Wallowa Mts, 6100', 7/12/97, Sheldon 8530 (US, RM

extreme source of Wallowa R, 3000 m, 8/16/08, Cusick 3321c (CI,SU,US,RM
do. of Kettle Ck, Wallowa Mts, 7000', 8/20/07, Cusick 3202 (RM
Wallowa Mts. nr. the lake, 6000', 8/5/99, Cusick 2311 (C
high Wallowa Mtn. peak, 10,000', 8/13/06, Cusick 3139c (US
Wallowa Mts, 9000', 8/28/00, Cusick 2485 (C,F,RM

Baker: Eagle Creek Mts, dry mtn. sides, 6-8000', 1882, Cusick 877 (US

rocky slopes of Wallowa Mts. nr. Cornucopia, 7/20/36, Thompson 13378 (CAS

Powder River Mts, rock crevices, 9000', 8/96, Piper 2470 (US

Umatilla: ledges of basaltic cliff, S. canyon of Birch Creek, W. slope Blue Mts, 1000 m, 6/18/08, Cusick 3151 (C,SU,US

Hood River: alpine mdws, Eden Park, Mt. Hood, 5000', 8/7/27, English 836 (US

Josephine: Upper Biglow Lake, 2 mi. E. of Oregon Caves, 8/16/37, Applegate 11428 (SU,US as likely an odd form of scabrella or the Coast Range form of Canbyi with open panicle

CALIFORNIA

Siskiyou: Spirit Lake, 6000', 8/2/39, Howell 14912 (CAS,SU

Trinity: hd. of White's Ck, Devil's Canyon Mts, 6800', 8/7/35, Tracy 14594 (C,US
Do., do., do., 8/5/35, T. 14549 (C,GH

Butte: Appleton, 7/16/16, Hazeltine (C

Nevada: above Donner L, 7/4/92, Sonne 17 (C cited in descrip. of P. invaginata Scribn. & Williams.

Mt. Stanford, 8700', 8/88, Sonne 24 (US " " " " " "

CALIFORNIA, cont.

Nevada or Placer (?): Summit Camp, Sierra Nevada, 7/10/70, ? (US, in part, type of P. invaginata Scribn. & Williams. Two specimens of gracillima and one of incurva, the description a composite ("panicle subpyramidal to oblong"), but based principally on the gracillima specimens.

Placer: Donner Pass to Mt. Lincoln, 7/21/43, Howell 18638 (CAS

Eldorado: Mt. Tallac, rocky soil, 7000-9500', 8/6-8/08, Hitchcock 3134 (US
Do., do., H. 3139 (US
nr. Echo Camp, above Echo Lake, 7000', 8/6/15, Heller 12146 (CAS,SU), 12148 (CAS,SU,US

Lily Lake, Lake Tahoe region, 7/25/07, Pendleton & Reed 1244 (C

Alpine: near Ebbett's Pass, 8/7/63, Brewer 2077 (C
below Ebbett's Pass valley floor, 7500', 7/29/42, Beetle 3795 (RM

Mono: Slate Ck. basin, 3080 m, 8/19/36, Keck 4432 (CI,SU grass-covered rocky slope

Ellery Lake, 9500', 8/22/36, Yates 6289 (C

Tuolumne: Dardanelles, 2 mi. SW. Sonora Pass, 8700', 8/20/36, Yates 6239 (C,SU

Emigrant Mdw, 9400', 7/26/36, Peterson 379 (C

Slide Canyon, W. of Finger Pk, N. bound. YNP, 10500', 7/30/34, Bartholomew (C

Slide Mtn, 9600', do., 7/29/34, Bartholomew (C

betw. Benson L, and Smedberg L, ca. 8500', 7/26/34, Bartholomew (C
nr. Tioga Pass, 9800', 8/22/36, Yates 6317 (C

vic. Tuolumne Mdws, 8500-9500', 7/02, Hall & Babcock 3547 (C
nr. Parson's Lodge, Tuol. Mdws, 8/5/44, Howell 19964 (CAS
nr. Lembert Dome, Tuol. Mdws, 8600', 8/8/33, Sharsmith 314 (C,SU

upper end Lyell Canyon, 9500', 7/25/33, Sharsmith 183A (C

Mariposa: high gap S. of Elizabeth L, 8/8/44, Howell 20150 (CAS

Lyell Fork, Merced R, 8/1/35, Schreiber 1920 (C

Lake Tenaya, 8300', 6/02, Hall & Babcock 3503 (C,US

summit Cloud's Rest, 7/12/89, Chestnut & Drew (C

Madera: Garnet Lake, 9700', 8/5/41, Howell 16774 (C,CAS 7/24/41, 16425 (CAS

Soda Springs, Upper San Joaquin, 8/20/95, Congdon (US

Rush Creek Divide, E. of Minarets, 8/22/99, Congdon (US

Inyo: top of Escarpment at Heart L, Rock Ck. L. Basin, 10,700', 8/19/33, Peirson 10826 (US
do, 8/6/40, Peirson 12939 (CAS

CALIFORNIA, cont.

Inyo, cont.

Bishop Creek, 7/1911, A. Davidson 2512 (US
Bottleneck Lake, Bishop Ck. region, 11,400', 7/17/50, Bracelin 2939 (CAS,NY

Mosquito Flat, Rock Creek Lake Basin, 10,300', 7/14/46, Howell 22358 (CAS
near Rock Creek Lodge, 8000', 8/6/32, Halperin 534 (CAS

Fifth Lake, Big Pine Lakes, 11,000', / / , Howell 23782 (CAS

hd. Lone Pine Canyon, E. side Mt. Muir, 12,200', 8/20/37, Sharsmith 3307 (C,SU

Mt. Whitney Portals, 8500', 7/16/39, Kerr (CAS

Army Pass, 12,000', 8/5/49, Howell 26054 (CAS with rhizomes due to burial

Fresno: Mono Rock, 10500-11000', 7/20/46, Howell 22556 (CAS toward incurva

jct. N. Goddard Creek and S. Fk. San Joaquin R, 10,300', 8/5/35, Ferris 9306 (SU

dry ground in rock crevices, nr. Round Mdw, Kaiser Ridge above Huntington L, 7/22/27, Swallen 836 (US

Bubbs Creek nr. Vidette Mdws, 9500-10,000', 7/23/48, Howell 24929 (CAS

Tulare: vic. Mt. Whitney, 12,000', 8/7/97, Dudley 2486 (SU

vic. Alta Peak, Sequoia NP, 6000-8000', 8/21/96, Dudley 1708 (SU

Rockslide Lake, Kaweah Pks, 10,000', 8/4/97, Dudley 2389 (SU

reg. Mineral Gap: trail Bullion Flat to Mineral King, 10,589', 8/11/97, Dudley 2562 (SU

Farewell Gap, 9500', 8/30-9/9/08, Hitchcock 3384 (US
S.E. of Farewell Gap, 10,800', 7/20/51, Howell 28019 (CAS,NY
Ouzel Basin, 10,500', 8/4/40, Howell 15986 (CAS

betw. Big Arroyo and Chagoopa Plateau, 8/5/42, Howell 17719 (C,CAS

Little Five Lakes Basin, 7/29/42, Howell 17412 (CAS

reg. Tule-Little Kern Divide, Hockett Mdw, 8-9000', 8/1/95, Dudley 1038, 1053 (SU

Eagle Lake Trail nr. Mineral King, 7/25/42, Howell 17189 (CAS
White Chief Trail nr. Mineral King, 9500', 7/17/51, Howell 27856 (CAS,NY
Sky Blue Lake, 11,500', 8/3/49, Howell 25982 (CAS

Poa incurva Scribn. & Williams (P. saxatilis Scribn. & Williams)

Alberta: Waterton Lakes N.P., summit of Mt. Crandell, alpine zone, 8/9/53, Breitung 17373

Do., at timberline on Mt. Crandell, 7600', do, do. 17362

CANADA

BRITISH COLUMBIA

Kootenay: Roger's Pass, summit of Selkirk Mts, 4500', 7/31/90, Macoun 83b (US in part

MONTANA

Judith Basin (?): Little Belt Mts, near the Pass, 7000', 8/10/96, Flodman 129 (NY

Madison: Spanish Basin, Madison Range, 6000', 7/11/96, Flodman 128 (NY

WYOMING

Johnson: dry hillsides nr. Cutler Creek, T56N, R88W, 8000', 7/25/36, LO & RWilliams 3110 (NY toward Canbyi but quite good incurva

Teton: Teton Mts, 8/16/99, Nelson 6530 (C,NY,RM
Black Rock Creek, Teton Forest Reserve, 10,000', 8/97, Tweedy 6 (NY !

**Sublette: Gros Ventre Mts, nr summit, 15 mi. ne. Bondurant, 8/15/22, EB & LB Payson 3050 (RM**

NEVADA

Lander: Toyabe Mts, 10,000', 7/29/13, Kennedy 4171 (SU
Bunker Hill slopes, 10,000', 7/29/13, Kennedy 4160 (CAS
DO., 6/29/31, JM & MAR Linsdale 518 (CAS

Mineral: summit Mt. Grant, Wassuk Range, 11,300', 6/26/38, Archer 6793 (SU

Clark: Charleston Peak, open ridge, 3450 m, 7/17/36, Clokey 7032 (CAS,NY

IDAHO

Fremont: Mt. Chauvet, 10,000', 7/29/97, Rydberg & Bessey 3658, 3659 (NY

Bonner: Queen Mt, Pend Oreille Forest, 1700-1800 m, 8/6/13, Eggleston 9786 (US

Custer: W. base of Mt. Hogg, Lemhi Range, talus in cirque, 10,000', 8/19/44, Hitchcock & Muhlick 11254 (CAS,GH, NY,SU,RM slightly open, det. as gracillima

Parker Mt, exposed rocky summit, 8500', 7/17/16, Macbride & Payson 3242 (C,CAS,NY,SU

limestone ridge, Lost River Mts, Leatherman Pass, hd. W. Fk. Pahsimeroi **River, 11,000', 8/16/44, Hitchc. & Muhlick 11162 (CAS**

**Mahogany Ck, Lost R. Mts, nr. Mt. Borah, 8/12/44, H & M 11060 (SU CAS** Canbyi??

E. Fk. Pahsimeroi River, do, 10,300', 8/14/44, Hitchc. & Muhlick 11098 (CAS,GH,NY,SU,RM

Mackay (Bear Canyon), slide rock, 6700', 7/31/11, Nelson & Macbride 1455 (SU

Poa incurva Scribn. & Williams (P. saxatilis Scribn. & Williams) - page two

IDAHO, cont.

Valley: granite slopes of Gold Fork Lookout, Sawtooth Mts, 8100', 7/9/37, Thompson 13771 (CAS

Blaine: talus N. side upper Norton Lake, vic. Norton Pk, Smoky Mts, 9000', 8/3/44, Hitchc. & Muhlick 10699 (SU 31 cm. high
ridge above W. side of Alturas Lake, 8200', 6/22/41, Cronquist 2624 (GH

Elmore: talus N. side Bald Mt, 9200', 7/23/44, Hitchc. & Muhlick 10242 (CAS, NY, SU, RM, UW

rocks above Middle Spangle Lake, Sawtooth Primitive Area, headwaters Middle Fk. Boise River above Atlanta, 7/19/44, Hitchc. & Muhlick 10151 (CAS, NY

WASHINGTON

Walla Walla: high ridges of the Blue Mts, 7/15/96, Piper 2555 (US

Okanogan: Sheep Mt, Okanogan (now Chelan) Forest, 2100 m, 7/30/16, Eggleston 13304 (US

Muckamuck Lookout, exposed open summit of, 6300', 6/26/31, Thompson 7006 (C, SU
do, do, 6390', 7/21/31, Robt. Bigelow 129 (SU

Whatcom: steep S. slope Mt. Hermann, Mt. Baker region, [illegible]', 8/14/30, Thompson 5726 (SU
Church Mountain, 7/5/37, Muenscher 9008 (C
alpine slate ledges, Grouse Ridge, Mt. Baker, 6700', 6/8/[illegible], St. John 5109 (US

Chelan: Chumstick Mt, 5700', 6/23/32, Thompson 8521½ (SU

Kittitas: Bald Mt, Snoqualmie NF, 5800', 6/22/40, Thompson 14801 (C, CAS, SU

alpine slopes of Table Mt, Wenatchee Mts, 6200', 7/1/40, Thompson 14916 (C, CAS

Pierce: Mt. Rainier, 5500', 7/8/19, Flett (US toward gracillima
river bars kept moist by spray from falls Ohanapecosh, Mt. Rainier, 2100', 7/29/19, Flett (US toward gracillima
Terminal Moraine, Flett's Glacier, 8/27/19, Flett (US
dry rocky places, Mt. Rainier, 7000', August 1895, Piper 1964 (US isotype of P. saxatilis Scribn. & Will. panicle rather strict but more open than many incurva. Somewhat intermediate to gracillima.

Yakima: S. E. slope Mt. Paddo, 10/5/02, Suksdorf 5389 (US

Clallam: Mt. Angeles, 5000', 7/18/31, Thompson 7531 (C, SU moist alpine mdws.
do, do, 7527 (C
dry summit of Mt. Angeles, 5500', 8/2/30, Thompson 5489 (US at SU = gracillima

Jefferson (?): moraine of Duckabush ("Duckaloose") Glacier, Olympic Mts, 7000! August 1[illegible], C. V. Piper 1989 (US type of incurva a slender alpine
open alpine ridges in Marmot Pass, Olympic Mts, 6000', 8/18/33, Thompson 9953 (US

OREGON

Wallowa: nr. Mirror Lake, 7/20/33, Peck 17792 (NY,SU

Jackson Lake, 7/16/33, Peck 17685 (NY,SU,VW

nr. summit source Imnaha and Wallowa rivers, 2930 m, 8/29/07, Cusick 3226 (C,CI,SU

source Kettle Ck, main divide Wallowa Mts, 2330 m, 9/19/07, Cusick 3205 (C

Baker: Wallowa Mts. nr. Cornucopia, 7/20/36, Thompson 13376 (CAS

Sumpter Valley watershed, Blue Mts, 7600', 7/8/19, Ferris & Duthie 940 (SU

Hood River: N. side Mt. Hood, 6000', 8/1/86, T. Howell 202 (US
do, 8/1/86, THowell (NY

Bluegrass Ridge, Mt. Hood, 5500', 7/22/28, Thompson 5095 (SU ? Clackamas Co.

Clackamas (?): Eliot Glacier, 6000', 7/23/28, Thompson 5047 (SU,US← This sheet was the basis for the report of Pattersonii on Mt. Hood.

Grant: top Dixie Mt, 7800', 7/25/25, Henderson 5613 in part (CAS with Cusickii

Harney: Fish Lake, Steens Mts, 7000', 7/15/35, Thompson 12093½ (NY,SU 12095 (NY,SU,VW

top of Steens Mts. above Alberson, 2800 m, 7/4/25, Peck 14244 (F

Jackson: Red Mts, Siskiyou Mts, 7500', 7/29/35, Thompson 12386 (C,NY

Josephine: Bolan Lake, Siskiyou Mts, nr. Calif. line, 6000', 7/7/39, Hitchcock & Martin 5242 (C,SU

CALIFORNIA

Siskiyou: Marble Mt, 9000', 6/01, Chandler 1664 (C

Lassen (Plumas??): dry hard soil, summit of mts. 5(?) mi. S. of Susanville, 7/27/27, Swallen 865 (US

Sierra: Webber Lake, dry mtn. slope, 8/4/00, Leiberg 5256 (US

Nevada: Bierstadt Peak, Truckee R. basin, 6/25-30/97, Davy 3216 (C 7500' 3213 (US

Castle Peak, 7/18/43, Howell 18549 (CAS,NY

nr. Donner Lake, open sandy woods, 7/14-16/13, Hitchcock 10487 (US
**Boreal Ridge, Donner Pass, nr. Norden, 7000', 7/16/43, Howell 18397 (CAS**

Placer: nr. Donner Pass, 7800', 8/13/17, Heller 12917 (CAS,SU
Donner Summit, 7200', 6/1/28, Smith 2277 (C

betw. Truckee and Tahoe, 6/25-30/97, Davy 3265 (C

Poa incurva Scribn. & Williams (P. saxatilis Scribn. & Williams) - page four

CALIFORNIA, cont.

Placer, cont.

flat summit Mt. Lincoln, 8000', forming large fairy rings, open dry ground, 7/14-16/13, Hitchcock 10511 (US do, rocky slope, H. 10512 (US
do, do, 7500', H. 10513 (US
do., summit, H. 10507 (US , 10504 (US

Eldorado: Mt. Tallac, 9740', 7/03, Hall & Chandler 4029 (C,US
do, 8/31/46, Howell 22947 (CAS toward gracillima
do, rocky hill in open ground, 7000-9500', 8/6-8/08, Hitchcock 3126
summit of Mts. above Lake Tahoe, 8/2/27, Swallen 921 (US ? Co.

Desolation Valley, 8500', 8/30/42, Stebbins 3324 (C 3312 (C

E. slope Freel Peak, 10,600', on Alpine Co. line, 8/30/37, Sharsmith 3487(C

Jefferson: open alpine ridges in Marmot Pass, Olympics, 6000', 8/18/33, Thompson 9953 (US

Alpine: E. side East Blue Lake, decomposed granite, 8100', 8/12/33, Wolf 5218 (CI
2 mi. W. of Sonora Pass summit, 7/2/36, Hoover 1467 (C

Mono: 2.5 mi. nw. Belfort, 11,300', 7/16/37, Graham 50 (C

3/4 mi. E. Tamarack Lake, 10,700', 7/19/37, Hendrix 396 (C
S. side Tamarack Lake, 9700', 7/19/37, Hendrix (C

Slate Creek, mdw. at T. L., 10,000', 8/26/37, Keck 4597 (CI
Do, 10,200', 7/19/35, Keck & Clausen 3870 (C,CI
ravine at hd. Slate Ck, 10,800', 8/27/37, Keck 4639 (C,CAS,CI
Middle Ridge, Slate Creek Basin, 10,200', 8/26/37, Keck 4583 (CI

saddle betw. Mt. Conness and East Plateau, 11,000', 8/27/37, Keck 4636 (CI,SU
ridge E. from Mt. Conness, 11,200', dry ledges, 8/27/37, Keck 4620 (CI

North Mt, talus, 10,650', 6/23/35, Clausen 1154 (CI,SU

White Mt, granite gravel, 11,500', 8/17/36, Keck 4387 (CI

Tioga Crest, E. side Saddlebag L, 11,400', 7/25/37, Sharsmith 2753 (C,SU

Dana Plateau, 12,200', 7/15/37, Sharsmith 2494A (C

Tuolumne: Emigrant Mdws, S. 10, T4N, R21E, 9400', 7/25/36, Peterson 379 (SU

**Burro Pass nr. Matterhorn Pk, 11,000', YNP, 7/31/34, Bartholomew (C**

**northwest plateau of Mt. Dana, 11,800', 9/18/36, Sharsmith 2344 (C**
this I label "Possibly Poa incurva x P. Suksdorfii" It is a very dense leafy tuft, even denser than Suksdorfii, and has puber. lemmas like incurva. It may be a n. sp.

N. ridge Mt. Gibbs, 12,000', 7/17/37, Sharsmith 2567B (C

GAylor Lakes, 10,800', 8/12/44, Howell 20420 (CAS
S. slope Kuna Peak, 12,500', 7/21/37, Sharsmith 2700 (C

CALIFORNIA, cont.

Tuolumne, cont.

betw. Dog L. and Tuolumne Mdws, 8/6/44, Howell 20011 (CAS
summit Lembert Dome, 9300', 7/13/33, Sharsmith 37 (C
slope N. of Tuolumne Mdws, 8/6/44, Howell 20016 (CAS 8600', 7/11/33, Sharsmith 20C (C,CAS

betw. Cathedral Peak and Echo Peak, 10,000', 7/14/33, Sharsmith 73 (C

Mt. Lewis (n. Parker Pass), 11,300', YNP, 7/22/37, Sharsmith 2732 (C

Mariposa: Lyell Circque, 7/25/35, Schreiber 1799, 1958c, 1970 (C

high gap S. of Elizabeth Lake, 8/8/44, Howell 20152 (CAS

Merced Lake, 7200', 7/9/09, Jepson 3190 (US

summit Sentinel Dome, 7/21/15, Hitchcock 13140 (US

Madera: Agnew Pass Trail, 7/31/41, Howell 16570 (CAS

Garnet Ck. to San Joaquin Mt, 8/8/41, Howell 16839 (CAS
nr. Garnet Lake, 9700', 7/30/41, Howell 16524 (CAS 8/5/41, 16773 (CAS, pars

Fresno: Graveyard Mdw. nr. Silver Pass, 8500', Grant 1540 (SU
Woods Creek, 7/19/10 (Clemens (RM
n. of Mono Pass, 11,500', 8/8/37, Sharsmith 3022B (C

s. slope Kaiser Crest nr. Huntington L, 9500', 7/11/26, Klyver (SU
dry hills above Huntington L, 7/23/27, Swallen 842 (US 844 (US

Pioneer Basin, 10,800', 7/22/46, Howell 22647 (CAS
Vermilion Valley, 7200', 1926, Klyver (SU

Humphreys Basin, w. Mt. Humphreys, 11,500', 8/12/37, Sharsmith 3167 (C,SU

Martha Lake, headwaters S. Fk. San Joaquin R, 10,750', 8/5/35, Ferris & Lorraine 9169, 9213 (SU

Mt. Gould, n. of Kearsarge Pass, 12,500', 8/15/37, Sharsmith 3200 (C

Bubbs Ck. nr. Vidette Mdws, 9500-10,000', 7/23/48, Howell 24933 (CAS

Inyo: Mosquite Flat, Rock Creek L. Basin, 10300', 7/14/46, Howell 22357 (CAS,SU
Do, do, 7/15/46, H. 22364 (CAS

slope Mt. Mills, nr. Mono Pass, hd. Rock Ck, 12,800', 8/6/37, Sharsm. 3053 (C
Heart Lake Mdw, Rock Ck. Lake Basin, 10,500', 8/5/33, Peirson 10827 (C
reg. of Heart Lake, do, 10550', 7/23/34, Peirson 11312 (US
Piute Pass, 10,900-11,100', 7/22/34, Ferris 8925 (SU

hd. Lone Pine Canyon, E. Mt. Muir, 12,200', 8/20/37, Sharsmith 3303 (C
defile across Transverse Ridge, Rock Ck. Lake Basin, 10,700', 8/19/40, Peirson 12934 (CAS

Fifth Lake, Big Pine Lakes, 11,000', 8/15/47, Howell 24120 (CAS
Third to Fifth Lake, do, 10,500', [illegible]/47, H. 23905 (CAS

CALIFORNIA, cont.

Inyo, cont. crest of Inyo Mts., 3 mi. e. of Badger ~~Pass~~ Flat, 10,850', 8/13/53, JC & AR Roos 6018 (NY
S. end of Coyote Ridge, 12,000', 8/11/47, H. 24033 (CAS
Coyote Ridge (E. of So. Fork of Bishop Creek), 11,600', 8/7/50, Raven & Stebbins 224 (CAS, NY
ridge above Dingleberry Lake, Bishop Creek region, 10,700', 7/16/50, Bracelin 2911 (CAS, NY

nr. Blue Lake, above Lake Sabrina, 10,100', 8/9/50, Raven & Stebbins 260 (CAS, NY

ridge betw. Flower and Bench lakes, E. of Kearsarge Pass, 7/19/48, Howell 24788 (CAS

edge of Lone Pine Lake, Mt. Whitney trail, 7/8/39, Kerr 402 (CAS

Tulare: betw. Reflection lake and Harrison Pass, 8/8/40, Howell 16040 (CAS ~~2911, CAS, NY~~

Center Basin, 12,000', 7/28/48, Howell 25101 (CAS

w. side Kaweah Peaks Ridge, Nine Lakes Basin, 12,000', 8/21/38, Sharsmith 3810 (C

Alta Mdws, 10,000', 7/02, Grant (SU

Little Five Lakes Basin, 7/29/42, Howell 17[illegible]13 (CAS

rocky shores of Long Lake, do, 10,500', 7/29/43, Ferris & Lorraine 10695 (SU

mdw. Head of Whitney Creek, Mt. Whitney, 11,500', 8/30-9/9/08, Hitchcock 3449 (US
some characters of Cusickii, lemma ± keeled, nearly smooth (only scabrid), and large (to 5.5 mm. long); ligule long
rocky woods, along Whitney Creek, Crabtree Mdws. to Mt. Whitney, 8/30-9/9/08, Hitchcock 3437 (US
rocky woods, hd. E. Fk. Kern Canyon to Crabtree Mdws, 10,500', do. H. 3427 (US

gorge N. of Bakeoven Mdws, 8200', S. Fk. of Kern R, 7/16/50. Howell 26981 (CAS, NY

Upper Bear Creek Canyon, Sequoia NP, 8/28/00, Dudley 2895 (SU

betw. Big Arroyo and Chagoopa Plateau, 8/5/42, Howell 17718 (CAS

Soldier Lake, 11,000', 7/23/49, Howell 25647 (CAS
Columbine Lake, 8/7/42, Howell 17811 (CAS - immature
Rock Creek, 10,800', 8/3/49, Howell 25969 (CAS ca. 10,000', 8/7/49, H. 26125 (CAS

Farewell Gap, 10,500', 7/20/42, Ferris & Lorraine 10704 (C, SU
Do, do, 1897, Purpus 5207 (US do, 10,000', 8/30-9/9/08, Hitchc. 3383 (US

White Chief nr. Mineral King, 9100', 7/15/42, Ferris & Lorraine 10607A (SU
White Chief Trail, nr. Mineral King, 9500', 7/17/51, Howell 27857 (CAS, NY
Black Rock Pass, 11,500', Sequoia NP, 7/30/43, Ferris & Lorraine 10911 (C, SU

Poa incurva Scribn. & Williams (P. saxatilis Scribn. & Williams) - page seven

CALIFORNIA, cont.

Ventura: summit Mt. Pinos, 8800', 7/7/05, Hall 6546 (C a form of this? culms too leafy

San Bernardino: Mt. San Gorgonio, 11,500', 7/25/47, Howell 23680 (CAS lemmas scabrid but scarcely at all puberulent.

Ditto, summit, 11,400', 7/12/08, Abrams & McGregor 758 (SU

Poa juncifolia Scribn.

SASKATCHEWAN. dry open hilltop, Cypress Hills, 6/25/36, J.L.Bolton (US much more slender than the ampla from here subsp. Porteri?

BRITISH COLUMBIA

Yale: open dry woods on high ground, Arid Transition, Monte Creek, Kamloops, 6/12/40, Eastham 7262 (US very narrow-lvd. ampla type

Allies Mine, Tranquille, 4000', 7/23/35, ex Dominion Range Exp. Sta., Kamloops (US
alpine slopes of Bulster Mt, Marble Mts, 7000', 7/14/38, JW & EM Thompson 431 (US, NY toward ampl

alkali marsh, Similkameen R, 6/9/05, Macoun 72971 (US, NY

MONTANA ? Co.: Deer Lodge Valley, 5000', 7/25/05, Jones 11394 (SU
Powell: sagebrush knolls ca. 6 mi. N.E. of Helmville, 6/28/45, Hitchc. & Muhlick 11688 (UW
Granite: open grassland, moist mdw, Smart Cr. Basin, Deerlodge Forest, 5500', June 1936, DRDrumheller (Forest Service #79210) (US distrib. as epilis

Gallatin: Bozeman, RR track nr. depot, 6/14/46, Booth 1088 (US

Billings, 7/00, Griffiths & Lange 1 (NY

Silver Bow: Melrose, 1895, Rydberg 2096 (SU

WYOMING

Sheridan: moist mdws. 1 mi. S. of Sheridan, 7/6/35, Williams 2368 (US dry subalpine mdw, Owen Ck, Big Horn N.F, 8500', 7/23/36, Porter 2202 (US, RM

Sheridan (?): foothills betw. Sheridan & Buffalo, 6000', 6/15-7/15/00, Tweedy 3692 (NY, RM

Park: 3 mi. N. of Meeteetse on dry hillside, 6/22/47, Beetle 4588 (C keel puberulent! ~~NY~~

Teton: in clump of sagebrush, E. of Moran, 7/29/26, Hitchcock 23189 (US
glaucous; open aspen woods on steep slope, Jackson, 6200', 7/22/26, H. 23116 (US
Buffalo Fork, 7000', 7/97, Tweedy 19 (NY Teton For. Res.
Albany; dry sandy roadside, nr. Big Hollow, 15 mi. W. Laramie, 7500', 8/6/44, Porter 3475 (CI, RM

Carbon: Battle, 10-11,000', 7/25/01, Tweedy 4346 (NY
Grand Encampment Creek, 8/13/97, ANelson 3999 (RM
Sweetwater: Black Rock Springs (= Rock Springs) ~~(Point of Rocks?)~~, 7/13/97, A. Nelson 3721 (US, NY, RM isotypes "along a dry run" 2 isotypes NY. One reads: "No. 3721. Awen Nelson. July 13, 1897. Point of Rocks, Sweetwater Co., Wyo. Black Rock Springs, 6800. One of the most desireable pasture grasses of the locality."
10 mi. N. of Point of Rocks, 6/20/01, Merrill & Wilcox 34 (C, RM 32 (C, RM = nevad. form toward ampla
in bunches in alkaline soil nr. "Boars Tusk", 6/30/01, Merr.& Wilcox 80 (NY
Point of Rocks, South Butte, in small marsh mdw, 6900', 7/13/97, Nels. 3751 (US
Granger, Hams Fork, 7/30/97, ANelson 3891 (RM
Co.?: Uinta Mts, Wyoming Terr., 8/72, Dr. Jos. Leidy (NY
Uinta: Evanston, 7/27/97, ANelson 3837 (RM 7/28/97, ANelson 3869 (RM

Sweetwater: South Butte, 7/13/97, ANelson 3751 (RM
Beaver Basin, Colorado line, 7/22/97, ANelson 3807 (RM with Porteri
in loose bunches in alkali bottom nr. Leucite Hills, 7/2/01, Merrill & Wilcox 94 (RM 4 dm high, brd. lvs. of ampla but narrow inflor. of juncif.
[illegible]

**Poa** juncifolia Scribn - page two

COLOR-DO

Moffat: 1/2 mi. W. of Lay, 5/31/40, Pohl 1960 (US
**Grand: Coulter P.O., 7/12/05, Osterhout 3009 (RM**
Grand: Hot Sulphur Springs, Middle Park, mdws on Grand R, 8/1/81, Engelmann (NY
**Sulphur Springs, 7/16/05, Osterhout 3064 (RM toward ampla**, 7/20/07, Clements (NY
Gunnison: reg. of Gunnison Watershed, Iola, 7480', 7/29/01, Baker 656 (C,NY ssp. gracilenta NY, type! RM

IDAHO

Lemhi: open sagebrush desert 6 mi. E. Gilmore, 6/25/44, Hitchc. & Muhlick 9295 (SU

Oneida: Holbrook-American Fall. Hiway, Commons' Ranch, sagebrush, 5600', 7/23/40, Hull 244 (NY

Custer (or Butte): Lost River Mts, 10,200', 8/14/95, Henderson 3940 (CAS,US

Blaine: Clyde, dry saline flat, 5600', 7/11/16, Macbride & Payson 3182 (CAS,CI,SU,NY?US
**Cassia: Shoshone R.S, damp soil, aspen type, 7/30/38, Gierisch 795 (NY**
← Canyon: Caldwell, base of RR. bank nr. tule marsh, 2400', 7/18/08, Chase 4726 (US

UTAH **Edward's Creek, Lookout Mts, 7/3/59, Henry Engelmann (NY**

Co.?: Edward's Creek, Lookout Mtn, 7/3/59, Engelmann (NY lig. 2.4 mm. long

Wasatch: E. side Strawberry Valley, sandy slope, artemisia assoc, 7/1/38, Harrison & Nisson 8760, 8762 (C

Summit: Kamas, 6850', 7/7-11/00, Pammel & Stanton (US

Cache: saline soil, mdws, 3 mi. W. of Logan, 7/2/36, Maguire 13874 (CAS, RM

Co.?: sagebrush plain nr. Mill Ck. R.S., 9000', 7/18/26, Hitchcock 23105 (US do, aspen woods, 7/15/26, H. 23091 (US
**Tooele: Lake Point, 4200', 7/19/79, Jones 1021 (NY, 1 sheet some Canbyi** under this number

NEVADA

Elko: Lone Mtn, 7500', 8/5/13, Kennedy 4320 (CAS

Nye: Mohawk R.S., 8000', 6/17/31, JM & MAR Linsdale 214 (CAS with ligule 2.5 mm. long

Washoe: Hines Ranch, foot of Peavine Mt, 7/1/07, Heller & Kennedy 8661 (CAS?CI?SU

Wadsworth, 7/21/87, Tracy & Evans (SU
nr. Steamboat, sandy alkaline soil, full sun, roadside , 5/26/41, Beetle 2700 (CAS
Reno, 7/10/80, Tracy (SU
**Hunters Creek road, 8 mi. SW. Reno, mt. mdw., dominant plant, 6500', 7/14/38, Archer 6345 (SU?NY**
nr. Washoe Hill ca. 17-19 mi. S. Reno on rd. to Carson City, 6/19/37, Miller 112 (NY,SU

Ormsby: 1 mi. N. Carson City, 6/2/37, sandy loam, Lehenbauer 1 (SU ligs. rather long, but otherwise juncif. rather than nevadensis
King's Canyon, 1700-2000 m, 7/14/02, Baker 1314 (NY 1311 (NY

WASHINGTON

Co.?: Coleville Reservation, 6/02, Griffiths & Cotton 396 (US

Okanogan: betw. Tonasket and Republic, low damp mdw, 6/29/31, Thompson 7122 (C,SU
Do., moist ground, do,, T. 7138 (C,SU
nr. Tonasket, 2000', alkali soil, 7/2/32, Thompson 8692 (C,NY,SU toward ampla

Brewster, 6/15/02, Griffiths & Cotton 264 (US

Lincoln: nr. Almira, sagebrush slopes, 6/16/~~93, Vasey 3010 (US~~ Thompson 11667 (NY, SU
Whitman: Steptoe ("Streptoe"), 6/15/01, Vasey 3010 (US
Wawawai, 5/31/03, Piper 4127 x (US

Adams: Cow Creek, edge of moister areas, 6/28/02, Griffiths & Cotton 512 (C

Grant: (labelled Douglas Col), 1300', 6/22/93, Sandberg & Leiberg 267 (C,NY,US,RM isoptypes of P. brachyglossa; another isotype in US is labelled "Low grassy land at junction of Crab and Wilson Creeks. June 22. Alt. 1300 ft. 267." No other data but on a label "Herbarium of A.S. Hitchcock" and in the Scribner Herb. purchased by A.S.H. Also US, type!

moist ground nr. Blue Lake in Grand Coulee, 6/16/35, Thompson 11631 (SU
Do., around margin of Blue Lake, moist ground, T. 11633 (US
Do., in alkali marsh by Blue Lake, T. 11667 (US

Coulee City, in low mdws, 6/1/02, Piper 3918 (US 3917 (US
moist spring below Dry Falls, Coulee City, 6/22/33, Thompson 9117 (NY

Benton: Prosser to Rattlesnake Hills, 6/02, Griffiths & Cotton 1 (SU toward ampla
Rattlesnake Mts., 16 mi. N. of Prosser, 6/16/01, Cotton 412 (US do., side hills of gulches, 6/15/01, C. 412 (US, RM

Yakima: Toppenish, alkaline mdws, 6/4/03, Cotton 1174 (US
Cold Creek, Yakima region, abundant in mdws, 6/1/01, Cotton 402 (RM, NY

Peirce: Steilacoom, 5/27/88, Piper (US fairly typical, lemmas slightly puberulent

OREGON "E. Ore.", 1100 m, 6/27/06, in small tufts, alkaline soil, Cusick 3049 (SU,F,US, RM
Do, 1200 m, 7/06, Cusick 3069 (C,CI,NY,SU, dry soil, RM

Wallowa: dry ground, Ice L, 7/16/34, Peck 18568 (CAS,US towards ampla

Wasco: 7 mi. SE. The Dalles, bunchgrass paririe, open hillsides, Arid Transition, 6/26/17, Lawrence 85 (SU
dry ground nr. Dalles City, 6/7/04, Suksdorf 638 (US

Union: La Grande, (2800'), 6/22/04, Hunter 579 (US
nr. Union, 6/05, Cusick 3055 (US ligules 2.5 mm. long and acuminate

Baker: Baker, 1000 m, 6/17-21/16, Eggleston 12624 (NY

Grant: Izee, 7/15/02, Griffiths & Hunter 207 (US

Crook: "desert", 6/10/02, Cusick 2809 (C,SU, NY
nr. Crooked R, at Prineville, 955 m, 6/22/94, Leiberg 309 (NY
Hay Creek, 840 m, crevides of rocks, 6/12/94, Leiberg 210 (C,US

nurly.Narrows, 7/02, Griffiths & Hunter 257 (NY

Malheur: Barren Valley, swaley flats, common, 6/12/01, Cusick 2562 (C,F,RM
nr. Beulah, 1080 m, 6/17/96, Leiberg 2305 (C,NY toward ampla; 6/18/96, 2316 (NY,US,RM

Harney: Silver Creek Valley, large tufts, abundant in moist or dry mdw, 6/27/01, Cusick 2614 (C,NY,F,RM distributed as Poa rubriflora Scribn. ined.
Steins Mtn, 6/1/85, THowell (NY
Silvies R. to Burns, 7/02, Griffiths & Hunter 217 (US towards ampla

Steens Mts. dry soil, 6/29/98, Cusick 1981 (C,RM

Lake: Goose Lake Valley nr. Lakeview, wet, slightly alkaline mdw, 6/28/27, Peck 15312 (SU

Klamath: nr. Recreation, W. side Upper Klamath L, 7/17/20, Peck 9520 (NY,SU

Beatty, summer 1930, Isabel T. Kelly 25 (CAS

CALIFORNIA

Siskiyou: 1 mi. SE. of Gazelle, 2600', 6/21/36, Yates 5823 (C toward ampla

Lassen: Squaw Valley, 6/27/35, Whitney 3317 (C toward ampla

Plumas: Red Clover Valley, 7/5/07, Kennedy & Heller 8751 (CAS

Chester, 6/15/18, McKee & Westover (US

Nevada: Donner Lake, 6/00, Dudley (SU do., meadow soil, 6/14/00, Dudley (SU

Placer: Snow Mt, Truckee Q, S19, T16N, R14E, 8000', 7/13/34, Bolt (Veg } prob. ok but ligule not seen; labelled brachyglossa

Alpine: 1 mi. W. of Carson Pass, 8300', 6/30/34, Yates (Veg

Tulare: Bokeoven Mdws, So. Fk. Kern R. 8100', 7/20/50, Howell 27127 (CAS,CI,NY
Do., around mineral springs, 7/18/50, Howell 27047 (CAS,CI,NY,SU
Do., mineral seepages in gorge N. of, 8200', 7/16/50, H. 26971 (CAS,NY

Siskiyou: 3 mi. S. of Grenada, Shasta Valley, dry subsaline adobe flat, 2600', 6/30/35, Wheeler 3635 (NY,M basal rosettes dense (no rhizomes), with crowded short glaucous involute coriaceous smooth filiform leaves 5 cm. long (4—7), the culms 30-35 cm. long and nearly naked, their blades only 1-3 cm. long; ligules 1.5 mm. long, obtuse; culms very slender and the too open panicle graceful and very floriferous; lemmas glabrous except for a few hairs at the callus. Not good fibbata. cm.

Tulare: Monache Mt., 8100-8500', 7/21/50, Howell 27160 (CAS,CI,NY,SU

Fresno: Mono Hot Springs, 6500', near mineral springs, 7/25/53, Raven 5854 (CAS

Poa juncifolia Scribn. subsp. Porteri Keck, subsp. nov.

SASKATCHEWAN.

Wood Mt. Post, 6/10/95, JMacoun 13259 (NY

Moose Jaw, 7/17/96, JMacoun 13262 (NY

Cypress Hills, 6/25/36, JLBolton (US

Farewell Creek, S. of Cypress Hills, 6/27/95, JMacoun 13302 (NY

ALBERTA

White Mud R, 6/22/95, JMawoun13300 (NY , 6/21/95, JMacoun 13301 (NY

Pot Hole Ck, S. of Lethbridge, 7/21/95, JMacoun 13308 (NY

BRITISH COLUMBIA

Valley of Elk R, along Goat Ck, 27 mi. N. Natal, 7/4/41, Weber 2293 (NY

NORTH DAKOTA

Stutsman Co.:
Buchanan, slightly alkaline soil in coulee, 6/16/11, Bergman 171 (NY

SOUTH DAKOTA

Hot Springs, 3500', 8/3/92, Rydberg 1146 (NY

Elk Canyon, 4-5000', 6/29/92, Rydberg 1146 (NY Meade Co.

MONTANA

Fergus Co., 7/3/01, Spragg (RM

Meagher: Black Hawk, mdw, 8/1/96, Rydberg 3272 (NY

Castle, 8/1/96, Rydberg 3243 (NY

Elk Mts., nr. Castle, 8/1/96, 6000', Flodman 151 (NY,RM

Park: Springdale, 6/14/03, Lunell (RM

WYOMING Sheridan (or Johnson): rolling plains betw. Sheridan & Buffalo, 3500-500 ft, 6/15-7/15/00, Tweedy 3697 (NY

Crook: 5 mi. S. of Sundance, 6/14/44, Porter 3434 (RM

Weston: 10 mi. S. of Newcastle, 7/15/44, Porter 3425 (RM

Goshen: Raw Hide Butte, 9/5/92, Buffum 5115 (NY

Platte: Chugwater, 6/26/01, ANelson 8253 (RM

Laramie: Pine Bluffs, 7/7/97, ANelson 3644 (RM

Albany: Chimney Park, 8/1/01, ENelson 462 (RM

Chug Creek, open flats nr. the creek, 6/29/00, ANelsnn 7326 (RM

Poa juncifolia Scribn. subsp. Porteri Keck - page two

WYOMING. Albany Co. continued: Pole Mtn. region, bunchgrass in dry gravelly soil, 8400', 7/6/43, CLPorter 3249 (NY, ~~type~~

dry roadside betw. Albany and Keystone, Medicine Bow NF, 9200', 7/31/47, CLPorter 4334 (RM

Carbon: Saratoga, 6/91, Buffum (RM

Sweetwater: Beaver Basin, Colorado line, 7/22/97, ANelson 3806 (RM

North Fork Vermillion Creek, 7/19/97, ANelson 3766 (RM

Granger, Ham's Fork, 7/30/97, ANelson 3891a (RM

COLORADO

Clear Lake: Clear Lake, Georgetown, 8/17/95, Rydberg 2376 (NY

Poa nevadensis Vasey ex Scribn.

MONTANA

Lewis and Clark: Helena, 6/27/88, FDKelsey (NY lig. 3.7 mm, acumin. good nev

Madison: Spanish Basin, Madison Range, 6000', 7/13/96, Flodman 145 (NY
(typical nevadensis with ligule 4.1 mm. long)
just below Lower Brandon L, Tobacco Root Mts, edge of stream, 7/29/47, Hitchc. 17006 (UW

Park: 1 mi. E. Cooke, open lodgepole timber, 7800', 7/29/51, Porter & Rollins 5792 (RM

WYOMING. Park: clayey mdw at Clay Butte, nr Beartooth Butte, 9000', 8/18/51, Porter 5923 (RM

Teton: dry open slopes, mts N. Buffalo R, Teton For. Res. 8/7/01, Merrill & Wilcox 417 (NY, RM toward ampla; not [illegible] nor ampla nor juncif.
dry bank of mt. pond above Leigh's L, Teton Mts, Jackson Hole, 7000', 7/26/01, Merrill & Wilcox 356 (RM
do do, 7/25/01, 325 (RM, NY

Teton: Buffalo Fork, 7500', 7/97, Tweedy 20 (NY
in cottonwoods along Snake R, Jackson, 7/13/01, Merr & Wilc 262 (RM - ampla @ SU
~~Yellowstone Lake, 8/[illegible]/97, 7500', Rydberg & Bessey 3664 (RM~~
Yellowstone NP: 1888, R. S. Williams (NY ; 8000-9000', 8/84, Tweedy 279 (NY

Albany: nr. Big Hollow, 15 mi. W. of Laramie, 7500', 8/6/44, Porter 3475 (CI,RM

Carbon: Encampment, river bottoms, 7200', 7/15/01, Tweedy 4347 (NY slender like juncif., but lig. rather long; lvs. ± scabrid
like this but not scabrid
Sandstone R.S, 8/8/44, Porter 3482 (RM

Sublette: Piney and Beaver creeks, 8000', 7/22-31/00, CCCurtis (NY
nr. Green River Lakes, 8000', caespitose, edge of lodgepole pine woods, 7/19/4[illegible]

Sweetwater: 10 mi. N. of Pt. of Rocks, along dry run, 6/20/01, Porter 5095 (RM

COLORADO: Merrill & Wilcox 32 (RM scabrous lvs., looks like juuncifol.

Co.?: without other data, Hall & Harbour 674 (US

Larimer: 8 mi. W. of Hebron, Buffalo Pass road, 9000', 8/15/98, along irrigating ditch in mdw, frequent in mdws, Shear & Bessey 1466 (US
North Park, along the Michigan, 8/8/99, HerbStateAgricColl 3752 (RM,NY

Routt: Buffalo Pass Road, Park Range, 8/12/98, Shear & Bessey 1442 (C,US
(rather dry low ground among aspen, 8000', a dry situation form, forming small mats)
Do., foot of Park Range, nr. sawmill, dry burned timber land, very dry soil, 9000', 8/14/98, S. & B. 1470 (US
Red Dirt Divide, Steamboat Springs Road, rich wet somewhat shady gulch, ca. 8000', 8/1/98, Shear & Bessey 1379 (US
Steamboat Springs, dry open ground, forest, rd. to Buffalo Pass, Swallen 1372 (US
grassy mdw, Rabbit Ears Pass, nr. Steamboat Springs, 8/9/28, Swallen 1375 (US
Columbine Lake, meadow, surrounded by aspen, 8900', 7/23/37, Johnson 786 (US
nr. Columbine, 7500', 7/11/38, Bridges 1252 (US

Eagle or Pitkin: Holy Cross Nat'l. Forest, shallow loam, 8/17/23, Hunter (US

El Paso: along track at D & RG depot, Manitou, 7/23/06, Hitchc. 1769 (US

Grand: Sheephorn Divide, moist rich soil, 9000', 9/1/98, Shear & Bessey 1550 (US

Gunnison: Gunnison, 7700', 7/29/96, Clements 238 (NY a peculiar form like the juncifolia from nearby Iola except for long ligules to 4.5 mm. and the lemmas puberulent. = juncifolia ssp. gracilenta

NEW MEXICO.

west. Socorro: Fitzgerald Cienaga, 7/12/05, Wooton (US 2 sheets

Poa nevadensis Vasey ex Scribn. - Page Two

Merrill & Wilcox 141 (NY RM do.do.do. 140 (RM

IDAHO Fremont: common in bunches, dry rocky soil, nr. the river, St. Anthony, 7/4/01

Blaine: Tikura, acres, in natural mdw, 4500', 7/22/11, Nelson & Macbride 1305 (M, KC, RM

Oneida: 12 mi. NW. of Holbrook, 5600', sagebrush, 6/18/41, Hull 259 (NY

Canyon: Emmett, wet saline flats, 3000', 6/9/11, Macbride 867 (C,F,M,Minn,NY,RM

UTAH

Co.?: open slope, Mill Ck RS, 9000', 7/15/26, Hitchcock 23090 (US the glaucous filiform involute short-lvd type

Salt Lake Co: Salt Lake City, 6/9/80, Jones 11434 (CAS
Alta, Wasatch Mts, 11,000', 7/31/79, Jones 1124 (F

Iron: Modena, sandy bottoms, 6/2/02, Goodding 1015 (C,F,NY,RM

Sevier: nr. Fish Lake, 9000', 8/11/94, Jones 5826 (C,RM

Southern Utah, Northern Arizona, etc. 1877, Dr. E. Palmer 474 (NY, 2 sheets, isotypes of P. nevadensis Vasey ex Scribn. The description of nevadensis was obviously based on this collection; it matches well, the lemmas are very scabrous from tip to base, the lowermost hairs approaching crisp-puberulent, but not that in the sense that Canbyi is crisp-puberulent, i.e. t is is nevadensis as historically understood (#492 was collected on Mt. Trumbull, see below in Arizona. 474½ Pf and long! Red Cr. prob. Mojave Co. Ariz. — See Scribner with Pf complex

Washington: Pine Valley Mts, nr. Mountain Meadows, 6000', 6/5/13, Woodbury 5 (RM

ARIZONA.

Coconino: Hart's Ranch nr. Mt. Agassiz, 9/84, Lemmon & Lemmon 3151 (C
in a crater lake nr. Mt. Agassiz, Aug. 84, J.G. Lemmon 3151 (US

Mohave: Pipe Spring, 5000', 5/21/94, Jones 5266 (C,NY,SU,RM
one piece at NY has a short rhizome 32 mm. long. lvs. firm! ligule like C (short for nev. but too big for ampla, 2.8 mm. long, acute to acuminate)
(Mt.) Trumbull, 1877, Dr. E. Palmer 492 (NY lvs. soft, but like C (short for nevadensis but too big for ampla) ~~lvs. soft but ligule like C~~

NEVADA
Elko: Jack Ck, 70 mi. nw. Elko, 6500', 6/25/37, Nichols & Lund 191 (NY
Elko: Parks Station, forming hay in mdws, 6150', 8/4/13, Kennedy 4338 (CAS
Jarbidge, wet mdws, 7000', 7/12/12, Nelson & Macbride 2026 (NY
Gold Ck, freq. on dryer lowlands, 6300', 7/24/12, Nelson & Macbride 2091 (Minn,NY,RM
Independence Valley, 2 mi. S. Tuscarora, 6400', moist sand, 6/30/37, Nichols & Lund 263 (NY RCBunn 1579 (RM scabrous involute lvs. like juncii

Humboldt: dried slough of Quinn R, below Camp McDermitt, S13,T47N,R38E, 6/10/46,

Lander: Austin, 1882, Jones (US, type acc. to A.S.H.
rd. betw. Austin and Big Ck, 6400', 7/28/13, Kennedy 4546 (CAS
Battle Mt, 4513', forming native hay in mdws, 7/23/13, Kennedy 4004 (CAS,
do, 6/15/82, Jones (CAS

Eureka: Hickison A.S, 6000', Toiyabe Forest, sage-grass, dry sites, 7/13/39, Cr ne 349 BC (NY

Poa nevadensis Vasey ex Scribn - page three

Lincoln: Calientes, Meadow Valley Wash, 5/23/02, Goodding 930 (C,RM
do, do, steep mtn. side, do, 920 (C,NY,SU almost typical nev., not good scabrella or Sandbergii

Nye: Mud Springs A.S., Toiyabe Forest, deep loam, 7000', 6/25/38, Crane 236(NY
Mohawk R.S., 8000', 6/17/31, JM & MAR Linsdale 207 (CAS
N. Mud Spring, Salisbury Basin, Toiyabe Forest, 5500', 5/22/40, Pearse 254 (NY
12 mi. N. of Reese River R.S., 6500', 6/22/31, JM & MAR Linsdale 340 (CAS

Washoe: nr. Reno, 4800', 6/20/00, Stokes (C
alkali mdws, Reno, 7/20/89, SMTracey (C,F,NY,US
• 5.5 mi. NNE. Steamboat Springs, NW. Virginia City, 4550', 6/22/38, Adams 83 (C, Veg
Glendale, 4500', 6/28/07, Kennedy 1580 (CAS
Mt. spring betw. airport and Swan Lake on Chas. Sheldon Antelope Refuge, n. Washoe Co., 6400', marsh, 6/4/39, Train 2971 (NY probably n.sp., short rhizomes, broad lvs, acute lig. only 2.6-3.1 mm., minutely pubescent lemmas; an odd plant.
Galena Creek, 8000', 8/1/06, Kennedy 1227 (CAS,SU

Mt. Rose, 8500-10,500', 8/4/38, Howell 14202 (CAS det. juncif. by Chase

spring, 3 mi. E. of Chas Sheldon Antelope Refuge Hdq. bldgs, Bald Mt, n. Washoe Co, small pure stands on springy mesa mdw, 6/6/39, Train 2997 ([illegible]

Ormsb : Eagle Valley, 1446 m, 7/7/02, Baker 1272 (C,NY,RM 1267 (C,NY

Esmeralda: [illegible] branch Fish Lake Valley, nr. [illegible] spr, alkaline marsh, 5000', among sedges, [illegible]/26/38, Archer [illegible] ([illegible],SU

Chiatovitch Creek, White Mts, 7500', 6/20/30, Duran in UC set 502 (C,CAS,NY,RM, SU (3 mi. E. of Calif.)

Mineral: above Cory Canyon, E. side Wassuk Range, dry edge of Mdw. 8000', 9/7/38, Archer 6969 (NY lvs. filiform and scabrous like Cusickii but lemmas rounded on back, much longer-lvd. than Duran's Chiatovitch Ck collection, but otherwise similar

Walker Lake (labelled "Mono Co., Calif." 8/17/94, Congdon (SU

Mountain Spring, E. side Cottonwood Ck Canyon, Mt. Grant, 8000', 6/21/40, timbered spring banks, Train 4099 (NY in part, with pratensis

[illegible]GON

Crook: nr. Crooked R. at Prineville, wet sandy soil, 6/22/94, 955 m. Leiberg 309 (C,US

Wallowa )?): summit of Wallowa Mts, 8000', not rare on high ridges, commonly in shelter of trees or shrubs, 8/22/07, Cusick 3215 (US (atypical with keeled lemmas; originally called nervosa, then rhizomata by ASH, but lemmas glabrous, sheaths slightly puberulent

Malheur: [illegible] Ranch, Malheur R, alkaline mdw, 6/20/[illegible], Cusick 1949 (C,F,RM distributed as P. [illegible] Scribn. n. sp. looks like a hybrid with Cusickii
Vance Ranch Hot Spring, S35,T40S,R42E, 6/13/46, RCBunn 1623, 1620 (RM " " " " "
along a creek, [illegible], [illegible], 7/[illegible]/27, Henderson [illegible]223 (CAS

[O]REGON CONT.

Harney: shores of Harney Lake, 1200 m, alkaline flats, 6/23/96, Leiberg 2378 (C,NY

mouth of Emigrant Ck, low ground, 7/9/12, Peck 6526 (SU

mdw 5 m W. of Riley, 7/9/25, Peck 14311 (SU

Steins Mts, 6/2/85, T. Howell (SU  Cusick 2561a (US (nearest this, but lig. not very long and lemmas almost puberulent. Characters of both ampla & Canbyi

Narrows, July, 1902, Griffiths & Hunter 257 (US

bottoms of the Malheur R. 20 mi. E. of Crane, 5/28/27, Henderson 8232 (CAS

wet mdws, often under water, Belle A Ranch, nr. Burns, 6/22/27, H. 8210 (CAS

hill S. of Burns, Harney Valley, 6/23/27, Henderson 8208 (CAS

bottom lands, Alvord Ranch, E. base Steens Mts, 7/1/27, Henderson 8221 (CAS

along a rill, moist soil, Steens Mts, divide above Anderson Valley, 6/6/27, Henderson 8205 (CAS

mdw, Holloway Place, Cottonwood Ck, nr Nevada line, 7/5/27, Hend. 8218 (CAS

mdws. Fox Valley (Co.?), 7/12/02, Griffiths & Hunter 152 (US very narrow lvs

Bedes Butte, 7/25/02, Griffiths & Hunter 32[illegible] (US toward juncifolia

"P" Ranch (Nr. Frenchglen), 7/02, G & H 301 (US

Lake: along Rock Ck, Hart Mt, 7/16/32, Applegate 7714 (SU  7712 (SU

warm springs on Rock Ck, do, saline flat, Applegate 7710 (SU

Lakeview, 6/[illegible]/32, Johnson (SU

nr. Hot Springs, Lakeview, 6/30/27, Peck 15354 (SU
Goose Lake, 5/29/40, Hitchcock 6733 (NY

Klamath: Brookside Ranch, Swan Lake Valley, moist mdw, 7/3/04, Applegate 3142 (SU

do, do, a very common bunchgrass, 7/1/04, Applegate 3114 (SU

Modoc Point, wet mdw, 6/23/27, Peck 1[illegible]146 (SU ampla ligule; sheaths and blades ± scabrous; lemmas very scabrous but not puberulent at base

Keno, Camp Field, 7/7/29, Peck 9371 ([illegible],SU

Co.?: Hot Lake, 6/22/21, Piper (C

Camp Creek, Maurey's Mts, boggy mdw, 7/25/01, Cusick 2699 (C,RM,NY

[CAL]IFORNIA

Modoc: [illegible] Modoc [illegible]o., [illegible]/03, Manning [illegible]2[illegible] (C

15 mi. [illegible] [illegible] [illegible]s, [illegible]800', soil quite wet, 5/28/40, Hitchcock 6725 ([illegible]

CALIFORNIA cont.

Siskiyou: Medicine L, 7/28/21, Eastwood 10932 (CAS perhaps x nervosa

Castle Lake, 7/24/21, Eastwood 10768 (CAS

Lassen: Dixie Valley, July 5, Baker (C

Harvey Valley, 7/6/34, Howell 12459 (CAS,NY

Honey Lake Valley, abundant wet adobe mdws, "red-top", 6/17-24/97, Davy 3316 (C

s.n. (C adobe mdws, De Witt's, do, do, 3318 (C

5 mi. E. Westwood, rd to Fredonyer Pass, 5200', swale at mdw edge, 7/8/44, Keck 5456 (C,CAS,CI,NY

2 mi. E. Westwood, mdw. leading to Mtn Mdw Reservoir, wet sod, 5000', 7/8/44, Keck 5447 (C,CI

Amedee, 4000', 6/23/97, Jones s.n. (US

Plumas: Constantia, RR Station, 6/10/01, Kennedy (C,CAS,RM

1.5 mi. E. Crow, 5500', 6/25/35, Sawyer 153 (C,VEG

N. side Sierra Valley, 2 mi. W. Vinton, 5000', roadside ditch, sandy soil, 7/9/39, Stebbins 2918 (C

Feather R Inn, Mohawk Val, 4450', grassy swales, 7/8/44, Keck 5420 (C,CAS,CI,[illegible] NY,SU

Sierra: Sierra Valley, Lemmon 5461 (SU,US 6/89, Lemmon (C 5/89, do, (C 6/6/89, Lemmon (C

Sierra Valley, 1.5 mi. N. Sattley, Feather R road, 5000', wet mdw with Poa pratensis etc, 7/8/44, Keck 5415 (CI

Loyalton, 6/29/18, Eastwood 7930 (CAS

Placer (?): Lake Tahoe, 7/01, Miss Ora Bongs (US

Ward's Creek, base of Twin Peaks, Tahoe Forest, 2100-2400 m, 8/14/27, Eggleston 21650 (US

Eldorado: Tallac, 6/28/00, Dudley (SU

Mt. Tallac, dry ground, sagebrush, 7000-9500', 8/6-6/08, Hitchcock 3117 (US

do, alt. do, do, Hitchcock 3151 (US

**foot of Cracked Crag, 4 mi. W. of Fallen Leaf, moist open granitic ledges, 8000', 8/30/42, Stebbins 3308 (C**

Fallen Leaf L, 7/27-8/15/06, Eastwood 1054 (CAS,US toward juncifolia

Mono: Mono Lake, Bolander (US said to = Bolander 6113 type of P. limosa S & W, 75 cm. high, lvs to 3 mm. wide, nearly smooth

ALIFORNIA Mono: cont.: **dry alkali soil, edge of mud flats, hot springs 1 mi. SE. Bridgeport, 6750', 6/16/49, Munz 13638 (RM habit of juncif.**
Bridgeport, alkaline meadows, 6470', 7/27/50, Howell 27404 (CAS,NY

Inyo: nr. Bishop, wet ground along roadside, tall grass, not uncommon, but hard to dig out of the stiff wet soil, base missing, 5/30/06, Heller 8354 (CAS,NY,SU

below Rock Creek Lodge, 8000', 8/6/32, Halperin 538 (CAS
**nr. Blue Bell Mine, Inyo Mts., 10,100', 8/11/53, JC & AR Roos 6000 (NY**

San Bernardino: grazed mdw on N. shore Bear Lake, e of entrance rd to Holcomb Valley, S. Bdno. Mts, 6900', July 1926, Quibell 88 (US indigenous here??-ddk

**Los Angeles: in semi-alkaline mud at edge of brackish pool; flats betw. Lancaster and Rosamond, Mohave Desert, 2300', 5/27/51, J. & L. Roos 5055 (NY**

**Inyo: locally common bunchgrass on moist alkaline flats, Amargosa Desert 3 mi. ne. of Death Valley Jct., on Ash Mdws. road, 2050', 5/7/54,** J & L Roos 6076 (NY

Poa Sandbergii Vasey

SASKATCHEWAN SE of Power Dam, edge of prairie, 6/27/47, Breitung 4139 (RM

ALBERTA McKague, 6/29/39, Breitung 200 (NY

Rosedale Camp, prairies, 2200-2500', 6/7/15, Moodie 954 (NY

BRITISH COLUMBIA

Yale: Pritchard, dry open range, 5/14/35, McCabe 2019 (C

18.5 mi E. of Kamloops in yellow pine Douglas fir parkland, do, do, 2042 (C

5 mi W. of Kamloops, dry knoll, open range, 5/9/34, McCabe 1005 (C

5 mi S of Oliver, semibarrens above Osoyoos Lake, 5/4/38, McCabe 4862 (C

jct. Richter Pass and Copaka Rd, the common grass in the Art. tridentata desert, 5/6/38, McCabe 5921 (C

Tulameen River, 5000', summer 1900, JFKemp (NY

Kootenay: Trail, 6/18/02, Macoun 63433 (CAS (could be scabrella)

NORTH DAKOTA

Slope: bench lands, Little Missouri R, Marmarth, 6/4/14, Moyer 428 (NY

SOUTH DAKOTA Rapid City, Red bed foothills, 6/12/27, Hayward 783 (RM grass-yucca asso

Fall River: Hot Springs, 3500', 6/13/92, Rydberg 1147 (NY typical except lemmas appressed-puberulent to above the middle

NEBRASKA

Sheridan: nr. Hay Springs, 1000 m, 6/6-7/01, MacDougal 75 (NY like Ryd. 1147

MONTANA Custer: Miles City, 5/26/12, Piper (RM

Gallatin: Belgrade, 5/31/01, Blankinship (NY

Granite: dry grassland 30 mi E. of Missoula, 3650', 6/20/44, H & M 9114 (CAS

Missoula: nr. Fort Missoula, 3200', 6/2/33, Hitch. 1646 (CI, CAS, SU sandy open field, plants tufted, not stoloniferous

dry plains nr. Missoula, 6/2/38, Rose 173 (NY a robust form

Pattee Canyon, " , 5/31/38, Rose 164 (UW

Lake: Lake McDonald, Mission Mts, 1000 m, 6/23-24/01, MacDougal 386 (NY

Lewis and Clarke: Helena, 5/15/91, Kelsey (SU 6/22/91, Kelsey (NY

Mts. about Helena, 5/20/87, Anderson 624 (NY, SU

do, 6/87, FW Anderson (NY

Flathead: Bad Rock Canyon, S side Flathead R, 3 mi E Columbia Falls, 6/26/42, HT & JM Rogers 1001 (NY

1 mi. S. of Columbia Falls, 6/27/41, HT Rogers 855 (C

WYOMING Sheridan: Dayton, mdws at ft. of Big Horn Mts, 7/6/35, Williams 2367
Sheridan: Wakeley, R83W, T56N, Sec 4, rangeland, 6/9/43, Pfadt 33 (RM
Yellowstone NP: Mammoth Hot Springs, 7/1/99, A & E Nelson 5630 (SU, RM loose white soil of formations

Mammoth Hot Springs, 7/7/02, Mearns 1571 (SU
Johnson: dry hillside nr. Cutler Ck, T56N, R88W, 8000', 7/25/36, LO & R Williams 3110 (RM
Sweetwater: Red Desert, 6/30/20, Hagman (SU — Red Desert, 6/3/97, ANelson 3118
North Vermillion Creek, 7/20/97, ANelson 3780 (RM
Point of Rocks, plentiful on tablelands, 6/12/00, A Nelson 7154 (Minn, NY, RM 6/17/01, Merrill & Wilcox 2 (RM
" " " 6/15/98, ANelson 4758 (RM
Niobrara: 20 mi. N. of Lusk, 6/17/47, Beetle 4458 (C
Goshen: sandy hills 5 mi. SE. Old Fort Laramie, 4300', 6/16/49, Porter 489[illegible] (RM
Fremont: 30 mi. S. of Leckie, 6/29/01, Merrill and Wilcox 67 (C, RM
Platte: Meadowdale Store and P.O., 7/23/43, Pfadt 98 (RM
Laramie: Rt. 85 5 mi. E. of jct. with R[illegible] 87, 5/24/47, Beetle 4416 (C
Fremont: 10 mi E. Sand Draw Oil Field, 6000', 7/6/49, Porter 4958 (RM
Albany: Green Top (Mt, 8100'), 6/29/97, Nelson 3244 (NY, RM drying down, a robust Sandbergii
Laramie Hills, 6/23/94, A. Nelson 42 (RM
Laramie: dry sandy soil 20 mi. E. of Cheyenne, 7/1/45, Porter 3586 (RM

middle E. slopes of Snowy Range, in lodgepole pine woods, 7/3/47, Beetle 4713 (C

head of Pole Ck, 6/19/97, Nelson 3197 (NY, RM

Pole Mt. region, open gravelly ridges, 8500', 7/6/43, Porter 3246 (C, CI, RM
Sublette: Big Sandy, 7/92, Buffum 1109 (RM
Lincoln: Cokeville, 6/11/98, ANelson 4651 (RM

COLORADO

Larimer: Lyon-Estes Park rd, 6/18/36, Silveus 1411 (CAS, US
Cache, Le Poudre and tributaries, Ft. Collins, foothills, 5500', 6/27/96 Pammel 28 (NY
Jefferson: dry hillside, Mt. Morrison, 2120 m, 6/3/21, Bethel & Clokey 4009 (CAS toward Canbyi

top of Castle Rock nr. Golden, 6000', 7/1/85, Patterson (F

Chaffee: Chalk Ck, 5 mi. above St. Elmo, 10000', 7/13/36, Rollins 1362 (C, SU

Mesa: Grand Junction, Book Cliff road, Picea-Abies assoc., 5/18/16, Eastwood 5212 (CAS dry mdw.

Montezuma: Mancos, 7000', 6/23/98, Baker, Earle & Tracy 110 (C
[dry ground (is this possibly Canbyi or? possibly rhizomatous)]

UTAH

Daggett: Flaming Gorge, 6000', 6/2/32, Williams 487 (CAS, RM

Cache: Pine Canyon, 6200', 5/20/32, Burke 2890 (C

College Bench, Logan, 4800', 6/11/33, Maguire 3228 (C

Blacksmith Fork Bench 1 mi. E. Hyrum, 4800', 6/5/32, Maguire 3227 (C

Box Elder: Cold Water Bench, 5200', 5/23/32, Burke 2889 (C

Davis: Sandy, 4500', 5/22/84, Leonard (SU

UTAH. Davis: cont

Salt Lake City, 5/26/83, Jones (CAS'SU, RM

Fort Douglas, 5/16/11, Clemens (CAS

City Creek Canyon, 6-8000', 5/17/84, Leonard (C

Utah: Point of Mountain, n end of co., 5000', 5/20/40, Harrison 377H (C dry open foothills

**Pleasant View, 4700', 5/26/38, Harrison 8347 (RM**

Mount Timpanogas, 6/15/33, Eastwood & Howell 411 (CAS

Sevier: Glenwood, 5300', 5/22/75, Ward 69 (F

Millard: N face Marjim Can., 46 mi W Delta, 6/15/33, Maguire & Becraft 3913 (C sagebrush

Washington(?): Diamond Valley, volcanic craters, 5/16/02, Goodding 836 (RM glaucous like juncifolia

ARIZONA

Mohave: Mokiak Springs, 19 mi. S. St. George, 3000', in Larrea zone, 4/25/42, FW Gould 1643 (NY det as Canbyi by JRSw. Could be scabrella, but has slender habit of Sandbergii (it is like Goodding 920 but even slighter)

IDAHO

Lemhi: sagebrush 14 mi S. Lemhi, limestone shale, 6/23/44, Hitchc. & Muhlick 9225 (SU

Bannock: Nr. Pocatello, 5/22/92, EPalmer 10 (C. , 5/1/35, Davis (RM

Custer: gravelly washes in low hills across river from Challis, 5200', 6/14/44, Hitchc. & Muhlick 8937 (CAS

limestone gravel on benchl nd N side Mackay Reservoir, 4 mi W. of Mackay, 6/13/44 H. & M. 8894 (CAS

10 mi E. of Challis on Dickey rd, 6/13/44, H & M 8917 (CAS,NY,SU,RM very scabrous throughout

Morgan Creek, 6/29/44, H & M. 9352 (UW, NY

7 mi. N. of Dickey, 6500', 6/24/38, Hitchc., Rethke & van Raadshooven 3776 (CAS,SU

open gravelly mdw. above N Fk Big Lost R, 20 mi NE of Sun Valley, 6/7/44, H & M 8801 (CAS, SU

Latah: Moscow, 5/00, Abrams 609 (CI,SU

Viola, 6/26/92, Sandberg 503 (CAS

Nez Perces: 1500-2000', about Lewiston, 4/24/96, Heller 2974 (NY,SU

do, 5/17/92, Sandberg, Heller & McDougal 187 (CAS, US

do, 1800', 5/15/35, Hitchc. & Samuel 2521 (CAS

do, 1500-2000', 5/28/96, Heller 3028 (C do, 5/5/96 H. 3017 (NY

] Clearwater R, 5/14/92, Sandberg 167 (CAS

IDAHO Nez Perces: cont.

Hatwai Ck, 5/17/92, Sandberg 183 (CAS,US
near Lewiston, JHSandberg 164 in 1892 (NY labelled "type" by P.A. Rydb.

Canyon: Falks store, 2200', dry bottom lands, 4/22/11, Macbride 759 (M,SU,RM do,do, 5/17/10, Mcbr. 43 (SU)

Butte: Bear Ck, 14 mi. N. of Leslie, Lost R. Mts, 7300', 6/12/44, H & M 8817 (CAS,SU

Blaine: sagebrush plains 6 mi. N. of Sun Val., 6/7/44, H & M 8795 (CAS,SU

Elmore: 4 mi. N. of Pine, divide N of Dog Ck, 6/4/44, H & M 8699 (CAS

Co.?: "Southern Idaho", 1893, Dr. E. Palmer 85 (NY,US (do,do,do 10 (NY,US nr. Pocatello)

NEVADA

Humboldt: n. end Santa Rosa Range, S. 20,T46N, R40E, 6/11/46, Bunn 1607 (RM
Humboldt: Winnemucca, 5/17/17, Wooton (US
Paradise Valley, 4/30/05, Kennedy 1040 (RM

Lander: Victory Hiway side rd 20 mi E of Battle Mt, 6/10/33, Eastwood & Howell 180 (CAS

Eureka: Emigrant Pass, Victory Hiway, 6120', 6/10/33, Eastw. & Howell 225 (CAS

Elko: amid junipers E of Wells, 6/11/33, Eastw. & Howell 326 (CAS

Victory Hiway betw. Elko and Wells, 6/11/33, E & H 272, 303a (CAS toward Canbyi

White Pine: 15 mi W Ely, 6000', 6/4/37, Moore and Franklin 472, (SU gravel soil, common, typical

Mineral: Olympic Mine, Mina, 5/30/17, Brown (CAS typical

Nye: Summit Canyon, 8000', 6/13/33, JM & MAR Linsdale 910A (CAS
Twin River, 6000, 5/27/32, " " " 761 (CAS
Wisconsin Creek, South Fork, 8000', 6/4/32, do 804 (CAS
Last Chance Creek, 6000', 5/23/32, do. 749 (CAS

Washoe: 5.8 mi E-SE Pyramid, 5800', 6/1/38, Simontacchi 394 (C sage-grass assoc.-toward scabrella

5 mi E of Sparks, 6/8/33, Eastw. & Howell 35 (CAS

1 mi SE Poeville, NW of Reno, sagebrush, 6750', 6/1/38, Tillotson 29 (C

Hunter's Ck Rd, 6-8 mi SW of Reno, among rocks, 6000', 6/23/38, Archer 6076 (SU

Ormsby: King's Can., 1700-2000 m, 6/1/02, Baker 934 (C

E. of Lake Tahoe on Route 50, 5/26/41, Beetle 2724 (CAS,C, toward scabrella

Douglas: Clear Creek, 10 mi SW Carson City, slopes, 5800', 6/20/38, Archer 6039 (SU toward scabrella!

WASHINGTON

Lincoln: Columbia R 5 mi above Grand Coulee Dam, 1290', 5/2/40, Rogers 380 (CAS,SU,US

Spokane: 10 mi E Spokane, 5/19/35, Hitchcock and Samuel 2610 (CAS,SU

Whitman: Steptoe Valley, 4/2/00, Vasey 3003 (US 5/15/00, V.3008 (SU, 5/30/00, V.3015 (US 5/25/01,V.3018 (US

ver)

Poa Sandbergii Vasey - page five

WASHINGTON Whitman: cont.

prairie remnant along highway, 2.5 mi NW of Colton, 5/30/48, Keck 6036 (CI

Wawawai, 4/23/21, St. John 5946 (C dry slope

Pullman, 6/2/94, Piper 1755 (US do, 5/24/02, Piper 3973 (US
do, 6/2/94, Piper 1909 (F

Wawawai Ferry, 5/8/21, St. John, Warren, Cary, Pickett 6089 (C

Almota, moist mossy hillsides, 4/4/94, Piper 1907 (F,US do, 4/29/94, Piper (US

Garfield: open parks in yellow pine 15 mi S Pomeroy, 4800', 5/25/44, Hitchc. & Muhlick 8299 (SU

Okanogan: Oroville, 6/24/11, Jones (CAS,SU

Conconully, 3000', 7/3/11, MEJones (SU

Adams: Ritzville, 1893, Sandberg & Leiberg 184 (NY

Grant: nr. Coulee City, 5/2/31, Thompson 6189 (SU,US rocky sagebrush plains

in the Grand Coulee 7 mi above the Dry Falls, 5/17/40, Rogers 433 (CAS, SU,US

near Coulee City, 5/2/31, Thompson 6190 (SU rocky sagebrush plains

nr. Vantage, sandy drifts along Columbia R, 5/2/31, Thompson 6109 (US

N of Park Lake, Grand Coulee, 4/20/35, Rollins 837 (C sandy knoll

Walla Walla: Waitsburg, 5/18/97, Horner 546 (US

Chelan: open rocky slopes on Lookout Mt. nr. Leavenworth, 5/23/31, Thompson 6524 (US

Kittitas: scabby hilltop nr. hd. Quillawene Ck, 4000', 6/21/04, Cotton 1615 (US

rocky ledges along Cle Elum R, 2000', 5/12/34, Thompson 10492 (CAS

Yakima: Rattlesnake Mts, 1902, Cotton (C,SU

Klickitat: Rockland, 5/5/ 98, Suksdorf 4990 (US

Bingen, Columbia R, 4/29/97, Suksdorf 2832 (SU on low, rocky bank of, typical

do (transient) on bottomland, 4/30/96, Suksdorf 4964 (US

OREGON

Baker?: Snake River near Landing, 5/28/01, Cusick 2528 (C

OREGON cont. Wallowa: Rim of Joseph Creek Canyon, ca. 3 mi. from Flora, 7/1/52 Howell 28532 (CAS,NY

Wallowa: Paradise, 4000', 6/18/00, Cusick 2415 (C,US,RM stony swales

Horse Creek Canyon nr. mouth of creek, 1650', 5/19/97, Sheldon 8088 (US
do, 1700', 5/15/97, Sheldon 8053 (NY

Umatilla: Umatilla, 5/1/82, T. Howell 601 (C,SU sage plains nr.

S. of Ukiah, 1100 m, 6/26/16, Eggleston 12773 (US
Ukiah, W. slope Blue Mts, dry mdw, 4500', 6/24/08, Cusick 3262 (RM
N of Albee, W spur of Blue Mts, 3500-4000', 6/24-25/08, Chase 4803 (US

Morrow: nr. Rock Ck, 790 m, 5/22/94, Leiberg 89 (C wet banks of hillsides
do, 1040 m, 5/19/94, Leiberg 85 (NY

Gilliam: mouth John Day R, 4/7/25, Henderson 5077 (CAS,SU sunny bluffs

Sherman: " " " " 4/8/25, " 5076 (CAS lemmas nearly glabrous at bas

Wasco: The Dalles, 4/22/02, Seldon 9179 (SU
do, in dry hills, 4/7/14, Peck 4794 (US
do, 4/11/03, Lunell (SU, dry hills

Tygh Valley, 5/29/27, Peck 14897 (SU canyon North of, dry slope

10 mi N Wapanitia, 5/29/33, Peck 17391 (SU dry ground

dry slope, Bakeoven Canyon nr. Maupin, 5/27/33, Peck 17311 (NY,SU

Multnomah: trail to Larch Mt., 6/18/27, Thompson 2709 (SU

Grant: Prairie City, 4/23/25, Henderson 5078 (SU "One of the important bunch-grasses in the John Day Val."

17 mi E of Prairie City, 6/18/28, Peck 16046 (CAS,SU moist ground

Mt. Vernon, 4/26/25, Henderson 5079 (CAS,SU hills

Beach Creek, nr. Mt. Vernon, 4/25/25, Henderson 5080 (CAS,SU

Deschutes: nr. Redmond, 6/16/12, Whited 58 (SU common on the desert

Malheur: Barren Val., 1450 m, 6/2/96, Leiberg 2195 (C plains

bluffs on the N. Malheur R, near Beulah, 5/11/27, Henderson 8202 (CAS

sagebrush hills N. of Vale, 5/9/27, Henderson 8204 (CAS

high hills N of Juntura, Malheur R, 5/12/27, H. 8282 (CAS

Vale Butte, nr. Vale, 5/8/27, Henderson 8203, (CAS

field nr. Cow Ck, nr. Idaho line, 5/24/27, H. 8238 (CAS

stony clayey soil 5 mi N of Jordan Valley, 5/5/40, Peck 20565 (CAS

W of Symes Ranch, Owyhee R. Canyon, 4/27/27, Henderson 8231 (CAS

OREGON cont.

Lake: E of Lakeview, 5/29/02, Applegate 3047 (SU foothills

Klamath: Klamath Falls, 4/20/28, Henderson 9357 (CAS,SU
do, 4200', 6/4/46, Rose 46186 (CAS

Harney (?): rocky banks of Malheur R, 20 mi from Crane, 5/28/27, H. 8234 (CAS, US

Harney: high hills nr. Crane, Harney Val., 6/24/27, Henderson 8207 (CAS

Burns, dry flats, do., Henderson 8239 (CAS
1 mi. NW. Princeton, shade of cliff, 7/5/52, Howell 28663 (CAS,NY
10 mi N of Suntex, 5/29/27, Henderson 8201 (CAS

wet adobe, Sheep Camp, Steins Mt., 5500', 6/15/27, H. 8213 (CAS lvs. all short and and basal, otherwise Canbyi type: #8199 perhaps better! as Canbyi! for culms leafy although it flowers early

CALIFORNIA

Modoc: Davis Ck, 3-4500', 5/15/40, Willits 247 (SU rocky hillside

Goose Valley, 5/26/94, Davy (US

Lassen Ck, Willow Ranch, 55/6/40, Willits 217 (SU 3000-4500',

Cedarville Pass, open Abies forest, 6950', 6/29/41, Beetle 2927 (C,US,NY toward Canbyi

12 mi NE of Alturas, rocky slope under shrubs by stream, 5300', 5/27/40, Hitchcock 6752 (SU

Modoc Lava Beds, Modoc Battle Ground 4/24/27, Applegate 5004 (C, SU

Siskiyou: Klamath hills, 4/16/10, Butler 1187 (C

Indian Well, Lava Beds NM, 6/2/35, Applegate 9267 (SU

Yreka, 4/19/10, Butler 1200 (C dry hill

Lassen: bet. Susanville and Janesville, 5/13/30, Kildale & Gillespie 9362 (SU

nr. Lost Lake, Warner Mts., 8000', 6/14/34, Howell 12172 (CAS

Fredonia Pass, nr. Susanville, 5/14/30, Kildale & Gillespie 9379 (SU

nr. Doyle Station, 1290 m, 5/29/11, Eggleston 6731 (US

Sierra: Sierra Valley, 6/87, Lemmon (C

Newman Point, SE edge Sierra Valley (3.5 mi NE of Sierraville), 5300', 6/3/56, Babcock and Stebbins 1626 (C rocky soil with Art. and Purshia

Sierra Valley, 1.5 mi N. Sattley on Feather R road, 5000', dry edges of mdw with Poa Cusickii, Elymus glaucus, Stipa Lemmonii, etc. 7/8/44, Keck 5411 (CAS,CI, SU

Poa Scabrella (Thurb.) Benth. ex Vasey

BRITISH COLUMBIA

Nanaimo: Sproat, 6/24/90, Macoun 83d (11094) and 96 (11108) (US

WASHINGTON

Skagit: Anacortes, rocky knoll, 5/15/38, Hitchcock 3438 (C,SU 5 mi. S. of

Snohomish: Marysville, sand spit, 5/30, Grant (C

Island: Langley, sea shore, 6/25, Grant (SU

Klickitat: nr. Columbus, 4/13/86, Suksdorf 216 (US

low dry ground, Falcon Valley, 6/16/90, Suksdorf 4937 (US 6/21/11, Suksdorf 7262b (US
Falcon Valley, 6/25/92, Suksdorf 4946 (US
do, 6/13/85, low ground, Suksd, 1128 (US

on dry talus at Bingen, 5/29/13, Suksdorf 7678 (US do, dry rocky places, 5/17/09, Suksdorf 6566 (US 5/14/98, Suksdorf 2831 (US

talus slope, Cape Horn, 5/27/20, Suksdorf 10476

Skamania: dry hillside nr. Rands, 4/30/38, Hitchcock & Marsh 3332 (C,SU

Cowlitz: Kalama, 4/85, Drake & Dickson (F

OREGON

Columbia River, Nuttall (NY ex BM as "Poa tenuifolia Nuttall" an isotype OK as scabrella & not multnomae etc.

Wasco: Tygh Ridge, 5/5/33, Sprague (SU

Clackamas: base of cliff by Willamette River, Oregon City, 7/4/08, Chase 4931 (US

do, so, hanging tufts on vertical cliffs, do, Chase 4926 (US panicle very big and open; this plant intermediate to P. multnomae; ligule long.

Oregon City Falls, rocks of the Willamette, 5/30/85, Henderson 1645 (NY

banks of Willamette R, ? Co, June 1880, Thomas J. Howell (US #859~~0~~(?) intermediate to P. multnomae Piper

Tillamook: Trask River, 7/11/82, TJHowell 73 (US open panicle, puber. lvs.

Marion: wet rocks under Silver Creek Falls, 7/11/28, Thompson 4978 (SU,US panicle open ligule 3.5 mm. long & acuminate

Lane: rocky slope nr. summit of Horse Pasture Mt, 6000', above (SE. of) Mackenzie Bridge, Cascade Forest, 7/22/27, Hitchcock 23476 (US

Skinner's Butte, Eugene, 4/23/20, Bradshaw 1411 (SU

Poa scabrella page two

OREGON cont:

Douglas: dry wooded hill, Roseburg, 7/14/08, Hitchcock 2802 (US

Roseburg, 5/5/87, T. Howell (C,US isotypes of Atropis tenuifolia var. stenophylla Vasey ex Beal panicle open

Do, 6/25/87, T. Howell 240 (US panicle narrow like nevadensis

Umpqua River at Roseburg, cliffs, 4/27/14, Cusick 3927 (US

dry mountainside, Roseburg, 5/18/14, Cusick 4042 (US

Grave Creek, 5/21/84, T. Howell (US, GH type of P. acutiglumis Scribn. panicle ± open, lemmas long and acuminate (lowermost over 5 mm. long), very scabrous throughout and moderately crisp-pub at base (GH

Klamath: Tom's Creek, 3000', 5/14/98, dry rocky canyonside, Applegate 2092 (SU

Little Klamath Lake, rocky hillside, 4500', 5/11/98, Applegate 2046 (SU

Johnson Prairie, E. side, dry yellow pine woods, 4000', 6/13/98, Ap. 2438 (SU,US

rocky bank of Klamath R, 1 mi. from Keno, 7/9/20, Peck 9423 (SU,US

1/2 mi. S. of Klamath Falls, ungrazed land nr. highway, 5/11/40, Hitchcock 6385 in part (SU, RM scabr.! some ampla under this no. UW

Jackson: Corral Creek, Cascade Mts, 6/27/25, dry rocky glades in yellow pine forest, Applega 4319 (SU lvs. less than 5 cm. long, = Sandbergii??

Pinehurst, 6/20/27, dry woods, Peck 15040 (SU

Siskiyou Summit, 4600', 6/23/29, Kildale & Gillespie 8261 (SU

Siskiyou, rocky slope of hill above tunnel, 4000', 7/21/08, Hitchc. 2931 (US

do, do, open places in woods, Hitchc. 2927 (US

do, pine woods, Hitchc. 2892 (US 2883 (US

Jacksonville, 7/9/04, Byron Hunter 540 (US

Josephine: Grants Pass, dry wooded slopes, 6/29/13, Peck 6481 (SU

Rough and Ready Creek, 1450', 5/24/33, stony flats among brush, Tracy 12309 (C,SU

Galice, shady rocky bluff of Rogue River, 4/18/26, Henderson 5955 (CAS,SU,RM

Cedar Creek, 5 mi. down Deer Ck. from Selma, open woods, 3/29/26, Henderson 5762 (CAS,SU,RM

**bluffs of Deer Creek nr. Cedar Creek, 5 mi. from Selma, 4/11/26, Henderson 5952 (CAS,SU,RM), ✓ 5959 (CAS,SU,RM**

Tennessee Pass, 4 mi. from Kirby, rocky hillside, 4/20/26, Hend. 5956 (CAS,SU,RM

Josephine creek, 5 mi. from Kirby, serpentine, 4/21/26, Hend. 5958 (CAS,SU,RM

OREGON Josephine: cont.

hot bluffs, E. Illinois R, 4 mi. above Takilma, 4/22/26, Hend. 5953 (CAS,SU,RM

Waldo, rocky hillside, 4/21/29, Kildale 7375 (SU

nr. Waldo on Camp Chicago trail, 4/19/34, Eastw. & Howell 1688 (CAS

Curry: Cuckoo Creek, 6/18/26, Peck 14665 (SU

Iron Mtn. Summit, west slope, 4000', 8/13/47, WHBaker 4921 (CAS

NEVADA

Nye: vicinity of Currant, spring 1916, Georgia H. Bentley (SU

Currant, lower borders of pinyon-juniper, 6/24/30, Keck 594 (SU

Rhyolite, 3/28/47, Ferris 11270 (SU,RM

Washoe: Dinsmore Camp, Hunter Ck. Canyon, 6000', 6/20/07, Kennedy 1632, 1639 (CAS

Alum Creek, dry hillside, rich soil, 7/18/13, 6500', Kennedy 3044 (SU

Newcomb Lake, 6/6/01, Kennedy 1 (SU ? Co.

Clark: Rocky Gap Springs, Charleston Mts, 1400 m, 4/23/41, Clokey 8698 (CAS

CALIFORNIA. Modoc: Egg Lake, 6/13/94, Baker & Nutting (C Baker (C

Siskiyou: Montague, dry volcanic hillsides, 6/9/05, Heller 8007 (C,CI,NY,SU

25 mi. N. of Happy Camp, 6/1/42, Beetle & Stebbins 3449 (CAS

Yreka, 5/22/09, Butler 821 C,SU 5/6/10, Butler 1294 (C,CAS 6/15, Smith (US

Sisson, 6/15/05, Heller 8038 (NY

Shackleford Creek, 4000', dry woods, 6/12/10, Butler 1749 (C,SU,US

Willow Creek W. of Gazelle, 8/21/99, Dudley (SU

Wagon Creek Falls, E. side Mt. Eddy, swampy places, 6/29/20, Heller 13402 (M,SU

Comptons Prairie, N. base Mt. Eddy, 3800', 6/25/19, Heller 13256 (CAS,SU

Shasta Springs, 5/27/23, Eastwood 11962a (CAS

Moffitt Cr., 6/13/09, Butler 831 (C

Caribou Basin, Salmon-Trinity Alps, 6500-7500', 7/24/37, Howell 13403 (CAS

Rocky Peak, Foxtail Ridge, Salmon Mts., Siskiyou Mts. 8/23/01, Dudley (SU

Little Summit, 4700', 6/10/15, Heller 11956 (CAS,SU

S.E. corner of Siskiyou County, [illegible], Howell (CAS

Del Norte: Hazelview Summit nr. Crescent City, 5/25/29, Kildale 9173 (SU, [illegible]

CALIFORNIA cont.

Trinity: Browns Creek, 1914, Yates 363 (C

nr. Weaverville, 1914, Yates 274 (C

nr. Weaverville, 1914, Yates 275 (C

Little East Weaver Creek, 1914, Yates 343 (C

Weaverville, 5/30/31, Kildale 10806 (SU

[Mary Blaine Mt., 6400', 8/3/35, Tracy 14467 (C,SU in gravelly or rocky soil open situation = Canbyi ]

Norse Butte, 8.3 mi. w. Forest Glen, T 1 S, R 7 E, Sec. 7, 4000', 7/10/44 Keck 5480 (C,CI,SU

Shasta: 2 mi. w. Platina on mid. fork Cottonwood Creek, e. bound. Trin. NF, 2900', grassy slope edge yellow pine, 7/10/44, Keck 5463 (CI

Lassen: Black Mt., 5400', 7/11/37, Stebbins and Jenkins 2322 (C

10 mi. W. of Chester, Route 36, 5/2[illegible]/41, Beetle 2794 (C,CAS

Lassen N.F., T33N, R7E, S15, [illegible]0', 7/12/33, Fischer and Johnson F-219 (C

Dixey Valley, 7/3/1914, Baker and Nuttin (C

Plumas: Meadow Valley, road to Quincy, 3[illegible]00', 7/6/39, Stebbins 2598 (C

s. end Lake Almanor, 4400' dry volcanic soil, dormant soon, 7/8/44, Keck 5441 (CI,SU

**Jamison Creek, 6000', 6/27/51, Howell 27637 (CAS,NY ), 5200', 6/28/52, How. 27663 (CAS, NY )**

Red Clover Val., 7/3/07, Heller and Kennedy 8711 (CAS,M,SU

Eureka Peak, 6500-7000', 6/28/51, Howell 27687 (CAS,NY

1 mi. n. Quincy across Spanish Cr., 3500', in yel pine with Elymus glaucus, 7/9/44, Keck 5424 (CI,SU

Red Clover Val., 7/3/07, Heller and Kennedy 8706, (CI,M,SU,US

Lake Center Camp, Feather R. region, 7/1[illegible]/21, Anna Head (CAS

Tehama: bet. Mineral and Paynes Cr., 5/14/30, Kildale & Gillespie 9397 (SU

3 mi. n. Red Bluff, [illegible]/20/16, Heller 12330 (CAS,SU

Sierra: Sierra Valley, 6/16/01, Doten 48 (SU

Sierra Valley, (Lemmon 5460, 5/20/1869,) (C

**Sierra Valley, 6/1880, Lemmon 520 (C**

Sierra Valley, Lemmon 5469 (C

Butte: 10 mi. n. Chico, 210', 4/1[illegible]/17, Heller 12678 (CAS'SU

3½ mi. e. Durham, 3/25/2[illegible], [illegible]ins 1415 (SU

Poa scabrella (Thurb.) page five

CALIFORNIA cont.

Sutter: 1 mi. s, 2½ mi. e. South Butte, 890', 3/22/31, Embree 22 (C

Marysville Buttes, 4/14, Kennedy 4756 (SU

Marysville Buttes, 4/22/26, Ferris 6348 (SU

Placer: Truckee River Basin, 6/25-30/1897, Davy 8259 (C

Emigrant Gap, 6/28/82, Jones 11384 (SU

Emigrant Gap, 5300', 6/19/17, Heller 12728 (CAS,SU

1 mi. W. of Baxter, 5/7/40, Hitchcock 6341 (C,SU

Eldorado: woods, Glen Alpine Ck. nr. Fallen Leaf L, 6000', 6/25/39, HFCopeland (CAS,SU

[illegible] [bet. Shingle Springs and Eldorado, 4/6/16, Heller 12297 (SU [illegible]
[illegible]

Amador:

Elsie's Creek, 2700', 6/3/1896, Hansen 1707 (C

Elsie's Creek, 2700', 6/30/1896, Hansen 1[illegible]70 (C,M

Clinton, 2010', 5/19/1896, Hansen 16[illegible]7 (C

A[illegible]rc. Sta., 2[illegible]0', [=Jackson,] [illegible] 1[illegible]/1[illegible], Hansen 1[illegible]7 (C

nr. Jackson, wet [illegible], 1[illegible]2, Hansen [illegible]30 (C,[illegible]

17 mi. E. Pine Grove, Carson Pass Hwy., 5000', black loam, semi-shade, 7/1/33, Wolf 5137 (CI

Calaveras: 4 mi e. of Avery, 5/21/27, Stanford 420 (SU

Mokelumne Hill, Blaisdell (CAS

Calaveras R.S. nr. Avery, 1000 m, 5/26-6/8/13, Eggleston 9205 (US as ~~neva~~ nevadensis because of smooth lemmas

Camp Baxter, N. Fk. of Stanislaus R, 5600', 6/28/30, Jussel (CAS ;do, 6/27/30 (CAS with glabrous lemmas)

nr. Avery, 35[illegible]0', [illegible]/23/21, Tracy 5755 (C

San Joaquin: Tracy, [illegible]/25/03, Baker 2776 (C

E. end of Corral Hollow, 4/7/35, Eastwood & Howell 2092 (CAS

San Joaquin River Bridge W. of Ripon, 3/18/35, Hoover 380 (C

Hospital Canyon, 4/5/30, Stanford 1237(CAS

Stanislaus: 4 mi. S. of Oakdale, 3/1[illegible]/35, Hoover 321 (C

Arroyo del Puerto, 250' 3/28/35, Sharsmith 1575 (C

Arroyo del Puerto, [illegible]0', 4/21/35, Sharsmith 1756 (C), 1[illegible]3 (C[illegible]), 1[illegible] (C

Arroyo del Puerto, [illegible]0', 3/29/35, Sharsmith 1641 (C

Poa scabrella (Thurb.) Benth. - page six

CALIFORNIA Calaveras:cont.

Puerto Canyon, 250-300', 3/23/35, Ferris 9059 (SU

Tuolumne: Indian Cr., 1200', 5/15/19, Williamson 84 (CAS,SU,RM open inflor.

Pikes Peak, 7250', 6/20/37, Quick 1839 (CAS , 1840(CAS

Indian Cr., 1200', 5/15/19, Williamson 82 (SU

Upper Cow Ck, 7200', 6/20/37, Quick 1826 (CAS

nr. Bear Creek, 1100', 4/11/19, Ferris 1632 (CAS,SU,RM

Eagle Meadow, 7/17/41, Hoover 5499 (C

Red Hill above Peoria Flat, 1200', ~~April~~ 4/11-16/ 19~~19~~, Ferris 1603 (CAS,SU

N. side of Pigeon Flat (Middle Fk. Stanislaus R. at 6000 ft.), 6/15/41, Hoover 5354 (C small like incurva

nr. French Flat, 1350', 4/11-16/1919, Ferris 1547 (SU,CAS

~~nr. French Flat, 1350'~~ do, do., 4/25/19, Williamson 2 (CAS,SU

2 mi. W. of Jamestown, 4/1/23, Abrams 10027 (SU,US lemmas nearly smooth, called nevadensis

1 mi. W. of Keystone, 4/1/~~19~~23, Abrams 10067 (US , 10045 (SU

~~1 mi. W. of Keystone, 4/1/23, Abrams 10045 (SU~~

Cow Creek, 6500', 7/5/44, Quick 44-43 (CAS

1 mi. ne. Italian Bar, 2600', 4/15/36, Belshaw 1871 (C

½ mi. w. of Chinese Camp, 1350', 4/14/36, Johannsen 826 (C

Upper Piute Trail, 7/24/34, Bartholomew (C

Mather, 1400 m., 7/25/37, Clausen 1546 (C,CI,SU

Mather, 4600', 5/30/31, Keck 1118 (CAS,SU

Mather, dry slope above meadow, 8/10/43, Hiesey 471 (CI

Mather, 1400 m., 6/3/31, Keck 1197 (CAS,SU

Mariposa:

Yosemite Valley, 4000', 6/18/11, Abrams 4366 (SU,C

Yosemite Valley, 5/00, Bioletti 10, (C

nr. Tenaya Creek, 4000', YNP, 5/22/34, Carter 545 (C

El Capitan summit, 7600', 7/6/11, Jepson 4359 (US

nr. top Yosemite Falls, [illegible]', YNP, 5/24/34, Carter 57[illegible] (C

CALIFORNIA Mariposa: cont.

Blochmans Ranch, 4/17/15, Eastwood 4228 (CAS

3/4 mi. sw. White Rock, 775', 4/14/35, Yates 5126 (C

nr. Chowchilla Mt, 7/19/44, Quick 44-08 (CAS

Darrah, 5/1886, Congdon (SU

Cathay Valley, 4/19/15, Eastwood 4349 (CAS

Madera: N. Fork S.R.E. Plots, North Fork, 5/26/34, Bacigalupi 2286 (SU
Mono: 3 to 5 mi. E. of Topaz, 5/20/15, Bolton (CAS immature, perhaps sandberg.
Merced: Pacheco Pass, 1700', T 10S, R 7E, 4/2/34, Short 34 (C
Kings: Kettlemen Hills above Avenal, 3/23/40, Hoover 4274 (C
Fresno: Junction n. and s. Forks Kings R., 5/1/23, Duncan (SU

Big Creek, 5000', 6/26/26, Klyver (SU

Carlson's Mill nr. Big Creek, 6/11/26, Klyver (SU

Alder Springs, 6/13/26, Klyver (SU

Pine Ridge, 5300', 6/15-25/00, Hall and Chandler 72 (C, Minn, SU

Little Panoche Creek, w. Fresno Co., 3/29/35, Hoover 409 (C

Alcalde, 4/1/26, Eastwood 13502 (CAS), 13504 (CAS

n. side [illegible] Grade, 3500', [illegible], Keck [illegible] (CI,[illegible]

Diablo [illegible] 8 mi. [illegible] of Coalinga, 4/13/41, Beetle 2675 (C,CAS,US

(OVER) Fresno, 3/30/35, Hoover 472 (C

Tulare: Dillon's Forest, 4/6/00, Dudley (SU

below Oak Grove, Mineral King Road, 2800', 5/23/25, Bacigalupi 1214 (SU
White Chief Trail, nr. Mineral King, 8500-9000', 7/17/51, Howell 27829 (CAS,[illegible]
vic. Hockett Mdw., "Grant NP" (Sequoia NP), 8-9000', 7/15/1897, Dudley 1903(SU

Hunsaker, 5/1882, Congdon (SU

nr. Milo, 5/5/00 and 5/5/14, Dudley (SU,CI

Alta Meadows, Grant 5334 (C

Kings River, 7/02, Lemmon (C (2 sheets)

Alta Meadows, Grant 5335 (C

Rocky Hill, Exeter, 5/1/30, Parks 0457 (C

Ducor, 3/20/25, Munz 9030 (C

Ducor to Terrabella, 3/20/25, Peirson 5549 (C

Nr. [illegible], 3[illegible] to [illegible]97, [illegible] 3036 (C

Inyo: foothills s. of [illegible], [illegible]1/06, Heller [illegible]304 (C,CAS,SU

CALIFORNIA. Inyo: cont.

Titus Can., Grapevine Mts., e. of Death Val., 3300', T.13S., R.44E., 3/26/47, Keck & Ferris 5812 (CI

n. fork Hanaupah Canyon, Panamint Mts. 7000', 5/7/32, Munz 12560 (C

foot of Mt. Baldy, Panamint Mts, 8300', 6/21/31, Hoffmann (CAS

Johnson Canyon, Panamint Mts., 5/15/06, Hall and Chandler 7013 (C, NMC

Greenwater Spring, S. of Death Valley, 3800', 4/10/40, Munz 16549 (CAS

Millspaugh, Argus Mts., 6200', 5/18/06, Hall and Chandler 7082 (C

Seep Hole Spring, 7500', 5/12/40, Kerr (CAS

foothills S. of Bishop, 5/21/06, Heller 8303 (CAS, SU

Silver Canyon, White Mts, E. of Laws, 5/7/06, Heller 8188 (CAS, SU

ditto, 5/9/06, Heller [illegible]267 (CAS, SU

[illegible] Peak, Argus Mts, 4/12/30, Ferris 7649 (SU

3 mi. S. of S. Fk. Oak Creek, 7000', 6/2/40, Kerr (CAS

Emigrant Canyon, 2 mi. n. [illegible] Spring, Death Valley [illegible], Panamint Mts., [illegible]200', T. 17S., R. [illegible]E., 3/27/47, Keck [illegible] (C[illegible]

Leadville Pass, Grapevine Mts, [illegible]200', [illegible]/24/32, Coville & Gilman 423 (US
lemmas glabrous, so called nevadensis

Wild Rose Canyon, Panamint Mts, 8200', 5/15/31, R. Hoffmann 2[illegible]2 (US

foothills 4 mi. W. of Lone Pine, [illegible]/28/40, Hitchcock 6258 (SU, RM

Kern: China Grade nr. Oil City, 5/5/05, Heller 7584 (C, CI, SU

"Pah Ute" Peak, 5-6000', 6/1897, Purpus 5514 (C

1 1/4 mi. n. McKittrick, [illegible]00', 5/1/37, Yates 6523 (C

Pozo Creek, 4/27/37, Eastwood & Howell 4031 (CAS

Hobo Hot Springs, Kern R. Canyon, 5/2/27, Abrams 11[illegible]5 (SU

summit of Walker Pass, 5200', 5/13/30, Howell 5008 (CAS

Shepherd's Ranch, vic. Cisses Sta., 6/26/1895, Dudley [illegible]21 (SU

Red Rock Canyon, 5/13/30, Howell 4957 (CAS , 4/11/41, Beetle 2663 (C, US

Antelope Canyon, vic. Tehachapi Pk, 6-8000', 6/1895, Dudley (SU

6 mi. SE. of Monolith, 3/12/41, Beetle 2665 (CAS, C, US

Antelope Valley, [illegible]00', 3/30/37, Yates [illegible]489 (C

Fort Tejon, 5/30/27, Abrams 11[illegible] (SU

Poa scabrella (Thurb.) Benth. - page nine

CALIFORNIA Kern: cont.

bet. Gorman's Station and Fort Tejon, 5/26/1896, Davy 2341 (C

Red Rock Canyon, 3/27/40, C.L. Hitchcock 5817 (SU

Sam Emigdio Ranch, S. of Bakersfield, 3/21/13, Wooton (US

Cuddy Valley, Mt. Pinos, 5900', ~~June 21, 05~~ 6/21/05, Hall 6360 (C

Tehachapi, 5/6/10, Chase 5729 (US lvs. very narrow, only 6-7 cm. long, basal

Tehachapi Mts., 6/18/1888, Lemmon 5467 (C

Mojave, 6/1/17, Jones (C,CAS,SU

5 mi. s. Mojave, 4/6/26, Munz 10093 (C,US lemmas smooth "nevadensis"

Volcanic Hills bet. Rosamund and Mojave, 3/28/26, Abrams 11198 (SU ; do,)

~~Volcanic Hills bet. Rosamund and Mojave,~~ 3/29/26, Abrams 11204 (SU

Manzana, Antelope Valley, 5/9-24/1896, Davy 2465 (C , 2537 (C

vic. Eliz. Lake, Antelope Valley, 5/1-3/08, 40[illegible]', Hall 30[illegible] (C

~~Manzana, Antelope Valley, 5/9-24, 1896, Davy 2537 (C~~

Trinity: Ruth, [illegible], Lu[illegible] 2 (CAS

Glenn: 12 mi. w. [illegible], [illegible]/15, Heller 11[illegible] (CAS, SU blue oak belt

Black Butte, [illegible]/43, Howell 191[illegible]0 (CAS 7/16/44, Howell 19[illegible] (C,CAS

nr. Bennet Spr., Newville-Covelo rd., 3000', 6/3/15, Heller 11941 (CAS,SU yellow pine belt

Plaskett Mdws, 6000', 8/3/43, Howell 18976 (C,CAS, 7/14/44, H. 19780 (CAS, NY

w. Bennet Spr., Newville-Covelo rd., 3500', 6/17/15, Heller 12012 (CAS,SU [illegible]

canyon of Snow Basin Ck, 7/15/44, Howell 19804 (CAS

Colusa: 2 mi. from Stonyford, 4/23/26, Ferris 6583 (SU

w. of Arbuckle, Arbuckle-Rumsey Road, 4/14/17, Ferris 575 (SU

Humboldt Co.: Devil's Hole, Trinity Summit, 6000', 7/17/32, Tracy 10693 (C,SU [illegible]

~~Devils Hole, 6000', 7/17/32,~~ do, do, do, Tracy 10693½ (C

Trinity Summit, 6/2/1899, Davy ~~and~~ + Blasdale 5862 (C

Hoopa Summit, 6/10/1899, Davy ~~and~~ + Blasdale 5658 (C (2 sheets)

~~Hoopa Summit~~ Do, 6/19/1899, Davy ~~and~~ + Blasdale 5660 (C

[illegible]

Hoopa Mt., 3000', 6/13/33, Tracy 12602 (C,SU

[illegible] Cr., w. Hoopa Valley, [illegible], 1000', [illegible]/27, Tracy [illegible]51 (C

CALIFORNIA Humboldt: cont.

Trinity R. Val. nr. Willow Cr., 500', 4/30/22, Tracy 6011 (C

Horse Mt., 5000', serpentine, 6/20/26, Tracy 7613 (C

Grouse Mt., 5000', 7/26/33, Tracy 12911 (C

Kneeland Prairie, 2500', 5/4/13, Tracy 4044 (C

Kneeland Prairie, 6/23/37, Vestal (SU

South Fork Mt., 5700', 7/16-19/30, Tracy 9053 (C

South Fork Mt., Blake Lookout, 5665', 6/7/31, Kildale + Gillespie 10640 (SU

Dinsmore's Ranch, Van Duzen R. Val. opp. Buck Mt., 2500', 6/14/13, Tracy 4120 and 4121 (C

Buck Mt., 5500', 7/31/12, Tracy 3952 (C

Buck Mt., 5500', stream bank, 7/31/12, Tracy 3916 (C

hd. of South Yager Ck, nr. Yager, 6/2/42, Beetle, Stebbins & Tracy 3478 (CAS

Valley S. Yager Cr., 2500', 5/9/26, Tracy 7469 (C

Phillipsville, s. Fk. Eel R., 400', 4/2/27, Tracy [illegible]1 (C

nr. Bell Springs (Mend. Co.) outskirts, 1899, Davy & Blasdale 5353 (US

Mendocino: ½ mi. s. summit at junc. Fort Bragg rd. and Redw. Highway, 4/1/28, Kildale 4389 (SU

nr. Ten Mi. House, 5/8/~~18~~99, Davy & Blasdale 5284 (C, Davy + Blasdale 5298 (C

Anthony Peak, Tehama Co. line, 7/10/41, Eastw. & Howell 9864 (CAS); ditto, 8/8/43, Howell 19132 (CAS,C

Sherwood Valley, 6/3/~~18~~99, Davy & Blasdale 5230 (C , 5209 (C , 5226 B (C

field S. of Bufford Ranch, Potter Valley, 5/5/46, Baker 11366 (CAS

~~Sherwood Valley, 6/3/1899, Davy & Blasdale 5209 (C~~

Potter Valley, 5/20/25, Eastwood 12740 (CAS

~~Sherwood Valley, 6/3/1899, Davy and Blasdale 5226B (C~~

Sherwood, 6/26/08, Hitchc. 2714 (US

nr. Walkers Valley, 5/26/~~18~~99, Davy & Blasdale 5079 (C

Lake: ridge w. of Leesville, 2000', 5/10/19, Heller 13135 (CAS,SU

Dashiells, Mt. St. Helena, May, Eastwood 12845 (CAS

bet. Howland and Lakeport, 5/5/33, Lodge (C

Sawmill Flat, Bartlett Mt., [illegible]', 6/19/45, Howell 21047 (CAS

CALIFORNIA Lake: cont.

Kelseyville, 4/30/33, Holman (C , 4/1/31, Jussel (CAS), 5/2/24, Blankinship (CAS

1 mi S. of Lakeport, 5/11/43, Howell 18019 (CAS

Cobb Mt, 5 mi. n. Hoberg's, damp grassy mdw., 6/3/42, Beetle 3532 (C

1 1/2 mi. w. Bartlett Sprgs., 5/6/28, Abrams 12477 (SU

6 mi. up w. side Bartlett Mt., 5/6/28, Abrams 12415 (SU; 12427 (CI

Cache Cr., 2 mi. w. Hough's Sprgs, 5/7/28, Abrams 12513 (SU

Summit, 5/10/19, Heller 13135 (C

Kelseville (river bottom), 5/5/28, Abrams 12341 (SU

1 mi. S. jct. Highway and Bottle Rock Rd. S. of Kelseyville, 4/26/40, Koch 896 (C

2 mi. s. Lakeport, 5/5/28, Wolf 1921 (SU

6 mi. S. of Lakeport to Hopland, 4/27/40, Koch 903 (C

Highland Sprgs., 5/30/00, Davy 6650 (C

Napa: St. Helena Grade, 5/3/28, Abrams 12231 (SU , 12234 (CI, SU

~~St. Helena Grade, 5/3/28, Abrams 12234 (SU, CI~~

2 mi. s. Hawkins Mount Inn Camp, 5/3/28, Wolf 1814 (SU

Napa Co. traffic officer's country club, 900', 4/32, Taylor (C

Howell Mt. nr. Angwin Cr., 4/30/33, 1500', Tracy 12118 (C

Solano: Vacaville, 5/22/11, 175', Jepson 4241 (SU

Sonoma: 3 mi. s. Healdsburg, 4/9/02, Heller 5249 (F,SU , 5245 (SU

~~3 mi. s. Healdsburg, 4/9/02, Heller 5245 (SU~~

Wagnon Quarry, 5 mi. W. of Sebastopol, 4/13/40, Wagnon 240 (CI det by Swallen as gracillima.

Marin: Mt. Tamalpais, 4/1891, Michener 119 (C

Angel Island, N. side, 5/16/46, Howell 21895 (CAS

Black Canyon, San Rafael hills, 4/11/43, Howell 17897 (CAS), 17919 (CAS, NY)

Tiburon, 4/17/41, Howell (CAS

Tiburon Peninsula, 5/15/43, Howell 18071 (CAS, NY , 18093 (CAS

Big Carson Canyon, 5/2/43, Howell 17931 (CAS

Sausalito, 5/13/43, Howell (CAS

Rifle Camp, Mt. Tamalpais, 5/25/41, Howell 16213 (CAS, NY

CALIFORNIA cont.

Contra Costa: Antioch, 5/3/1893, Eastwood (C

rd. to Sycamore Canyon, Mt. Diablo, 4/26/25, Kennedy (C

Meridan Peak, Mt. Diablo, summit, 3400', 5/18/35, Bowerman 3028 (C

Antioch, 5/3/93, Eastwood (CAS, type of P. californica var. angusta Davy, ined.

Mt. Diablo summit, 5/25/21, Abrams 8031 (SU

Marsh Creek, 6½ mi. E. of Clayton, 3/21/34, Rose 34069 (CAS

Pinehurst, 5/16, Kennedy (C nr. Turtle Rock, Mt. Diablo, 1500' 4/19/35, Bowerman 2859 (C

Alder Canyon, Mt. Diablo, 2400', 5/3/33, Bowerman 1984 (C,CAS

Mt. Diablo (collector?) C, probably Mt. Diablo, Brewer 1070 (NY

Mt. Diablo, also Bowerman 1869 (C , 2960 (C , 1985 (C , 693 (C , 2900(C , 1381(C
1323 (C , 2237 (C , 1140 (C

salt marsh, 3/24/00, Davy 6534 (C ,

Marsh Creek Canyon, 4/11/41, Hoover 4834 (C

Pt. Richmond, 3/24/00, Davy 6540 (C

Pt. Richmond, 3/17/01, Hall 1661 (C

Red Rock, S.F. Bay, 4/19/36, Carter 1121 (C,SU

1 mi. E. of St. Mary's College, 600', 3/23/41, Beetle 1713 (C,US distr. as "Festuca scabella sic!

Berkeley Hills, 4/16, Kennedy (C

Berkeley, 3/18/01, Chandler 826 (C

Berkeley, 5/19/33, Schreiber 809 (C

Alameda: Arroyo Mocho, 10 mi. S.E. Livermore, 4/10/26, Bacigalupi 1299 (SU

Alameda, 4/31/70, Dr. Kellogg (US 32

Arroyo Mocho, 4/10/26, Bacigalupi 1300 (SU

Cedar Mt. Ridge, 4/11/26, Bacigalupi 1313 (SU

Oakland, dry meadows, in handwriting of Bolander?, Atropis californica Munro (NY
isotype!? typical of late spring stuff, lemma very scabrous well above middle

W. Corral Hollow, 5/21/39, Stebbins 2711 (C , 2699 (C 4/26/53, Howell 28929, 28928 (CAS,NY

Oakland, California, Bolander (NY ex Hb. Thurber & labelled Atropis scabrella Thurb.
is the isotype

~~Corral Hollow, 5/21/39, Stebbins 2699 (C~~

Cedar Mt., 2000', 5/28/39, Stebbins 2718 (C

CALIFORNIA

San Francisco: near bay of S.F., wet soil, 1862, Bolander (SU

Lake Merced, 5/16/01, Jones (C May 1901, Elmer 2869 (CAS

1 mi. s. Lake Merced, 5/11/01, Dudley (SU

San Mateo: nr. San Andreas Lake, 3/18/00, Dudley (SU

San Andreas Lake, 4/20/03, Copeland 1920 (C,CAS

Crystal Springs Lake, e. side of lake in rocky ground, open slopes, 5/11/33, Keck 2311 (CI

nr. Summit Sprgs., Kings Mt., 4/4/96, Dudley (SU

Woodside, 5/4/02, Abrams 2408 (SU

Woodside, serpentine, 3/11/00, Dutton (SU

Coal Mine Ridge, 5/1/37, Barry 38 (SU

Menlo Park Reservoir, 3/01, Abrams, 1016 (SU

Searsville Ridge, 4/10/03, Davis (SU

Divide bet. Alpine Cr. and Pescadero Cr., 1000', 3/20/21, Ferris 2174 (SU

vic. Redwood City, 3/16/00, Dudley (SU

Santa Clara:

Stanford U., 4/29/02, Dudley (SU

nr. Stock Farm, Stanford U., 4/17/00, Dudley (SU

Stanford U., 3/7/00, Atkinson (CI,SU

Page Mill Road, 500', 4/21/18, Ferris 766A (SU

Page Mill Road 6 mi. s. Mayfield, 5/21/18, Ferris 773 (SU

Black Mt., Page Mill Road, 4/14/00, Dudley (SU

Below Clarita Vineyard, Page Mill Road, 4/14/00, Dudley (SU

Soda Rock, Stevens Cr., 4/23/21, Ferris 2158 (SU , 2159 (CI, SU

~~Soda Rock, Stevens Cr., 4/23/21, Ferris 2159 (CI, SU~~

Almaden Canyon, 4/18/26, Bacigalupi 1329 (SU

Farwell Ave., Saratoga, 4/17/10, Pendleton 1449 (C

Rose Hill, Purissima Concepcion, Santa Cruz Mts., 3/29/1896, Dudley (SU

Poa scabrella (Thurb.) Benth - page fourteen

CALIFORNIA Santa Clara: cont.

Loma Prieta, 4/7/1894, Davy 569 (C,SU 4/6/94, Davy 534 (US lemmas glabrous

Tuff Hill, 2/1/00, Cannon (SU , 2/22/00, do, (SU

~~Tuff Hill, 2/22/00, Cannon (SU~~

San Jose, cemetery, 4/6/1888, Lemmon 5459 (C

Mt. Hamilton Road e. of San Jose, 5/14/24, Vestal (SU

Mt. Hamilton, 5/21/23, Lang (SU

bet. Hall Valley and Smith Creek, Mt. Ham. Rd., 4/17/26, Bacigalupi 1326 (SU

vic. Smith Creek, w. side Mt. Hamilton, 2250', Sharsmith 625 (C , 626 (C , do, 2000', 1997 (

~~vic. Smith Creek, w. side Mt. Hamilton, 2250', Sharsmith 626 (C~~

~~vic. Smith Creek, w. side Mt. Hamilton, 2000', Sharsmith 1997 (C~~

Grand View, w. edge Hall Val., Mt. Hamilton, 1750', Sharsmith 1077 (C

Seeboy Ridge, Mt. Ham. Range, 2400', 5/3/35, Sharsmith 1948a (C

Packard Ridge, M.H.R., 3000', 3/31/34, Sharsmith 740 (C

Santa Isabella Cr., n. base Mt. Hamilton, 1750', 3/30/34, Sharsmith 677 (C

headwaters Arroyo Mocho, 2500', 4/21/36, Sharsmith 3508 (C

hills above Coyote R., nr. Coyote, serpentine, 4/23/18, Ferris 812 (SU , 816 (SU

~~hills above Coyote R., nr. Coyote, serpentine, 4/23/18 Ferris 816 (SU~~

San Juan Grade, 4/12/29, Wiggins 3402 (SU

Santa Cruz: Little Basin Road, 1500', 4/30/20, Ferris 1999 (SU

Santa Cruz, Anderson (C,CI,SU

San Benito: San Lorenzo Cr. nr. junct. with San Benito R. 2000', 4 mi. NW. Hernandez, 5/1/33, Ferris 8372 (SU,C, ; Keck 2036 (CI

Tres Pinos R. 8 mi. above Paicines, 4/29/21, Abrams & Borthwick 7811 (SU

No. 2 Mine, New Idria Mining Co., Idria, 4/8/28, Ferris 7027 (SU

Above New Idria, 3/31/1899, Dudley (SU

betw. Paicines and Pinnacles P.O., 4/22/33, Howell 11056 (CAS 11057 (CAS

5.4 mi. s. Willow Cr. Sch., rd. to Pinnacles, 5/1/33, Ferris 8353 (SU

The Pinnacles, 3/29/30, Howell 4601 (CAS 4601a (CAS

Hernandez, 6/1/1899, Dudley (SU

1.5 mi. from junction of San Benito-Hernandez road on crossroad to Bitter-water Valley, [illegible]', [illegible], Sec. 32, [illegible] Keck [illegible] (CI

CALIFORNIA

Monterey: s. side San Juan Grade, 4/12/29, Ferris 7491 (C,SU

1 mi se. Jolon on road to Bryson, 960', in compact silt, 5/2/33, Keck 2078 (CI

Big Pinnacles, 4/27/19, Ferris 1722 (SU , 1744 (SU , do, spring, do, 1687 (SU

~~Big Pinnacles, 4/27/19, Ferris 1744 (SU~~

~~spring, Big Pinnacles, 4/27/19, Ferris 1687 (SU~~

Pacific Grove, 4/02, Elmer 3511 (C,CAS,SU

n. fork Big Sur nr. Cienega Cr., Santa Lucia Mts., 5/11/20, Abrams 7383 (SU

King City, 3/24/35, Eastwood & Howell 1956 (CAS

Tularcitos Ranch, Carmel Valley, 5/15/24, Bacigalupi (SU

The Indians, Santa Lucia Mts., 3/30/20, Ferris 1855 (SU

Jamesburg, 5/10/25, Bacigalupi 1094 (SU

Tassajara Hot Springs, 2500', Santa Lucia Mts., 4/26/33, Ferris 8324 (C,SU

Tassajara Hot Springs, 6/01, Elmer 3304 (SU

Pick Place, Big Sur, May-June, 1901, Davy 7488 (C

Tassajara Hot Springs, [illegible]/10/25, Bacigalupi 1125 (SU

Pine Canyon Road, 3 mi. w. King City, 4/8/35, Keck and Clausen 3025 (C,CI,SU

5 mi. S. of San Ardo (Keck obs.)

Nacimiento River, Santa Lucia Mts., May-June, 1901, Davy 7692 (C

3/4 mi. e. Bryson, 5/2/33, Ferris 8455 (C,SU

Pico Blanco summit, May-June, 1901, Davy 7331 (C

N. Fork San Antonio, 1600', 3/27/20, Duncan 91 (SU

N. Fork San Antonio R., 1700', 4/14/23, Ferris 3657 (SU

San Antonio Mission, 4/3/15, Eastwood 4108 (CAS

Pine Canyon road, 1400', 3/26/20, Ferris 1795 (SU

nr. Jolon, 4/3/01, Dudley (SU 4/4/15, Eastwood (CAS, ditto, 4085 (CAS

Cone Peak Ridge, 4750', Santa Lucia Mts., 4/14/23, Ferris 3646 (SU

canyon back of Rich's Cat Hills, 2250', 4/3/20, Duncan 63 (SU

summit of grade bet. Estrella and Parkfield, 3/31/35, Ferris 9151 (C,SU

San Luis Obispo: Hatterwater Val. bet. Cholame and Annette, 3/21/32, Wiggins 5796 (C,CAS,SU

3 mi. s. Paso Robles, 4/13/29, Wiggins 3437 (SU

Old Creek bet. Cayucos and Morro Beach, [illegible]/16/29, Ferris 7659 (SU

Carrizo Plain nr. Painted Rock, 4/29/37, Eastwood & Howell 4133 (CAS

CALIFORNIA San Luis Obispo: cont.

Sta. Lucia Mts., (Barber) 5/7/1899)(C , 5/1/00 (C

~~Sta. Lucia Mts., 5/1/1900, Barber (C~~

alkali flat E. of Cholame, 4/24/48, Howell 24217 (CAS

Paso Robles, 5/1/00, Barber (C , 5/23/99 (C , 5/15/00 (C, SU

~~Paso Robles, 5/23/1899, Barber (C~~

~~Paso Robles, 5/15/00 Barber (C,SU~~

N. end of Carrizo Plain, on black clay with Antirrhinum ovatum, 4/49, Eben McMillan (CAS

Pechoy Islay Ranch nr. Port Harford, 5/5/02, Dudley (SU

10 mi e. Shandon, 5/23/29, Wiggins 3736 (SU

7 mi. e. Pozo, Santa Maria-McKittrick rd., 3/29/35, Ferris 9079 (C,SU

3.6 mi. ne. Huerhuero School, 2000', 5/22/37, Hendrix 74 (C

1 1/2 mi. nw. La Panza Ranch, 1700', 4/2/37, Paterson 658 (C

6 mi. ne. Estrella on road to Parkfield, 5/3/33, Keck 2123 (CI

1 mi. nw. Syncline Hill, 2500', 3/27/36, Yates 5397 (C

1.8 mi. sw. Chimney Rock, 1200', 3/27/37, Nordstrom 1020 (C

10 mi. e. Cuyama Ranch, 2700', 4/3/37, French 916 (C

Santa Barbara: head Pine Canyon, Guadalupe Quad. Sec.-, T 8N, R 35W, 4/3/37, Yates 6729 (C

Pine Canyon, Guadalupe Quad., Sec.-, T 8N, R 35W, 300', 4/13/37, Yates 6528 (C

6 mi. sw. Cuyama Ranch, 2450', 4/6/35, Axelrod 228 (C

2 1/2 mi. sw. Spanish Ranch, 2500', Santa Barbara N.F., 3/30/36, French 759 (C

Big Pine Mts., 6400', 6/10/36, French 822 (C

Roble Canyon, San Rafael Mts., 3400', 5/20/07, Hall 7811 (C

Santa Barbara, Santa Ynez Mts., 5/02, Elmer 4153 (SU,US

Santa Ynez Mts., Santa Barbara, 5/02, Elmer 3789 (CAS, SU

Lobo Ranch, Santa Rosa Island, -/19/29, Hoffmann (CAS

[illegible]ura: Ojai Valley, 4/1896, Hubby (SU , 3/12/96, Hubby 23 (SU , 4/11/96, 37 (SU

~~Ojai Valley, 3/12/96, Hubby 23 (SU~~

~~Ojai Valley, 4/11/96, Hubby 37 (SU~~

[illegible]ta Barbara:
Valle del Medio, Santa Cruz Island, 4/10/31, Howell 6198, 6201 (CAS

CALIFORNIA Santa Barbara: cont.

Pelican Bay, Santa Cruz Island, 4/25/30, Abrams and Wiggins 10, (C,CAS,SU

Smugglers Cove, 5/14/27, Hoffmann (CAS

Lady's Cove, Santz Cruz Island, 800', 3/27/32, Hoffmann (C

Mesa above Christy's, w. end Santa Cruz Is., 3/21/32, Hoffmann (C

Ventura: Griffins, Mt. Pinos, 7/02, Elmer 4185 (NY, SU 5/16/23, Hart (CAS

Pratt Trail, ~~Ventura Co.,~~ 5/27/45, Henry M. Pollard (CAS

Griffins, Mt. Pinos, 7/02, Elmer 4184 (SU

San Antonio Creek Road, 8/25/45, Pollard (CAS

Seymour Creek, Mt. Pinos, 5900', 6/16/23, Munz 6992 (C

Ortega Hill Trail, 5000', 5/4/46, Pollard (CAS

Sawmill Mt., e. Mt. Pinos, 7000', 4/17/34, Sowder 385 (C,SU

burn nr. Kennedy Canyon, 4/13/46, Pollard (CAS

Ventura Valley, 3/28/96, Hubby 25 (US

Chuchupate Camp, w. side Frazier Mt., 7000', 6/3/33, Epling & Wheeler 1821 (C,CAS,CI

Los Angeles: Saugus, 4/03, Grant 5437 (C,SU

Saugus, 3/1/01, Davy (C

Saugus, 5/03, Grant 1016 (SU

Santa Monica Mts., 4/01, Abrams 1475 (CI,SU ,1262 (CI, SU

~~Santa Monica Mts., 4/01, Abrams 1262 (CI,SU~~

Verdugo Hills, 4/01, Abrams 1416 (SU

Mulholland Drive, Santa Monica Mts, 3/6/35, MacFadden 13503 (CAS

Garvanza, 3/22/03, Grant 1524 (CI,SU; Grant 1014 (CI,SU

Hills nr. Inglewood, 3/8/03, Abrams 3105 (SU

Lincoln Park, Pas., 4/405, Grant 6463 (C,SU

Mt. Lowe, 4/11/00, Grant (?) 2424 (SU

Old Mt. Wilson trail, 4/03, Grant 1015, (C,SU, Grant 3438 (SU

summit Mt. Wilson, 6/19/06, Grant 6911 (CI,SU , do.,

~~summit Mt. Wilson,~~ 5500', 6/02, Grant 5101 (C do.,

~~summit Mt. Wilson,~~ 6000', 4/22/00, Grant 1475 (SU ,2479 (C ,5/18/04(SU ,

do., 5500-6000', 6/10/03, Grant (SU , do., 5750'

~~summit Mt. Wilson, 6000', 4/22/00, Grant 2479 (C~~ 5/9/20, Peirson 5366 (C

CALIFORNIA Los Angeles: cont.

~~summit Mt. Wilson, 5500-6000', 6/10/03, Grant (SU~~

~~summit Mt. Wilson, 6000', 5/18/04, Grant (SU~~

~~summit Mt. Wilson, 6/19/06, Grant 6911 (CI,SU~~

~~summit Mt. Wilson, 5750, 5/9/20, Peirson 5366 (C~~

Pasadena 2/27/82, Jones (CAS,SU 4/4/05, Grant 1016-6463 (CAS

Pasadena, 189-, McClatchie (SU

Puddingstone Canyon, moist grassy slope, 4/5/19, Munz, Williams, Street 2433 (SU

Puddingstone Dam, San Jose Hills, 900', 3/26/32, Wheeler 538 (CI

U.S.F.S. Lookout (Baldy Lookout), 6000' to 7000', 6/20/17, Johnston 1355 (C,SU

San Bernardino: San Antonio Mts., 7000', 7/1899, Hall 1269 (C , do, 6700', 1260 (C

~~San Antonio Mts., 6700', 6/1899, Hall 1260 (C~~

Lytle Creek Canyon, 5750', 6/1-3/00, Hall 1439 (C,SU,US

Lone Hill (near Upland), 4/19/19, Munz, Street, Williams 2476 (SU

Lone Mt., 4/15/82, Parish (SU

Cold Creek, foothills San Bernardino Mts., 4/1/83, Parish 1641 (SU

San Bernardino, 5/86 (?), Parish 888 (SU

San Bernardino, 5/10/81, Parish 858 (SU

Mill Cr. Falls, 5500', 6/20/01, Parish 5044 (CI,SU

Seven Oaks Camp, 5000', San Bernardino Mts., 6/11-14/01, Grant (SU

vic. San Bernardino, 1000-1500', 3/22/95, Parish 3827 (C

Near Elsie Caves, San Gorgonio Quad. Sec. 25, T2N, R 1W, 7500', 6/26/37, Yates 6678 (C

Bear Valley, San Bernardino Mts., 6800', 6/24/37, Yates 6618 (C

Bear Valley, San Gorgonio Quad. Sec. 20, T 2N, R 1E, 6700', 6/25/37, Yates 6700 (C

Mojave Desert, 15 mi. ne. Barstow on Garlic Springs road, 0.8 mi. n. 2nd summit, 2800', gravel wash, sun; 4/27/35, Wolf 6516 (CI

1 mi. s. summit of Barstow-Cave Springs road, Granite Mts., Mohave Desert, 3800', dec. granite, sun; 4/28/35, Wolf 6561 (CI,RM

Baldwin Lake, N. end, 6750', 5/15/24, Peirson 4539 (CAS

10 mi. s. of Victorville, 4/14/32, C. L. Hitchcock 12247 (C

nr. Lake Arrowhead, Redlands Quad. Sec. 20, T 2N, R 3W, 5200', 6/24/37, Yates 6599 (C

Victorville, [illegible]/11/22, Clemens (CAS

CALIFORNIA San Bernardino: cont.

Rabbit Spring, 2700', 6/2/01, Parish 4888 (CI,NY,SU

Rabbit Springs, moist edges of alkaline seeps, 2900', 4/26/50, J. & L. Roos

Rabbit Spring, 4/25/15, Parish 9800 (SU 4764 (NY ), 6/3/50, do, 4826 (NY

14 mi. ne. of Barstow, 4/23/15, Parish 9787 (C,SU

dry hillsides N. of Barstow, 1000 m, 4/25/35, Clokey & Anderson 6521 (CAS

Salt Wells Canyon on Searles Station-Trona road, 3/17-24/24, Ferris, Scott, Bacigalupi 3911 (S

Cave Springs (Avawatz Mts), 4500', 4/16/40, Hitchc 6079 (C,SU

Ord Mts. s. of Kane Spr., 4200', 5/1/06, Hall and Chandler 6800(C

1.5 mi. w. Excelsior Talc Mine, Kingston Mts., 4500-5000', 5/14/41, Abrams 14161 (SU

Vic. Bonanza King Mine, e. slope Providence Mts., 3200', 5/21-24/20, Munz, Johnston, Harwood 4131 (C

Providence Mt., 5/30/02, Brandegee (C

Riverside: vic. of Riverside, 2250', 4/26/02, (Box Spr. Mt.), Hall 2973 (C

vic. Riverside, 1200', 4/03, Hall 3785 (C , Feb-Apr., 1908, Wheeler (US ,)

~~Riverside~~, 2/26/05, Reed 671 (C

Elsinore, 4/14/88, Lemmon 5462 (C

Whitewater Canyon 2 mi. n. Banning-Indio road, 3/22/40, Munz 15658 (CAS,SU

West Canyon, Palm Springs, 200 m, 4/18/07, Parish 6141 (SU

1 mi. W. of Banning, 4/18/43, Gould 2153 (C

s. side San Jacinto Mts., 5300', 6/01, Hall 2064 (C,SU , do, 5000', 5/22/01, Hall 1137 (C

Fuller's Mills Mts., 5400', (San Jacinto Mts.,) 6/01, Hall 2257 (C

San Jacinto Mt., 4560', 5/1899, Hall 1143 (C

17 mi. E. of Temecula on hiway 79, 4/5/44, Gould 2235 (CAS

~~s. side San Jacinto Mts., 5000', 5/22/01, Hall 1137 (C~~

Chalk Hill, 5500', San Jacinto Mts., 5/21/99, Hall 1130 (C (2 sheets)

Agua Caliente, 4/1882, Parish 1548 (SU

San Diego: near Cuyamaca Lake, 4750', 5/18/25, Munz 9768 (SU

Fallbrook, 3/23/82, Jones 3096 (CAS

Witch Creek, Alderson (SU

Warners Hot Springs, Apr. 1913, Buttle (CAS 4/10/13, Eastwood 2626 (CAS

CALIFORNIA San Diego: cont.

3 mi. below Dulzura, 4/10/27, Wiggins 2225 (C,SU

Henshaw Dam, 5/11/30, Howell 4824 (CAS 4829 (CAS

San Diego Co., 4/17/89, Orcutt, and 4/90, Orcutt (C

Del Mar, 4/15/88, Lemmon (CAS

1 mi. w. Mt. Springs, 4/12/27, Wiggins 2280 (CI,SU

Jacumba, 4/24/20, Eastwood 9496 (CAS

21 mi. w. Campo, 2/20/26, Wiggins 1815 (CI,SU

Laguna, 6/14/94, Schoenfeldt 3606 (US

Bankhead Springs, 4/13/27, Wiggins 2353 (CI,SU

nr. Foster, 4/03, Hall 3870 (C

San Felipe, 4/18/96, Brandegee (C

National City, 3/19/01, Setchell (C

Chollas (Valley, near) San Diego, 5/26/94, Orcutt 1070 (US, type of P. Orcuttiana Vasey. Typical tall non-anthocyanous scabrella. isotype (SU

Potrero (no county indicated, but likely San Diego, and possibly collected by T.S.Bdg???), April 9, 1892 (US es CalifAcadSciHerb, No. 25, type of Poa capillaris Scribn., not L., which became P. nudata Scribn. A dense-panicled form of scabrella; lvs filiform short and basal like Sandbergii but culms fairly stout and erect. Good scabrella

Orange: Casa Blanca, San Clemente Island, 4/12/23, Munz 6778 (C

mesa summit, San Clemente Island, 4/36, Hurbarger 139 (C

St. Cataline Island?, T. S. Brandegee (C

Baja California. 8 mi. from Rosario on rd. to El Marmol, 3/4/30, Wiggins 4338 (CAS,C,SU,US

Cantillas Mts. 7/5/87 (84?), CROrcutt 1148 (SU

Sacramento" (Sacramento Valley), Hartweg 2035 (NY (exBM), isotype of Sclerochloa californica Munro ex Benth., Pl. Hartw. 342, 1857, nomen nudum. A scabrella with peculiarly short lvs. like typical Sandbergii but long culms with scabrella panicles.
